# Interventions to address unprofessional behaviours between staff in acute care: what works for whom and why? A realist review

**DOI:** 10.1186/s12916-023-03102-3

**Published:** 2023-10-31

**Authors:** Jill Maben, Justin Avery Aunger, Ruth Abrams, Judy M. Wright, Mark Pearson, Johanna I. Westbrook, Aled Jones, Russell Mannion

**Affiliations:** 1 School of Health Sciences, Faculty of Health and Medical Sciences, University of Surrey, Guildford, UK; 2 NIHR Midlands Patient Safety Research Collaboration, University of Birmingham, Birmingham, UK; 3 Institute of Applied Health Research, University of Birmingham, Birmingham, UK; 4 School of Medicine, Faculty of Medicine and Health, University of Leeds, Leeds, UK; 5 Wolfson Palliative Care Research Centre, Hull York Medical School, University of Hull, Hull, UK; 6 Australian Institute of Health Innovation, Macquarie University, Sydney, New South Wales Australia; 7 School of Nursing and Midwifery, Faculty of Health, University of Plymouth, Plymouth, UK; 8 Health Services Management Centre, University of Birmingham, Birmingham, UK

**Keywords:** Professionalism, Unprofessional behaviour, Patient safety, Psychological wellbeing, Psychological safety, Incivility, Organisational culture, Bullying, Workforce, Acute healthcare

## Abstract

**Background:**

Unprofessional behaviour (UB) between staff encompasses various behaviours, including incivility, microaggressions, harassment, and bullying. UB is pervasive in acute healthcare settings and disproportionately impacts minoritised staff. UB has detrimental effects on staff wellbeing, patient safety and organisational resources. While interventions have been implemented to mitigate UB, there is limited understanding of how and why they may work and for whom.

**Methods:**

This study utilised a realist review methodology with stakeholder input to improve understanding of these complex context-dependent interventions. Initial programme theories were formulated drawing upon scoping searches and reports known to the study team. Purposive systematic searches were conducted to gather grey and published global literature from databases. Documents were selected if relevant to UB in acute care settings while considering rigour and relevance. Data were extracted from these reports, synthesised, and initial theories tested, to produce refined programme theories.

**Results:**

Of 2977 deduplicated records, 148 full text reports were included with 42 reports describing interventions to address UB in acute healthcare settings. Interventions drew on 13 types of behaviour change strategies and were categorised into five types of intervention (1) single session (i.e. one off); (2) multiple session; (3) single or multiple sessions combined with other actions (e.g. training sessions plus a code of conduct); (4) professional accountability and reporting programmes and; (5) structured culture change interventions. We formulated 55 context-mechanism-outcome configurations to explain how, why, and when these interventions work. We identified twelve key dynamics to consider in intervention design, including importance of addressing systemic contributors, rebuilding trust in managers, and promoting a psychologically safe culture; fifteen implementation principles were identified to address these dynamics.

**Conclusions:**

Interventions to address UB are still at an early stage of development, and their effectiveness to reduce UB and improve patient safety is unclear. Future interventions should incorporate knowledge from behavioural and implementation science to affect behaviour change; draw on multiple concurrent strategies to address systemic contributors to UB; and consider the undue burden of UB on minoritised groups.

**Study registration:**

This study was registered on the international database of prospectively registered systematic reviews in health and social care (PROSPERO): https://www.crd.york.ac.uk/prospero/display_record.php?ID=CRD42021255490.

**Supplementary Information:**

The online version contains supplementary material available at 10.1186/s12916-023-03102-3.

## Background

Unprofessional behaviours (UB) can be defined as “any interpersonal behaviour by staff that causes distress or harm to other staff in the healthcare workplace” (Aunger J, Abrams R, Westbrook J, Wright J, Pearson M, Jones A, et al:  Why do acute healthcare staff behave unprofessionally towards each other and how can these behaviours be reduced? A realist review, forthcoming). These encompass a range of actions such as incivility, microaggressions, harassment and bullying. Such behaviours persist within healthcare systems globally [1, 2]. Rates differ significantly between countries and contexts; e.g. data from Australia across seven hospitals showed 38.8% of 5178 staff respondents reported experiencing UB on a frequent (weekly or more) basis during the past year, with 14.5% experiencing extreme events such as physical assault [1]. Similarly, in a hospital in Portugal, prevalence of bullying has been found to be 8% [3], and, in Italy, prevalence has been found to be 12.3% for males but 16.4% for females [4]. There are numerous recent scandals in the United Kingdom’s (UK) National Health Service (NHS) that further demonstrate its prevalence. For instance, a 2023 investigation into clinical safety at University Hospitals Birmingham NHS Foundation Trust revealed a pervasive culture of “bullying and toxicity” which had adverse effects on patient care [5]. Similar problems were observed at the East of England Ambulance Service Trust from 2020–2021, which faced monitoring by the Equality and Human Rights Commission due to widespread sexual harassment and abuse [6]. UB has been described as an unaddressed crisis in healthcare (Aunger JA, Abrams R, Westbrook J, Mannion R, Jones A, Pearson M, et al: Unprofessional behaviour between acute healthcare staff: an unaddressed UK crisis, submitted) [7], and one which requires urgent action and further research [2].

Patient care is being jeopardized and staff psychological wellbeing negatively affected by the widespread occurrence of UB [2]. UB in acute healthcare settings can impair communication and concentration, reduce trust in teams, cause a loss of confidence in work ability for staff, and reduce psychological safety [8]. All these factors can lessen the sharing of important patient information, allowing medical errors to go unchallenged and reducing patient safety [9–11]. Illustrating this, a comprehensive retrospective cohort study conducted in the USA examined the data of 200 surgeons and 13,653 of their patients [12]. Results revealed that patients whose surgeons had a greater number of co-worker reports for UB had a significantly increased risk of experiencing surgical and medical complications. Patients whose surgeons had received 1–3 reports of UB within the 36 months preceding the operation faced a 14.3% higher risk of complications, while those whose surgeons had accumulated 4 or more reports faced an 11.9% higher risk [12]. Similar results have been reported when incivility scenarios are simulated [10].

UB between healthcare staff can also negatively impact staff psychological wellbeing [12, 13]. For those who are targeted by or who witness UB, it can result in alienation, depression, and, in severe cases, even suicidal thoughts [14]. This loss of wellbeing can lead staff to take sick leave or leave the organisation or profession entirely [13]. Indeed, bullying and harassment have been cited as one of the primary reasons for the current workforce crisis, with a recent report suggesting 49% of healthcare staff who have experienced UB are seeking another job outside of their organisations or healthcare as soon as possible [15, 16].

Such staff turnover can have significant economic implications [13]. A cautious estimate suggests that the cost of UB to the UK’s NHS amounted to approximately £2.28 billion per year when considering factors such as sickness absence, employee turnover, reduced productivity, compensation and litigation costs. This is equivalent to 1.52% of the NHS’ budget for 2019/2020 [13]. In the USA, replacing staff due to UB, can, for example, cost between $22,000 and $64,000 per nurse [17]; similarly, an estimate of the combined costs for disruptive physician behaviours (e.g. due to turnover, medical and procedural errors) in a 400-bed hospital was found to exceed $1 million.

Prevalence of reported UB varies across different staff members and groups. Data from the UK NHS Workforce Race Equality Standard in 2022 shows that a higher percentage of black, minority, and ethnic (BME) respondents experienced UB compared to their white counterparts, particularly when it originated from managers [18]. Staff members with long-term health conditions or illnesses were also more affected by UB from co-workers [18, 19] and a systematic review including studies looking at prevalence of UB between healthcare staff suggests that more studies find that women are more frequently targets than males [20]. Despite increased attention towards addressing misogyny, racism, and discrimination through social movements like #MeToo and #BlackLivesMatter, there has been little improvement in the experiences of NHS staff between 2018 and 2022. Indeed, discrimination, as reported in the NHS Staff Survey, has been identified as the primary reason for staff leaving NHS hospitals, and thereby contributing to the ongoing NHS workforce crisis [21].

There have been previous attempts to address widespread UB. Several studies have sought to collate and understand interventions to reduce UB in [22] and outside of healthcare [23]. However, existing reviews have focused on only one particular type of UB, such as bullying [22], and their applicability to acute healthcare settings may be limited [24]. Interventions have been implemented in a range of contexts using many different types of approach [22, 25, 26]. Additionally, interventions and the behaviour change strategies they use are often poorly described with insufficient explanation of how and why they are intended to work (see Table 3 below) [26]. Therefore, in this article, we draw on realist methodology to open the ‘black box’ of a heterogenous group of interventions, implemented in complex healthcare systems. In doing so, we synthesise evidence on how interventions to address UB between staff in acute care may work, why and whom they benefit.

## Methods

### Rationale for, and use of, realist methods

Realist reviews seek to understand why an intervention may work in one context but not another. This involves building an understanding of how various contextual factors affect the activation of mechanisms (i.e. changes in participant reasoning) to produce various outcomes [27]. Often, these relationships are not well articulated in the literature, so realist research uses retroductive reasoning (“identification of hidden causal forces that lie behind identified patterns or changes in those patterns” (Maben J, Taylor C, Jagosh J, Carrieri D, Briscoe S, Klepacz N, et al: Care Under Pressure 2: Caring for the Carers – a realist review of interventions to minimise the incidence of mental ill-health in nurses, midwives and paramedics. Health and Social Care Delivery Research, forthcoming)) to unpack this information, drawing on ‘hunches’ as well as inductive and deductive reasoning to ask “why do things appear as they do?” [28]. The aim is to build programme theories depicted through context-mechanism-outcome configurations (CMOCs), representing an understanding of how different interventions and strategies may be used in different contexts. This is done by first developing an initial programme theory representing how and why an intervention may work, before drawing on a wider body of literature to test and refine findings against this initial theory [29, 30].

This review followed the Realist and Meta-Review Evidence Synthesis: Evolving Standards (RAMESES) publication standards [31]. The protocol for this review is published [9], and this article comprises part of a larger realist review which also considers the contributors to UB, the findings of which are forthcoming (Aunger J, Abrams R, Westbrook J, Wright J, Pearson M, Jones A, et al:  Why do acute healthcare staff behave unprofessionally towards each other and how can these behaviours be reduced? A realist review, forthcoming).

### Aim

The aim of this review was to “Identify interventions and strategies designed to mitigate, manage, and prevent unprofessional behaviours and formulate programme theories to describe how, why and in what circumstances these work, and whom they benefit”.

### Review process

The following sections refer to terms commonly used in realist methodology which are further explained and defined below (Table 1).

**Table 1:** Definitions of realist concepts

Realist term	Operational definition
Context	Aspects of the setting in which a programme is implemented which affect how mechanisms are triggered. This can include geographical, social, resource, participant, or other features [30, 32]
Context–mechanism–outcome configurations (CMOCs)	A realist heuristic which enables an understanding of generative causation. This is typically constructed as “an outcome (O) of interest was generated by relevant mechanism(s) (M) being triggered in specific context(s) (C)” [30]
Demi-regularity	*“*Semi-predictable patterns or pathways of programme functioning*”* [30]
Mechanisms	“… mechanisms are a combination of resources offered by the social programme under study and stakeholders’ reasoning in response” [33]
Programme theory	“A set of theoretical explanations or assumptions about how a particular programme, process or interventions is expected to work” (Maben J, Taylor C, Jagosh J, Carrieri D, Briscoe S, Klepacz N, et al: Care Under Pressure 2: Caring for the Carers – a realist review of interventions to minimise the incidence of mental ill-health in nurses, midwives and paramedics. Health and Social Care Delivery Research, forthcoming)
Retroduction	“Identification of hidden causal forces that lie behind identified patterns or changes in those patterns” (Maben J, Taylor C, Jagosh J, Carrieri D, Briscoe S, Klepacz N, et al: Care Under Pressure 2: Caring for the Carers – a realist review of interventions to minimise the incidence of mental ill-health in nurses, midwives and paramedics. Health and Social Care Delivery Research, forthcoming)
Outcomes	“Outcomes are any intended or unintended changes in individuals, teams or organisational culture generated by context-mechanism interactions” [34]

Our review process comprised six main steps as per our protocol [9] (Fig. 2) also outlined below:

- (1) Building initial programme theories. We drew on literature searches of organisational sites including NHS England, King’s Fund, BMA, HCPC, and NHS Employers websites, as well as literature already known to the study team and from the study protocol. Reports were read in depth and data regarding strategies from this step were imported and organised in NVivo12, enabling us to understand the range and scope of strategies used to tackle UB in acute healthcare settings [35]. We then interrogated these sources to build initial CMOCs regarding how, why, and for whom each strategy worked in different contexts. As part of this process, we developed ‘if, then, because’ statements; these were discussed by team members and presented to stakeholders for refinement (Fig. 2). Initial theories are presented in Additional File 1.
- (2) Searching for evidence. From November 2021 to December 2022, we performed systematic, purposive searches for literature on Embase, CINAHL and MEDLINE databases and grey literature on HMIC, NICE Evidence Search, Patient Safety Network, Google and Google Scholar databases, and NHS Employers and NHS Health Education England websites. Unlike in systematic reviews, grey literature is often included as part of realist reviews, because such sources often provide important data for forming programme theories regarding how and why interventions may work in different contexts [31]. Full details of the Search process and Search Strategy are in Additional File 2.
- (3) Article selection. Records were screened according to inclusion criteria, rigour and relevance. Screening of 90% of search results was undertaken by JAA and a 10% random sub-sample was reviewed independently for quality control by both RA and JAA at title and abstract, full text and relevancy stages. Any disagreements were resolved by discussion between JAA, RA and JM. Title and abstract screening was performed using Rayyan.ai software (http://www.rayyan.ai/) and full texts screened using Mendeley (Mendeley Ltd.) [36]. Further, we applied conceptual richness standards to include the most theoretically useful literature using adapted criteria from Pearson et al. [37]. Inclusion criteria were as follows (Table 2):
- Decisions regarding inclusion were based on the criteria (Table 2), relevance (based on both the major/minor criteria below and the ability to inform programme theories) and rigour [38]. Rigour was assessed by evaluating the level of detail describing the methods used, and how generalisable and trustworthy their findings were based on those methods in line with the latest guidance [30, 38].

**Table 2:** Inclusion criteria

Category	Criterion
Study design	Any (including non-empirical papers/ reports)
Study setting	Acute healthcare settings—acute, critical, emergency (and potentially wider, see relevance criteria below). Interventions could be delivered globally
Types of unprofessional behaviour	All as exhibited and experienced by healthcare staff (not patients nor patient to staff)
Types of participants	Employed staff groups including students on placements
Types of interventions/strategies	Individual, team, organisational and policy level interventions. Cyber-bullying and other forms of online staff-to-staff unprofessional behaviour
Outcomes	Included but not limited to a focus on one or more of: staff wellbeing (stress, burnout, resilience) staff turnover, absenteeism, malpractice claims, patient complaints, magnet hospital/recruitment, patient safety (avoidable harm, errors, speaking up rates, safety incidents, improved listening/response), cost
Language	English only
- Our formal criteria for classifying the potential conceptual richness of reports are below. To be included, studies must have:
  - Contributed to the study aims and are conducted in an NHS context; or,
  - Contributed to the study aims and are conducted in contexts with similarities to the NHS (e.g. universal, publicly-funded healthcare systems); or,
  - Been conducted in non-UK healthcare systems that are markedly different to the NHS (e.g. fee-for-service, private insurance scheme systems) but where the mechanisms causing or moderating UBs could plausibly operate in the context of those working in the NHS.
- (4) Data extraction. PDF files for all reports were imported into NVivo12 software (QSR International), which was used as a data sorting and categorisation tool using both inductive and deductive code creation [35, 39]. Codes were created for entries for each identified strategy type to enable ease of theory creation based on relevant data excerpts (Fig. 1). Other important excerpts were extracted separately into a Word document where demi-regularities were identified across studies. Furthermore, key characteristics of included reports were transferred into an Excel spreadsheet.
- (5) Synthesis. We compared, contrasted, reconciled, adjudicated and consolidated different sources of evidence using realist logic of analysis to build an understanding of which contexts affect how interventions work, and why. Identifying demi-regularities (or “semi-predictable patterns or pathways of programme functioning” (Maben J, Taylor C, Jagosh J, Carrieri D, Briscoe S, Klepacz N, et al: Care Under Pressure 2: Caring for the Carers – a realist review of interventions to minimise the incidence of mental ill-health in nurses, midwives and paramedics. Health and Social Care Delivery Research, forthcoming)) across studies enabled us to categorise, by common underlying mechanisms, strategies to address UB. It also enabled us to identify Key Dynamics and Implementation Principles that can impact their success of interventions.
- (6) Testing and refining programme theories. Theories were tested against additional identified literature. At this stage, programme theories from Step 1 were either confirmed, refuted, or newly identified and added to our analysis.

**Fig. 1:**
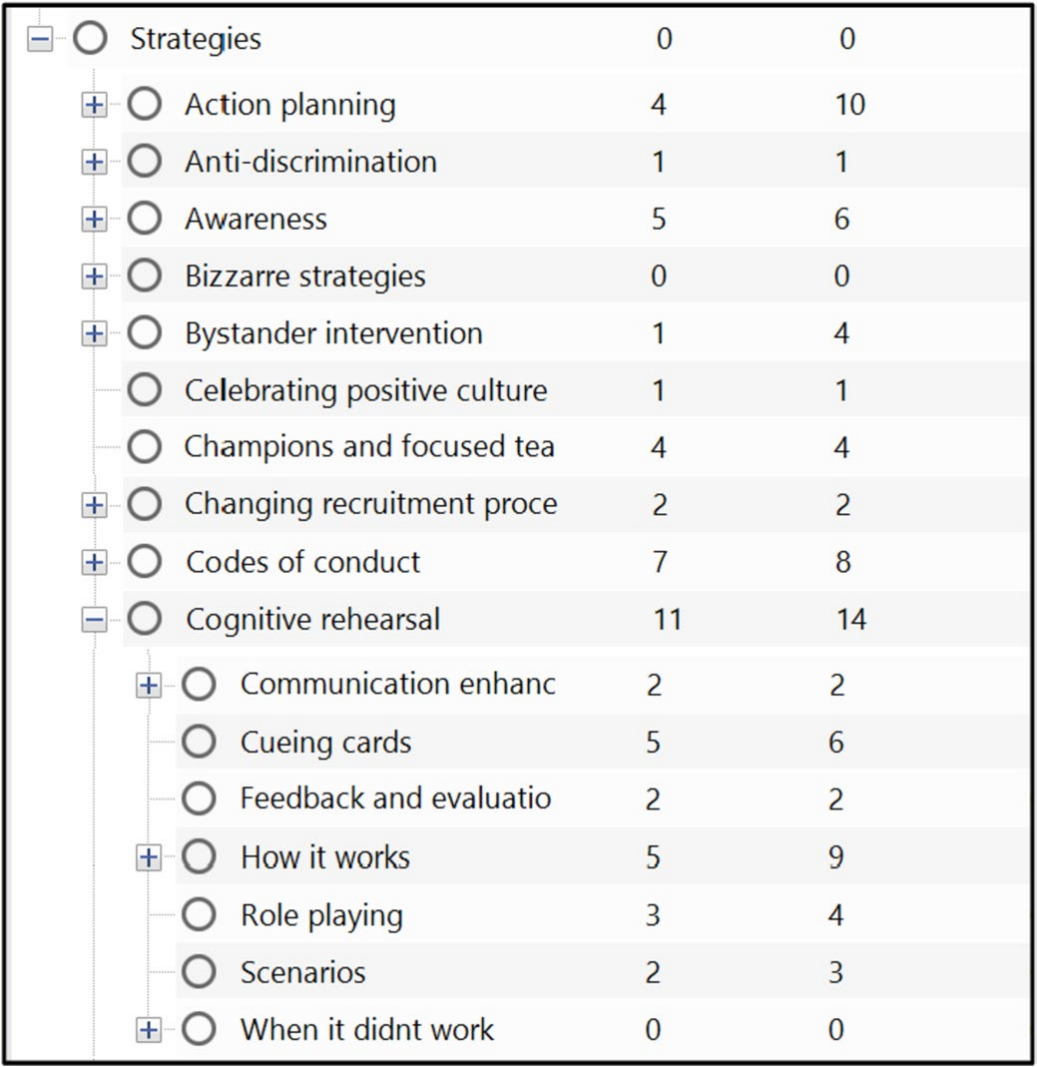
Example code structure in *NVivo*

### Changes to methodology since study protocol

There have been no significant changes since publication of our study protocol [9]. Where flexibility was built into our protocol (e.g. with the relevancy criteria), the reporting of methods in this paper has been updated to reflect the final methods used.

### Stakeholder and patient and public involvement

**Fig. 2:**
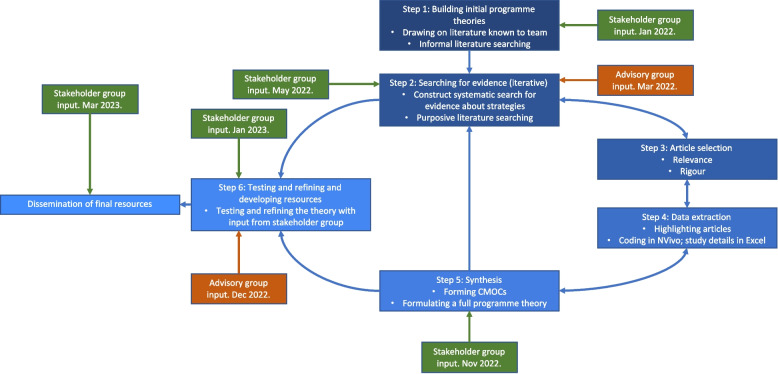
Flow diagram for realist review process. Updated from Maben et al. [9]

Stakeholder feedback was also incorporated at five stages (Fig. 2) using the following process: (1) documenting theory presentation to stakeholders for refinement; (2) documenting suggested alterations; (3) performing purposive searching to sense-check non-aligned suggestions; (4) discussing discrepancies within the team to determine consensus and action taken; (5) re-presenting changes made to stakeholders/group for further sense-checking (e.g. using “you said, we did” summaries at start of each stakeholder group meeting).

Stakeholders and advisors came from relevant backgrounds including patients and members of the public from diverse backgrounds, members of regulatory bodies and trade unions in the UK, and healthcare professionals with lived experience of UB. When compared against the ACTIVE (Authors and Consumers Together Impacting on eVidencE) framework for reporting stakeholder involvement in systematic reviews, our project has adhered to a continuous, multiple-time closed event approach in which stakeholders were able to influence the results of the review [40].

## Results

### Document results

**Fig. 3:**
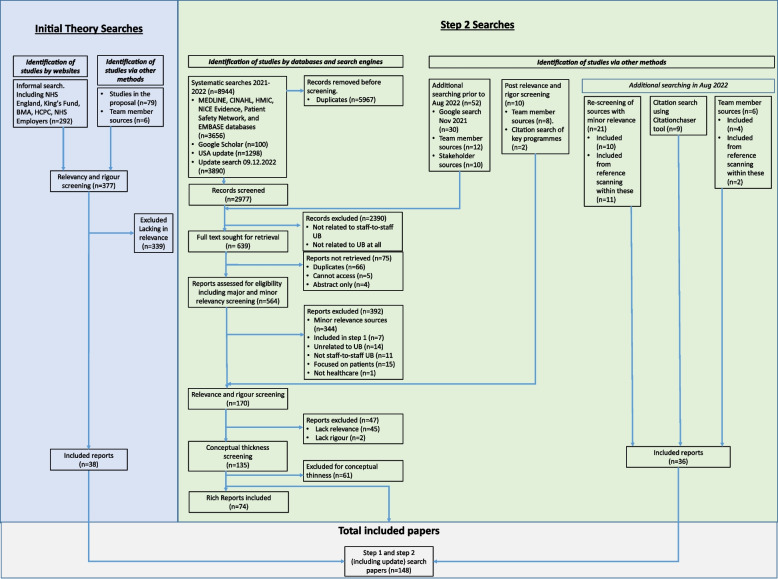
PRISMA-style diagram to depict document selection

We included 38 reports in Step 1 [2, 10, 11, 14, 20, 22, 25, 26, 41–70]. The exhaustive systematic search in Step 2 identified *n* = 8944 records, which reduced to *n* = 2977 when duplicates (*n* = 5967) were removed. Google search, team members and stakeholders identified further reports (*n* = 62). Updated searches in August 2022 resulted in 36 reports being added. After application of inclusion and exclusion criteria, full text and conceptual richness screening, and relevancy and rigour screening, 148 reports were included, comprising 38 for initial theory building and 110 for theory refinement [2, 25, 71–178]. Figure 3 depicts the document selection process and reports.

Of the 148, 42 reported on an intervention in acute care. The other included reports such as editorials, reviews and qualitative pieces were further useful for theory generation, such as by identifying informal strategies to address UB (that were not yet tested in an intervention), and providing information on how UB may manifest, which was useful for answering other research questions in our wider review (Aunger J, Abrams R, Westbrook J, Wright J, Pearson M, Jones A, et al: Why do acute healthcare staff behave unprofessionally towards each other and how can these behaviours be reduced? A realist review, forthcoming).

Analysis of these 148 reports resulted in 55 CMOCs being inferred, tested and refined across areas of (1) intervention types and how they work, (2) strategies to change behaviour, (3) key dynamics and (4) implementation factors which impact how and when interventions work.

### Document characteristics

**Fig. 4:**
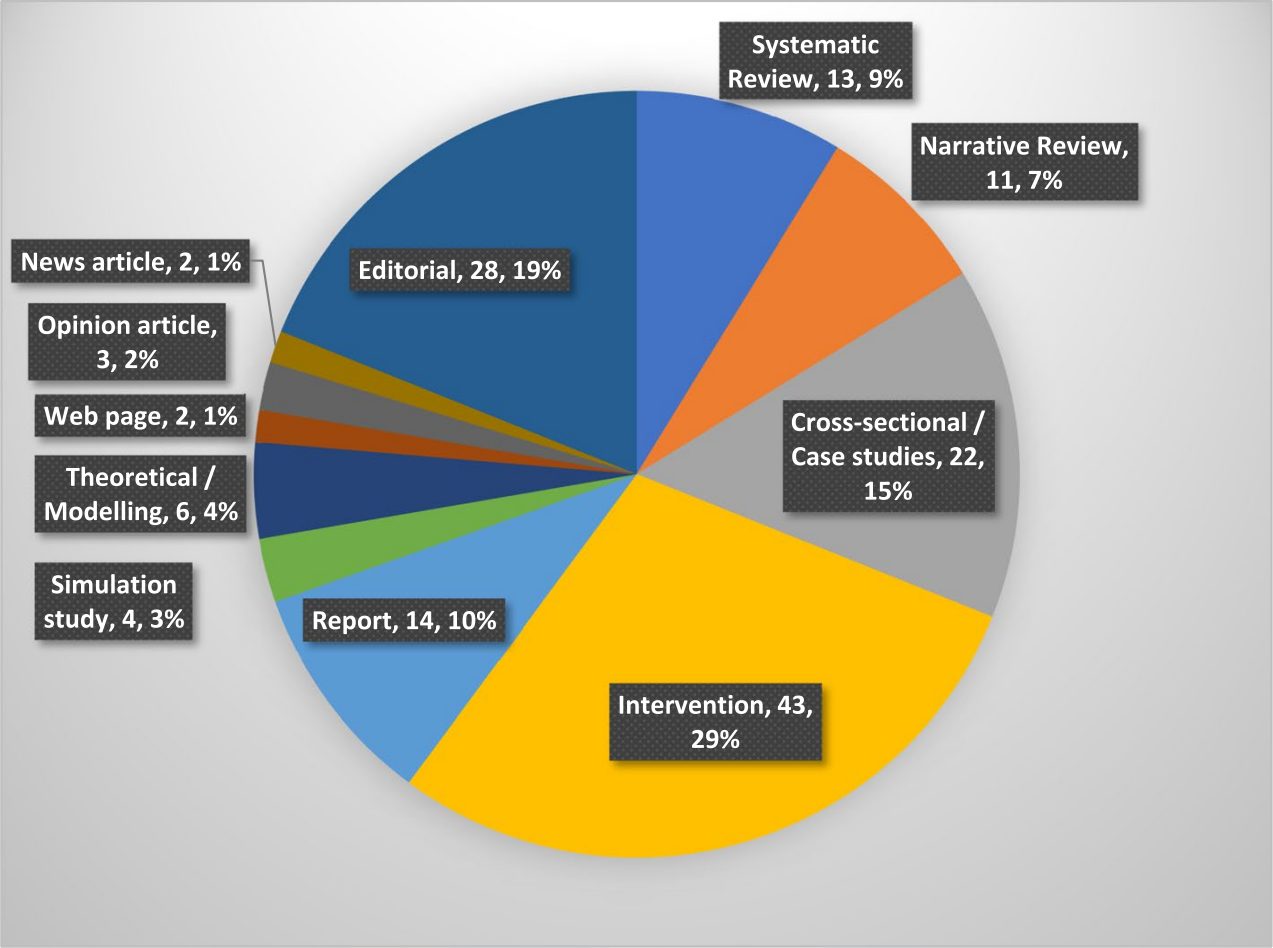
Characteristics of included sources. One intervention paper which informed Step 1 was not included in the realist intervention analysis due to being conducted outside of acute care

Included reports focused predominantly on acute healthcare settings, comprising 37% of included reports. Studies in unspecified healthcare settings, e.g. reports that referred to simply ‘bullying in healthcare’, comprised 38.5%. Over 52% of reports were predominantly focused on the USA or UK. A further 24.3% were not linked to a specific geographical region (e.g. due to being editorials or reviews). In terms of UBs, reports were predominantly focused on bullying (*n* = 47,31.8%), incivility (*n* = 18, 12.2%), horizontal or lateral violence (*n* = 16, 10.8%), or tangential issues such as interpersonal collaboration and culture (*n* = 12, 8.1%) or UB (*n* = 9, 6.1%). Figure 4 depicts source characteristics.

### Interventions and strategies seeking to address UB in acute care

This section outlines the interventions and strategies identified in the literature, and how and why they work.

#### Interventions versus strategies

In this paper, we refer to both interventions and strategies, defined in Table 3 (below).

**Table 3:** Understanding interventions and strategies

Intervention	Strategy
Interventions are defined as “*co-ordinated sets of activities designed to change specified behaviour patterns*” [179]. Interventions are broad, typically comprising (1) the apparatus for delivering strategies, (2) strategies themselves and (3) the evaluation methods assessing their effectiveness [179]	Strategies are components of interventions and comprise the specific ‘active ingredients’ of an intervention [179]. This may include, for example, Behaviour Change Techniques (BCTs). BCTs and related strategies are those aspects within interventions which try to change behaviour in specific ways [179]

#### Interventions seeking to address UB in acute care

We identified 42 evaluations or descriptions of 42 interventions, all of which sought to address UB in acute healthcare settings. Of the interventions; 30 were conducted in the USA [73, 76, 78, 82, 86, 88, 89, 91–93, 95, 100, 101, 106, 111, 116, 120, 122, 125, 127, 129, 137, 141, 143, 156, 164, 165, 172, 174, 177, 178]; five in Australia [74, 108, 126, 160, 162]; two each in Canada [132, 169] and South Korea [83, 94]; and one in Turkey [144], Ireland [100] and Iran [154]. Iran and Turkey were the only low- or middle-income countries to report an intervention. We identified no studies reporting an intervention in the UK or in countries other than those mentioned above (e.g. in developing nations).

We classified the interventions into five types and formulated programme theories regarding how and why these interventions work. These are outlined in Table 4 below.

**Table 4:** Types of intervention identified in the literature and programme theories developed

Intervention type	Intervention design and content	Programme theory
Single-session interventions (*n* = 13) [73, 76, 86, 88, 89, 91, 95, 111, 137, 141, 143, 162, 174]	Single, one-off lectures or workshops to try to change participant behaviour. This can employ awareness-raising strategies such as education about UB, or can be combined with role-playing and other activities intended to enhance the ability to speak up and challenge UB in the moment	**CMOC 1.** If an intervention relies solely on single sessions (C), then while they may raise awareness and knowledge of skills to tackle UB in an organisation in the moment (O1), any planned behaviour change may not be sustained (O2), because they are intended as a one-time delivery of information or training and may be forgotten (M) **CMOC 2.** If an intervention relies solely on single sessions (C), then behaviour and sustained culture change may not happen (O), because it relies on individual behaviour change without any parallel organisation-wide approach (M)
Multi-session interventions (*n* = 11) [82, 94, 100, 106, 125, 129, 144, 154, 156, 172, 177]	These are similar to single-session interventions but rely on use of multiple workshops or lecture-type sessions over time. Most still draw on education and role-playing type activities	**CMOC 3.** If an organisation seeks to implement a multi-session intervention, then, compared to single-session interventions (C), a greater transference of intervention content will occur (O), because it is possible to include more material, and learning is repeated and reinforced, facilitating greater knowledge retention (M)
Combined sessions with other activity interventions (*n* = 6) [83, 92, 93, 108, 116, 164];	These typically draw on single or multiple sessions as above, but also enhance this with non-session-based activities such as implementing an organisation-wide code of conduct	**CMOC 4.** If an organisation implements sessions combined with other strategies (e.g. a code of conduct) (C), then this may increase the spread of knowledge about how to address UB (O), resulting in both systemic change and individual knowledge gain/attitude changes through training or education (M)
Professional accountability interventions (*n* = 8) [74, 78, 101, 122, 126, 160, 165, 178];	These are more complex than those outlined above, relying on a reporting and escalation system. Examples include Ethos and Vanderbilt interventions. These interventions typically combined a reporting system with, in the case of Ethos [74, 126], training to enhance speaking up and role-modelling by leadership or, in the case of Vanderbilt interventions, incorporated championing (i.e. encouraging individuals to role-model and espouse the benefits of the intervention)	**CMOC 5.** If reporting and escalation systems and education about how to use them are implemented to address UB (C), then a clear message of no tolerance is sent to employees (O), because a new and structured route for speaking up and reporting UB is created (M)
Structured culture change interventions (*n* = 4) [120, 127, 132, 169]	These include CREW which offers a flexible package enabling organisations to respond to UB as needed, building upon (1) ongoing action planning to assess which strategies to implement and (2) surveys to understand prevalence and spread of UB. Strategies included training on assertiveness, communication and conflict resolution, as well as management training for leaders and other strategies that help build rapport between staff	**CMOC 6.** If organisations have access to financial and material resources that allow them to address UB in a setting-specific manner with a structured culture change intervention (C), then they will be better able to tailor their response to local UB as it occurs (O), allowing for contributors to be more directly addressed over time (M)

Interventions were evaluated with different study designs. Sixteen used a pre-post design [73, 86, 88, 89, 91, 92, 111, 125–127, 137, 141, 143, 144, 172, 174], three used a pre-post design with a non-randomised control group [120, 156, 162], five other studies used a pre-post design with no control group, but with the addition of follow-up data collection [76, 93, 95, 106, 177], five used a randomised or quasi-randomised controlled trial design [83, 94, 132, 154, 169] and thirteen were descriptive case studies or feasibility studies which did not formally evaluate the effectiveness of the interventions they reported [74, 78, 82, 100, 101, 108, 116, 122, 129, 160, 164, 165, 178]. Only *n* = 18 (43%) of interventions reported using any theoretical framework, of which *n* = 13 (31%) drew on psychological theories, and *n* = 5 (12%) on organisational theories.

With regard to effectiveness, thirteen of the 42 studies were descriptive, or examined only implementation or feasibility issues. Of the 29 studies that assessed intervention effectiveness to reduce UB, the majority (*n* = 23) reported some positive results, while three studies reported no significant change [89, 137, 162] and three reported a negative result [91, 93, 154]. The ‘negative’ results were due to the use of education strategies, whereby people became more active in reporting UB, leading to an increase in reports of UB after the intervention when compared to baseline [91, 93, 154]. Whether an increase in reports of UB is an indication of success or failure is discussed in the “Key dynamics impacting how and when interventions work” section below.

Of the 23 studies which reported some improvement in UB outcomes: nine out of 13 were single-session interventions, seven out of eight were multi-session interventions, two out of three were combined session interventions (although one did not report statistical significance), one of one was a professional accountability intervention, and all four structured culture change interventions reported improvement (see Additional File 3 identify these specific studies).

Studies used a wide range of outcome measures, with the most common being change in the prevalence of UB following implementation of the intervention (*n* = 23). No studies assessed improvements to patient safety or included an economic assessment. Seven studies assessed staff wellbeing or similar proxies such as turnover intention or burnout [83, 94, 108, 137, 156, 164, 169]. Further information regarding document characteristics and intervention study samples, durations, strategies, data collection timepoints, outcome measures, effectiveness and findings are depicted in Additional File 3.

##### Use of strategies in different intervention types

Interventions drew on a range of strategies to attempt to change behaviour or support efforts to do so (further details on the strategies are in the following sections). Most strategies were designed to prevent or reduce UB, except for implementation-aiding strategies, which were intended to support or improve effectiveness of other behaviour change strategies. While not entirely consistent, we identified patterns of use of strategies across different intervention types, which are highlighted in Additional File 4.

#### How, why, and for whom do strategies to address UB work?

The following section outlines the results of our realist analysis, split into sections detailing how strategies work, key dynamics and implementation principles.

We developed 13 categories of strategies by combining them according to common underlying mechanisms for how they are intended to work. For example, social norm-setting strategies work by setting an expectation for accepted behaviour in the workplace. This social norm-setting strategy category includes individual strategies such as championing, positive role-modelling, and codes of conduct. Table 5 sets out the range of strategies identified in this review, arranged by category, and provides an overview to contextualise our programme theories that follow.

**Table 5:** Strategies used to reduce, mitigate or prevent UB and associated programme theories. Evaluated and unevaluated strategies are highlighted separately

**Strategy**	**Description**	**All interventions drawing on the strategy (*evaluated*) or example sources which mention the strategy (*unevaluated*)**	**Programme theories for strategy category**^a^
**1. Direct or indirect approach to instigator (victim, bystander or managers) – Evaluated**			**CMOC 7. Direct or indirect informal approach** When feeling psychologically safe (C1) approaching an instigator directly (R) can provide an opportunity for the instigator to hear about what is perceived as UB and reflect (M1) which may cause them to change their behaviours to be less likely to engage in UB in the future (O1) **CMOC 8. Engaging with formal processes (e.g human resources or reporting system)** When an informal approach either has not worked (C1) or it feels psychologically unsafe to informally approach an instigator (C2) then taking a more formal approach (e.g. using a reporting system (R)) may increase an individual’s/instigator’s perception of risk when behaving unprofessionally (M) and thus reduce their future UB (O)
Informal resolution	Approaching an instigator individually, or their line manager, to try to prompt reflection about behaviour, change future behaviour, or resolve situation	[74, 78, 101, 165]	
Disciplinary action	Process of an individual being identified as problematic and disciplinary action taken against them by managers. Usually combined with reporting system of sorts	[74, 78, 101, 122, 160, 165]	
Peer messengers	Peer messengers deliver reports about UB to potential instigators, on behalf of other people who have been targeted and submitted a report to a reporting system	[74, 78, 126, 178]	
***1. Direct or indirect approach to instigator (victim, bystander, or managers)—Unevaluated***			
* Mediation (unevaluated)*	*Victim and instigator try to resolve their differences with aid of a trained mediator who creates safe environment for discussion*	[119, 130, 131]	
* Changing / softening language (unevaluated)*	*Attempts to change or soften language when reporting mistakes made by clinicians*	[52]	
* Speaking up (unevaluated)*	*Going to a person (e.g. Freedom to speak up Guardian)* [180]* or authority to report the incidence of UB in an organisation, or could be simply to state in the moment that one is uncomfortable with someone’s behaviour. Requires adequate psychological safety*	[77, 108, 122, 130]	
**2. Improving confidence to come forward (victim, bystander)—Evaluated**			**CMOC 9. Improving confidence to come forward** Use of role-playing, cognitive rehearsal strategies, or keeping records as an individual (R) to encourage speaking up about UB (C) can lead to improved self-confidence when coming forward (M) which can lead to the victim speaking up (O1), the instigator reducing their UB (O2) and increased management awareness of UB (O3)
Assertiveness training	Training intended to boost self-confidence and increase people’s ability to challenge UB as it happens	[88, 144]	
Role playing	Similar to cognitive rehearsal, role playing involves practicing resolution behaviours and thoughts with others in group setting. May enhance ability to cope or improve confidence to come forward	[89, 94, 100, 106, 116, 125, 127, 143, 156]	
Cognitive rehearsal	Learning of specific cognitive responses to prepare staff when they encounter UB. Intended to move responses from automatic towards deliberated to enhance coping and reduce escalation [73]	[73, 76, 83, 86, 89, 94, 95, 116, 141, 174]	
Keeping records	Recording incidences of UB and details of the events to provide evidence/improve trustworthiness when coming forward to make a claim against people	[111]	
***3. Improving ability to cope with UB (victim, bystander) – Unevaluated***			**CMOC 10. Improving ability to cope with UB** Use of coping strategies, such as seeking help externally, journalling, reflection, or other individual actions (such as taking sick days) (R) in a situation where one is experiencing UB (C) can lead to an increased ability to cope (M) thereby reducing the impact of UB on the victim’s psychological wellbeing (O)
* Seeking help externally (unevaluated)*	*Looking outside one’s organisation for help with UB, e.g. union representative, regulatory body, or GP*	[98, 119, 139]	
* Journalling (unevaluated)*	*Reflective writing about one’s experience of UB in the workplace may help with coping*	[90, 135]	
* Moving victims (unevaluated)*	*Moving victims away from instigators in organisation*	[25, 70]	
* Individual coping strategies (unevaluated)*	*Various strategies to help improve coping. e.g. taking sick days, hiding emotions/ breathing exercises*	[121, 161]	
* Reflection (unevaluated)*	*Engaging in self-reflection or group reflection to enhance ability to cope, e.g. Schwartz rounds*	[100, 151]	
**4. Understanding prevalence of UB (managers/leaders)—Evaluated**			**CMOC 11. Understanding prevalence of UB** Implementing strategies to understand prevalence of UB, such as performing an audit of an organisation’s culture (R) in an organisational environment where UB is suspected to be prevalent (C), can enable managers to have a better understanding of contributors and where UB is occurring (M1), increase knowledge about interventions that might help (O2) and provide a sense of urgency to tackle UB (M3), leading to better ability to target strategies towards core contributors to UB (O1) which can improve effectiveness at reducing UB (O2)
Survey	Survey to identify the level of UB occurring within an organisation which may help to target or design other strategies	[92, 132, 169]	
***4. Understanding prevalence of UB (managers/leaders)—Unevaluated***			
* Multisource feedback (unevaluated)*	*Similar to reporting systems, but identifies/ investigates individuals from different perspectives—“360-degree” view of individual’s historical behaviour*	[103]	
**5. Improving teamwork (all)—Evaluated**			**CMOC 12. Improving teamwork** Implementing interventions to improve teamworking (R) in an environment with low levels of social support (C) can increase empathy between staff, improving the sense of being supported by others (M1) and improve ability to communicate (M2) thereby reducing chance of experiencing conflict with colleagues (O1) and reducing UB (O2) and increasing ability to cope (O3)
Teambuilding exercises	Generally group sessions which incorporate activities to build a sense of social support and camaraderie	[100, 125, 127]	
Conflict management training	Training to be able to de-escalate situations or avoid escalating them altogether	[93, 132, 156, 169]	
Communication training	Training to enhance ability to communicate in a way which is less likely to be interpreted as, or foster, UB	[92–94, 169, 177, 181]	
Journal club / group writing	Writing as a group, often to reflect on experiences of UB and to build a sense of social support	[129, 172]	
Problem-based learning	Group learning which involves identifying with and attempting to tackle real-life problems. It often involves peer-to-peer teaching	[143]	
***5. Improving teamwork (all)—Unevaluated***			
* Staff networks (unevaluated)*	*Establishing internal or external networks for staff from specific backgrounds (e.g. minority ethnic or female) to share coping strategies/improve social support*	[148]	
**6. Social norm-setting (all)—Evaluated**			**CMOC 13. Social norm setting strategies** When/if leaders are seen to embody and enforce positive behavioural norms (C), then implementing social-norm setting strategies such as a code of conduct or positive role-modelling (R) can signal culture change towards civility (M1) making it socially unacceptable and therefore riskier to engage in UB for instigators (M2) thus increasing the sense of psychological safety (O1) and reducing the likelihood of UB occurring (O2)
Championing	Encouragement for certain individuals to espouse anti-UB values and behaviours, and, sometimes, to act as trusted contacts for reporting UB incidents	[78, 116, 125, 160, 165]	
Code of conduct	Document which clarifies organisational policies on acceptable behaviour and processes to report or otherwise tackle UB	[92, 93, 108, 116, 164, 169, 181]	
Role modelling	Similar to championing, leaders or managers seeking to espouse the behaviours and values they want to encourage in staff	[74, 88, 116, 126]	
***6. Social norm-setting (all)—Unevaluated***			
* Environmental modification (unevaluated)*	*Modifying physical environment can increase awareness of UB (e.g. posters) or reduce discomfort (i.e. more comfortable temperature) which may reduce UB*	[96, 160]	
* Allyship (unevaluated)*	*When an individual from a more privileged background publicly comes out in support of less privileged colleagues and actively furthers their cause*	[68]	
**7. Improving leadership competence and empathy (managers/leaders)—Evaluated**			**CMOC 14. Improving leadership competence and empathy** In an organisation in which there is substantial pressure on organisational leaders (C1), or where leaders have been perceived to engage in bullying-type management practices (C2), implementing training to improve management skills (R) can enhance ability to better understand effects of behaviours (M1) communicate with employees (M2), and enhance empathy for less senior colleagues , supporting ability to manage compassionately (M3), thereby reducing likelihood of leadership directly contributing to UB (O)
Leadership training	Training to improve management or communication styles so that they are less likely to be perceived as using bullying as a management tactic	[101, 169]	
***7. Improving leadership competence and empathy (managers/leaders)—Unevaluated***			
* Reverse mentoring (unevaluated)*	*Enables people in senior positions to learn from and understand issues from perspective of people in less senior roles often from under-represented groups*	[68]	
**8. External pressure on organisations (managers/leaders)—Evaluated**			**CMOC 15: External pressure on organisations ** If there is societal pressure or potential reputational risk for an organisation (R) due to findings of an unsafe culture or prevalence of UB (C) then this can lead to pressure on management to resolve the problem, often speedily (M) which can increase the likelihood of other strategies to address UB being designed, resourced and implemented (O)
Seeking hospital Magnet status	Seeking Magnet status can lead to managers and leaders becoming more focused on addressing a culture of incivility	[116, 125]	
***8. External pressure on organisations (managers/leaders)—Unevaluated***			
* Regulator action (unevaluated)*	*CQC or regulatory body inspection may identify culture of UB, which can place pressure on managers to tackle UB*	[119, 135]	
* Laws (unevaluated)*	*Legislation may place responsibilities on organisations for ensuring equality and employee wellbeing and safety which increases urgency to address UB*	[84, 113, 157]	
**9. Reporting and escalation systems (all)—Evaluated**			**CMOC 16. Reporting systems** In an organisation where people may not feel psychologically safe (C) implementing a reporting system such as those in Ethos or Vanderbilt (R) can provide an alternative means to speak up which feels safer (M1) enabling instigators to be approached and to reflect on their behaviour (M2) which can lead to a reduction in UB (O)
Reporting system	System to report incidences of UB in the workplace. Can be web-based, report to a specific person, or other way. Can be anonymous or not	[74, 78, 101, 126, 160, 165, 178]	
**10. Workplace redesign (all)—Evaluated**			**CMOC 17. Workplace redesign** Adjusting the workplace to give more decision-making power to employees or increasing role clarity (R) in an environment where workplace factors present barriers to performing work tasks (C) can increase a sense of fairness in the workplace (M1) improve psychological safety (M2) improve communication within teams (M3) and improve work engagement and motivation (M4) which can reduce proclivity to engage in UB (O1), increase ability to speak up (O2) and improve psychological wellbeing (O3)
Democratisation of workplace	Reorganisation of workplace processes to drive an increased sense of job control, reduce frustration and reduce hierarchy	[108]	
**11. Improving awareness and knowledge (all)—Evaluated**			**CMOC 18. Improving awareness and knowledge** If employees are engaging in UB unknowingly (C1) or are working in an environment where UB is not called out (C) then interventions to increase knowledge and improve awareness (R) can lead to an improved ability to recognise UB (M1) and can lead to reflection about past behaviour (M2), stimulating behaviour change away from UB (O1) likelihood of addressing UB in the moment (O2) and reducing likelihood of UB occurring in the future (O3)
Education, awareness and general group discussions	Training to increase knowledge of what UB look like, how to tackle / increase general awareness of it	[76, 86, 88, 89, 91–93, 95, 100, 106, 111, 116, 125, 137, 141, 156, 160, 164, 169, 174, 181]	
**12. Implementation-aiding strategies (managers/leaders)—Evaluated**			**CMOC 19. Implementation aiding strategies** When delivering a complex intervention to reduce UB (R) which requires sustaining over a long period (C), providing time and resource to implement momentum-building strategies can enable greater belief that the programme is an authentic effort to reduce UB, thereby increasing engagement (M1), increasing commitment to the intervention by key actors (M2) and increasing motivation for leaders and managers to implement further strategies to reduce UB (M3) which can increase effectiveness of other strategies to reduce UB (O)
Action planning or goal setting	Staff come together to plan other strategies to tackle UB. Can foster a sense of co-creation	[92, 93, 101, 106, 108, 111, 160, 169, 181]	
Building a repertoire of strategies	Enables flexible intervention delivery, with repertoire of activities to tackle UB enabling targeted responses to different scenarios	[132, 169]	
***13. Changing recruitment processes (all) – Unevaluated***			**CMOC 20. Changing recruitment processes** Implementing strategies to reduce UB e.g. novel selection methods (R) at the point of recruitment (C) can slowly change the perception of social norm towards civility (M) if the individuals behaving badly leave (C2) which can reduce likelihood of staff engaging in UB (O)
* Changing recruitment criteria (unevaluated)*	*Recruitment criteria to include personality / emotional intelligence tests to decrease recruitment of people who will not flourish in civil organisational culture*	[79, 98]	
* Dismissal (unevaluated)*	*Dismissing instigator known to have UB behaviour from employment*	[164]	

^a^These programme theories describing how strategies work present the resource (R) to make clearer the strategy being discussed within the CMOC. This method has been described by Dalkin et al. [33]. Later CMOCs e.g. to describe the key dynamics do not report the resource in this way

Some strategies were tested in the 42 interventions we outlined above, whereas others were not. Those that have been tested we refer to as ‘evaluated’. Strategies which have not yet been evaluated were reported in the 106 non-intervention reports we identified in the literature. These unevaluated strategies are presented in italics in Table 5.

As our analysis progressed, we identified that some strategies worked through shared underlying mechanisms. This enabled both creation of our categories of strategies as well as the shared programme theories for each category. The programme theories depicted in Table 5 set out how, and in which circumstances, use of various strategies are appropriate.

### Key dynamics impacting how and when interventions work

We identified twelve Key Dynamics which explored common issues, contradictions, tensions or considerations identified as important to intervention design. These can be common pitfalls which lead to unintended consequences, ways to improve effectiveness, and important design trade-offs. Programme theories are presented for each to highlight how and why these dynamics work; and these can have positive (O +) or negative (O −) outcomes. Helping to tackle some of these key dynamics are fifteen Implementation Principles which will be explored in the next section.

#### Key Dynamic 1. Interventions need to address systemic factors that contribute to UB not only individual factors

Organisations were found to largely assume that individual, rather than systemic factors, were driving UB [20]. A focus on individual factors leaves systemic contributors unaddressed and can lead to implementation of interventions which do not tackle the root causes of UB. Interventions focusing on individuals, such as boosting individual resilience, awareness, or ability to speak up can have their effectiveness undermined when systemic contributors, such as tackling workplace culture or design, remain unaddressed, and continue to contribute to UB occurring [124]

**Table:** 

**CMOC 21: Addressing systemic contributors**
If systemic issues such as understaffing, stress resulting from the way work is structured, and lack of resources are addressed at the same time as implementing an intervention (C), then interventions to address UB will have greater success (O +), because staff feel better-supported and psychological distress is reduced (M)

**Fig. 5:**
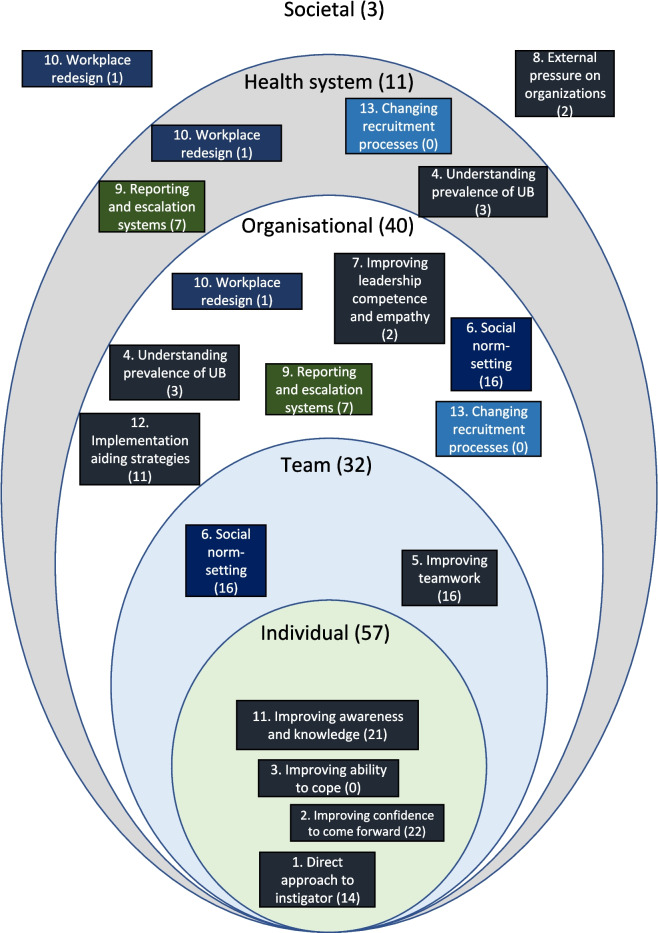
Interventions mapped according to their level of implementation. Numbers in brackets indicate the all the times strategies were evaluated within each category. Strategy categories that are mentioned more than once are reflected in different colours for ease of identification

To depict the preponderance by intervention designers on individual factors, we have presented the 13 main categories of strategy to address UB according to whether the strategies seek to address Individual, Team, Organisational, Health System, or Societal-level issues (Fig. 5). The number of times which strategies were evaluated is depicted in brackets in the figure for each strategy (e.g. social norm strategies were evaluated 16 times in total) as well overall according to the level (e.g. Individual or Team) that they targeted. Figure 5 demonstrates that most evaluated strategies targeted individuals (e.g. to raise their awareness of UB) (*n* = 57), with organisational level the second-most frequent (*n* = 40). This highlights the extensive application of interventions focused on individual factors.

#### Key Dynamic 2. Focusing on individual staff can have unintended consequences for psychological safety

When systems are implemented that seek to weed out ‘bad apples’, psychological safety is not improved, patient safety is unlikely to be positively impacted, and systemic issues (see Key Dynamic 1) remain unaddressed.

**Table:** 

**CMOC 22: Identifying bad apples** Top-down interventions focused on identifying problematic individuals (C) can lead to other/wider contributors of UB remaining unaddressed (O −) and have a negative effect on team cohesion (O2 −) because it can inhibit development of an open culture promoting psychological safety (M1) and increase retaliatory reporting (M2) VS.
**CMOC 23: Enhancing psychological safety** In an environment dominated by hierarchy and power dynamics, interventions which address systemic contributors to UB (e.g. by reorganising the workplace, increasing role clarity and improving worker decision-making) (C) can reduce UB more effectively (O +) because an open culture and psychological safety are fostered (M)

#### Key Dynamic 3. How and why an intervention is expected to work must be clear otherwise evaluations of interventions can be misleading

Existing studies have claimed success or failure based on intermediate outcomes such as ‘level of awareness’ of UB, or adjacent outcomes such as ‘assertiveness’. For example, four included interventions relying on reports of UB as their primary outcome measure were reported by their authors as being ‘unsuccessful’ due to an increase in reports of UB post-intervention when compared to controls [91, 93, 154, 162]. However, an increase in awareness and reports of UB should be considered a success from a behaviour change perspective. Use of logic models, unfortunately not presented by any included study, would help understand such relationships, and would be essential to improving fidelity of such evaluations and for getting closer to measuring actual improvements in UB.

**Table:** 

**CMOC 24. Need for comprehensive evaluation**
If those responsible for developing and implementing a UB intervention clearly map out how it could work, draw on theory and invest in sufficient evaluation (C), then how it impacts patient safety, staff psychological wellbeing and marginalised staff groups can be determined (O +), because greater information regarding success can be determined (M)

#### Key Dynamic 4. Maintaining a focus on why it is important to reduce UB (e.g. to improve patient safety) is key when designing an intervention to reduce UB

It is important to remember that the primary reason to reduce UB should be to improve staff wellbeing and improve patient safety and quality of care. Improving the ability to speak up *in the moment* can be essential to improving patient safety [10, 11]. Implementing a reporting system which enables speaking up online at a later time may have no impact on patient safety, unless other strategies are implemented which improve psychological safety *when it matters*.

**Table:** 

**CMOC 25. Maintaining a focus on distal outcomes such as patient safety is important when designing an intervention to reduce UB**
When interventions to reduce UB maintain a focus on improving patient safety (C), then the ability to challenge UB in the moment or speak up about medical mistakes is more likely to be improved (O +), because staff may feel more psychologically safe (M1), and a greater focus on patient safety may enhance engagement (M2) and improve culture change (M3)

#### Key Dynamic 5. Encouraging bystanders to intervene is important for culture change but can lead to moral injury

Encouraging bystanders to intervene sends signals that UB is unacceptable. However, creating an imperative to intervene can also lead to moral injury if staff do not subsequently intervene and feel guilty for not having done so. Further, intervening can place staff at risk of reprisal if performed in an unsafe organisational climate. Staff should be encouraged to intervene only when they feel safe and confident to do so.

**Table:** 

**CMOC 26. Encouraging bystander intervention successfully** Encouraging bystander intervention (C) can lead to UB being addressed in the moment (O +) and drive social norms to move towards civility (O2 +) because bystanders feel protected and able to act on their sense of moral duty to intervene (M2)
**CMOC 27. Encouraging bystander intervention may lead to moral injury or reprisal** Encouraging bystander intervention (C) can cause moral injury to the bystander if they do not feel confident intervening (O −) or can lead to reprisal if intervening when it was not safe to do so (O2 −) because they may feel like they have failed in their moral duty to intervene (M)

#### Key Dynamic 6. Identifying unintended consequences of anonymous reporting systems is essential

Systems that enable speaking up anonymously can enhance ability to speak up even when feeling psychologically unsafe. However, anonymity can also increase ease of subversion of these systems through behaviours such as scapegoating, e.g. by filing false reports. This can be avoided with triage systems or databases.

**Table:** 

**CMOC 28: Misuse** Enabling anonymous reporting of colleagues (C) can lead to an increase in UB in the form of undermining and scapegoating (O-) because informal alliances and individuals can co-opt the reporting system to target specific individuals with false reports (M) AND
**CMOC 29: Enabling speaking up** Enabling anonymous reporting of colleagues (C) can mean instigators are approached by messengers or line managers, directly reducing UB (O +) because recipients or witnesses of UB are able to speak up even when there are low levels of psychological safety (M)

#### Key Dynamic 7. Interventions must be perceived as authentic to foster trust in management

To assess whether it is worth trusting management to provide a safe working environment, healthcare staff will assess the authenticity of efforts that management make to reduce UB. If an intervention is not seen as authentic, staff may not take it seriously and will disengage. Authenticity can be lost if (1) managers are simultaneously engaging in negative behaviours and sending mixed signals, or (2) if the intervention itself is clearly inadequate for its intended purpose.

**Table:** 

**CMOC 30. Intervention perceived as authentic** When interventions are seen as authentic, and senior staff role model professional behaviour (C), then staff feel more able to buy into the intervention (O) because it is perceived as a legitimate attempt at reducing UB (M) VS.
**CMOC 31. Intervention perceived as inauthentic** If managers implement an intervention to address UB but continue to role-model or tolerate negative behaviours (C1) or the intervention content is perceived as unlikely to have any effect (C2) then staff will disengage from the intervention (O −) because staff received mixed signals about authenticity and may thus dismiss it as inauthentic (M)

#### Key Dynamic 8. One size does not fit all—tackling UB generally requires multiple and sustained interventions to address underlying contributors

Many interventions do not address systemic contributors; rather, they only seek to target one or two contributors (of many) for a limited length of time. However, the existence of this limited intervention may inhibit more comprehensive interventions from being developed and put in place because something is ‘already being done’ (although only partially) about the problem.

**Table:** 

**CMOC 32. Tackling UB requires multiple and sustained interventions**
If an intervention does not address all UB contributors (C) this can allow UB to continue to develop (O −) and inhibit trust in management (O2 −) because contributors remain unaddressed and more comprehensive interventions to reduce UB are ignored (M)

#### Key Dynamic 9. Addressing manager behaviour is essential for building trust in management.

To be seen as genuine and to have adequate reach, interventions need to include managers and senior employees at all levels. This is especially important for those organisations where managers have been seen to engage or tolerate UB themselves and where trust in management is low.

**Table:** 

**CMOC 33: Participation** If managers include themselves as a recipient or target of an intervention (C) this can show that UB is no longer tolerated (O +) and can build trust in management (O2 +) because it signals to other employees that the intervention is genuine (M1) and suggests there is a real cultural shift taking place (M2) VS.
**CMOC 34: No participation** If managers do *not* include themselves as a recipient or target of the intervention (C) this can allow UB to continue (O −) and reduces trust in management (O2 −) because it signals to other employees that the intervention is unfair and/or managers are not taking it seriously (M1) and suggests there is no real cultural shift taking place (M2)

#### Key Dynamic 10. Interventions that are both inclusive and equitable are critical to ensure effectiveness and sustainability and for addressing inequalities

Minoritised groups, women and staff with disabilities experience more UB in the workplace. Yet, these groups are rarely considered in existing interventions to tackle UB. We only identified one published intervention seeking to address racism [82], and none that even mentioned women or minoritised groups. This imbalance reduces equity and fairness and causes members of these groups to feel left behind. For example, the following excerpt from one UK-based study notes: “despite their selflessness and arduous work, Black African nurses face structural and institutionalised discrimination within the NHS. Employers must challenge the dominance and hegemony that exists within the NHS to ensure greater equality of all employees” [139]. Interventions could, and should, be more targeted and designed to specifically reduce UB for these groups.

While equity is essential to the success of interventions, it is also important to include as many people as possible in an intervention and not target one group over another. This is because targeting interventions at specific groups could alienate certain groups or imply they are ‘at fault’. Thus, it can be very difficult to design an intervention that simultaneously addresses the additional burden of UB experienced by minoritised groups and women, while also not singling out or denying opportunities to other staff groups.

**Table:** 

**CMOC 35: Equity**
When UB interventions cater to the specific needs of groups which experience systematic inequalities (C), then they will feel better supported in their workplace (O +), because they feel heard, seen and validated where previously they felt ignored (M) VS.
**CMOC 36: Inclusion**
If UB interventions seek to include all staff, including minoritized staff and women, and recognises differences in experiences such as higher rates of bullying directed at such groups (C), then inter-professional conflict may be reduced (O +), because staff feel included and their differences acknowledged (M)

#### Key Dynamic 11. There are trade-offs between fixed interventions and flexibility

Some interventions are inherently flexible and enable use of a repertoire of strategies that may be more effective in different contexts, such as CREW, increasing effectiveness. However, this can affect fidelity. This is because using different components when an intervention is delivered in different contexts makes it difficult to measure which mix of context and component was responsible for intervention success.

**Table:** 

**CMOC 37. Enhanced flexibility**
When implementing an intervention to address UB which draws on flexible implementation (C) this can enhance efficacy of the intervention to reduce UB (O +) because it may enable better adaptability of strategies to specific scenarios (M) AND
**CMOC 38. Reduced fidelity**
When implementing an intervention to address UB which draws on flexible implementation (C) this can reduce the ability to identify how to change the intervention to improve future efficacy (O +) because variability in implementation delivery across organisations and contexts can make it difficult to identify which components work (M)

#### Key Dynamic 12. There are trade-offs between a theory-first and practice-first intervention design

Many interventions are rooted in practice, or rather uncritically replicate existing interventions tried elsewhere. Few interventions were based on academic theory or contemporary behavioural science. A practice-led design may be rapid to design and implement and be more able to fit into existing organisational structures, but risks lacking articulation and understanding of how and why an intervention is supposed to (and did or did not) work. Simultaneously, a theory-led design can also risk being distant from what occurs in practice and being slower to roll out. As the study of such interventions progresses, provision of resources highlighting behavioural techniques for addressing UB for those embedded in practice may help bring these two approaches closer together.

**Table:** 

**CMOC 39. Theory-led**
If an intervention to reduce UB is being implemented while drawing on theories about how UB may arise (C) then an intervention may be slower to roll out (O1-) and more distant from ‘what occurs in practice’ (O2-) because it is facilitating a more robust evaluation process (M) and puts priority on theory over practical considerations (M2) VS.
**CMOC 40. Practice-led**
If an intervention to reduce UB is implemented rapidly with a practice-first mindset (C) then an understanding of its effectiveness may be compromised (O −) because the evaluation process may not have been adequately considered (M)

### Implementation principles to improve how interventions work

We identified fifteen further implementation principles that need to be considered by intervention designers to address the Key Dynamics outlined above. In summary, these principles include (1) ensuring organisational reach, (2) co-creation with staff, (3) assessing organisational landscape before implementation, (4) having dedicated staff to lead work to tackle UB, (5) ensuring skilled facilitation when using training, (6) drawing on multiple simultaneous strategies, (7) maximising visibility across the organisation, (8) intervening early where possible, (9) engaging managers and leaders, (10) ensuring the intervention is perceived as just and not punitive, (11) maximising existing organisational opportunities (e.g. appraisals), (12) managing organisational turnover and change to ensure programme continuity, (13) tackling instigators not victims, (14) incorporating ongoing evaluations and (15) not mixing hierarchies in group sessions. Additional File 5 highlights these principles in full detail, provides a programme theory underlying how these principles work, and maps these to the Key Dynamics. Each principle can help address one or more Dynamics.

## Discussion

Our review set out to investigate how and why interventions to address UB between staff in acute care work, and whom they benefit. We found that overall, interventions to reduce, mitigate and prevent UB are at an early stage of development and evaluation and their ability to impact the prevalence of UB is uncertain. While we identified 42 reports of interventions, most were small in scope, implemented in only one organisation, focused on individual-level contributors to UB, and only delivered to a subset of organisational staff. While UB is associated with reduced patient safety in both simulation and cross-sectional studies [1, 10, 11], no intervention measured changes in patient safety; however, some studies did measure changes in staff wellbeing (e.g. [156, 169]).

**Fig. 6:**
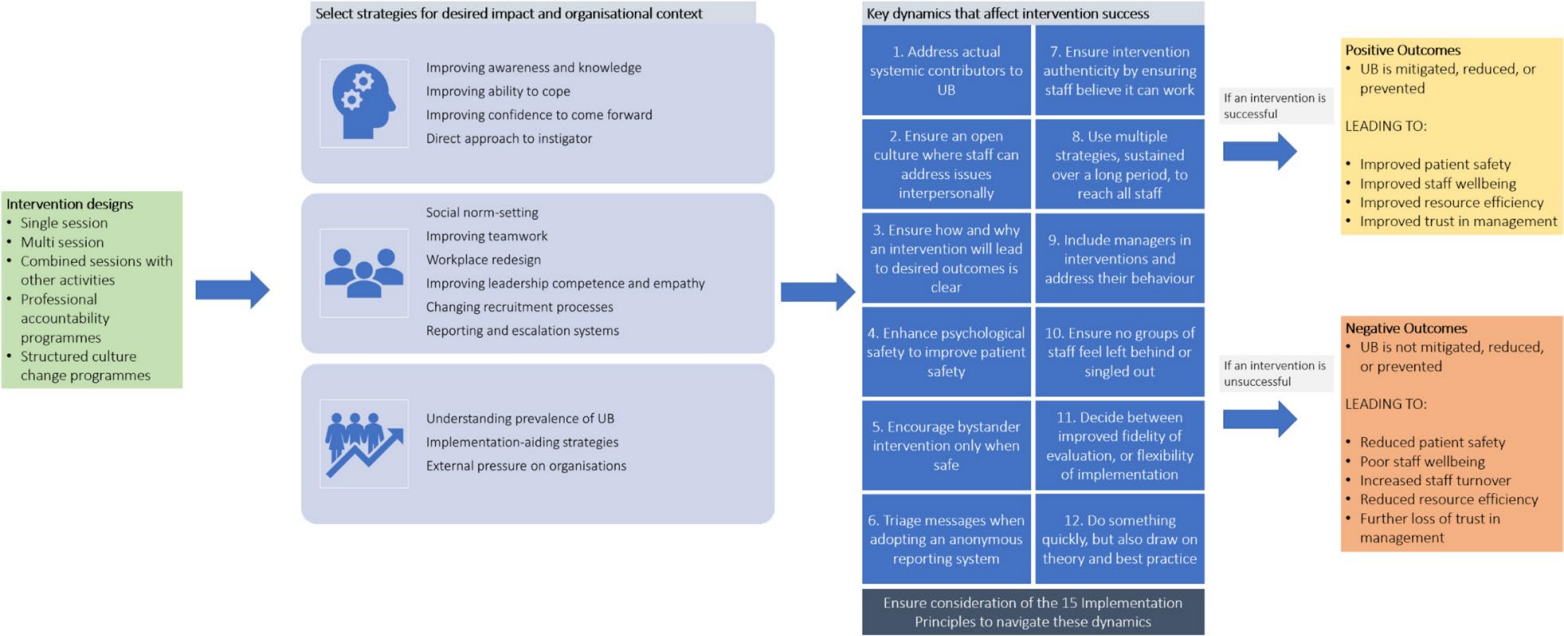
Final overall programme theory diagram

Our organisation of strategies according to their level of implementation, mechanism of action and the Key Dynamics, provides guidance on which strategies are appropriate in different circumstances. This is schematically represented in our overall programme theory diagram in Fig. 6. This overall programme theory broadly illustrates “how interventions can work to reduce, mitigate, or prevent UB, why, and under which circumstances”. It is important to note that this schematic only reflects strategies to address UB in acute care identified in this review.

We found that interventions in acute healthcare are preoccupied with individual behaviour, despite most contributors to UB being organisational and systemic [20]. Other reviews of interventions to reduce UB (including outside of healthcare) have highlighted this, noting that “the assumption that workplace mistreatment will be lessened if more people know about it, know how to recognise it and be more assertive in their responses to it (…)is a flawed assumption” [24]. An overly individual focus could lead to interventions being undermined by unaddressed systemic contributors (e.g. frustrating workplace designs, and a lack of job resources). An implication of this that future interventions should move towards addressing these systemic drivers as a priority [8].

Our work supports other authors who identified the risk that certain reporting systems can lead to a “worsening of safety culture by eroding trust and respect among healthcare professionals and teams, which affects both patient safety and individual well-being” [182]. Thus, maintaining a focus on *why* it is important for UB to be addressed is urgent and essential, to ensure interventions benefit staff and patients and to avoid perceptions of being ‘tick-box exercises’ [160]. This implies that interventions should focus on fostering a culture that supports building psychological safety, relationships between staff, and the ability to openly and freely talk to one another to manage conflicts before they escalate, to increase likelihood of success.

Overall, we found that theoretical bases of interventions and how and why they were intended to work were not well-reported. No included reports presented logic models nor drew on contemporary behavioural science that may lead to, or at the very least, facilitate long-term behaviour change. This finding suggests that authors may not always understand how intervention components will produce the desired effects. This may delay the advancement in rigorous research and understanding of UBs that is achieved by long-term repeated/iterative testing and developing of theories and logics. Other authors reviewing interventions in this area have suggested a lack of grounding in theory may be because “organizations are initiating their own research rather than turning to experts and academics to conduct analyses” [26], as highlighted in Key Dynamic 12. It is possible that provision of further guidance, such as in this review, will help others in practice to feel comfortable drawing on more theory-based interventions. Future reports of interventions should present logic models and make explicit any assumptions regarding how they intend to reduce UB with their chosen intervention design.

Key Dynamic 10 emphasises how existing research has been conducted with little regard to the additional burden of UB that women, staff from minority backgrounds, or with disabilities, experience. Only one intervention sought to address racist UB in acute care [82], and no others addressed this issue. Furthermore, sexual assault and sexism remain prevalent issues in the UK NHS and healthcare systems and societies worldwide, together with other known widespread issues such as racism, and disability and discrimination against LGBTQ + people. For example, over 4000 NHS staff between 2017 and 2022 were accused of rape, sexual assault, harassment, stalking or insults towards other staff or patients and only 576 have faced disciplinary action [183]. Similarly, in Australia, a survey of UB across seven hospitals found that 14.5% of staff had experienced “extreme” UB such as sexual assault, inappropriate touching and physical violence [1]. Intervention architects may assume that addressing UB in general may work to address issues such as misogyny, microaggressions and racism. We also found no mention of co-design of interventions with stakeholders by intervention architects, which may have resulted in interventions not targeting key outcomes relevant to the healthcare workforce. Our findings indicate the experiences of women and minoritised staff are unlikely to be addressed without specific effort. Further interventions must consider and addresses the inequitable impact of UB on female staff and staff from minoritised backgrounds as a core aspect of intervention design.

### Recommendations for future research

Interventions in this review had many limitations. Incorporating contemporary behavioural sciences theories which underpin long-term behaviour change into both the design and evaluation of interventions should be a priority. Relevant theories include the Capability, Opportunity, and Motivation for changing Behaviour (COM-B) approach [184], implementation science frameworks and theories (e.g. Consolidated Framework for Implementation Research (CFIR), Integrated Promoting Action on Research Implementation in Health Services (i-PARIHS) [185, 186], or Normalisation Process Theory (NPT). Future studies must fully clarify through logic models how and why intervention components are anticipated to lead to desired outcomes, including how implementation challenges in diverse contexts will be addressed. Future research may need to draw on multiple theories to explain how and why their intervention is intended to drive the desired effects.

Reports of evaluations of interventions should also give greater priority to reporting implementation context and how it could have impacted effectiveness. This will inform a greater understanding of why a particular strategy may work in one context but not another. Interventions must also address actual contributors to UB; however, to do so they need to first understand what they are. Few well-developed tools and instruments exist which determine contributors to UB; rather, the majority simply assess broad prevalence of UB [120]. Tools should be developed that provide greater insight into *what* is contributing to UB in an organisation and *where* it is taking place while allowing differing experiences of staff from different backgrounds to be understood. Based on the results of this research, we have developed guidance for addressing UB in healthcare organisations. This guide is available to download at: https://workforceresearchsurrey.health/. 

Additionally, there is a need for future interventions to incorporate economic evaluations and cost-effectiveness studies to determine whether the benefits outweigh the costs of implementation. Lastly, we identified that interventions have been predominantly implemented and evaluated in the USA, Canada and Australia. We suggest there is a need to commission and deliver evaluations of interventions in other countries and health systems suffering from the prevalence of UB, such as the UK.

### Strengths and limitations

This research had several strengths. The realist method, informed by the RAMESES standards [31], enabled us to present a coherent synthesis of a complex and disparate landscape of interventions. To achieve this, we included a significant number of reports (*n* = 148) for a realist review with strong international representation. The review searches are a strength; we drew on a range of published and grey literature reports, and searches were updated until December 2022. The majority of the literature reviewed was published after 2013 (e.g. 27 of 42 intervention studies), significantly advancing previous reviews (e.g. Illing et al. 2013 [22]). This study has also taken a wider view of UB between staff, expanding beyond bullying, which has been a focus of previous work.

The review had limitations. We did not include analysis of interventions to *improve* civility, but rather only to *reduce* incivility; therefore, we may have inadvertently excluded interventions capable of addressing UB. Despite seeking and including grey literature, we are also aware that there are unpublished practice-based interventions in use that are not captured by our review methods.

## Conclusions

UB is a pervasive issue which negatively impacts patient safety and erodes staff wellbeing. UB is yet to be sufficiently addressed by existing interventions, despite the urgent need to do so. Most intervention studies were conducted in the USA, Australia and Canada. The majority of these do not address systemic contributors to UB and rely on education or training workshops to boost individual knowledge and awareness of UB, improve ability for staff to speak up, or seek to identify problematic individuals. Such approaches *may* reduce prevalence of UB; however, it is currently unclear whether these interventions positively impact organisational culture, patient safety or staff psychological wellbeing. Interventions that focus on *both* individual and systemic contributors are required to effectively reduce UB. Issues such as lack of trust in management caused by pervasive, unaddressed UB presents a significant barrier to staff engagement with interventions. Fostering a culture that supports staff on the receiving end of UB to safely speak up can signal that UB is not tolerated. Future interventions would benefit from drawing on modern behavioural and implementation science principles, incorporating economic analyses, focusing on systemic issues that produce UB, and acknowledging and addressing the additional burden of UB experienced by women and minoritised staff.

### Supplementary Information

**Additional file 1.** Initial theories regarding strategies to reduce unprofessional behaviour.

**Additional file 2.** Search syntax and process.

**Additional file 3.** Table of intervention characteristics.

**Additional file 4.** Use of strategies in different intervention types.

**Additional file 5.** Implementation principles.

## Data Availability

The datasets used and/or analysed during the current study are available from the corresponding author on reasonable request.

## Abbreviations

BMA: British Medical Association
BME: Black, Minority and Ethnic
CFIR: Consolidated Framework for Implementation Research
CINAHL: Cumulative Index to Nursing and Allied Health Literature
CMOC: Context-Mechanism-Outcome Configuration
CREW: Civility, Respect and Engagement in the Workforce
HCPC: Health and Care Professions Council
HMIC: Health Management Information Consortium
i-PARIHS: Integrated Promoting Action on Research Implementation in Health Services
LGBTQ +: Lesbian, Gay, Bisexual, Transgender, Queer and others
MRT: Middle Range Theory
NHS: National Health Service
NICE: National Institute for Health and Care Excellence
NPT: Normalisation Process Theory
PRISMA: Preferred Reporting Items for Systematic reviews and Meta-Analyses
PROSPERO: International database of prospectively registered systematic reviews
RAMESES: Realist And Meta-narrative Evidence Syntheses: Evolving Standards
RCT: Randomised controlled trial
UB: Unprofessional behaviour
UK: United Kingdom
USA: United States of America
