# Supplementary material for: Interventions to address unprofessional behaviours between staff in acute care: what works for whom and why? A realist review
Source: BMC Med. 2023 Oct 31;21:403. doi: 10.1186/s12916-023-03102-3 (PMC10617100; doi:10.1186/s12916-023-03102-3)
Supplement: Supplementary file 1 — Additional file 1. Initial theories regarding strategies to reduce unprofessional behaviour. [file 12916_2023_3102_MOESM1_ESM.docx]

### Initial theories regarding strategies to reduce unprofessional behaviour

We also identified and coded excerpts relating to strategies in NVivo and initially classified them as either ‘preventative’ (i.e. intended to prevent UB) or ‘reactive’, i.e. try to mitigate the impact of UB, however, in the refined version we did not retain these categories as the distinction between these was not always clear. Our initial CMOCs are presented below.

Table 1. Initial theories of preventative strategies.

| **Strategy** | **Type of behaviours** | **CMOC** |
| --- | --- | --- |
| 1. Education, training of workforce about identifying unprofessional behaviours | Unprofessional behaviours (general) | When staff think unprofessional behaviours are necessary (e.g., bullying as part of training ‘to build resilience’) (C) then education regarding the effects of unprofessional behaviours may not lead to reflection on one’s behaviour (M - Instigator) therefore leading to no decrease in unprofessional behaviours (O) – 3  If the intervention is of too short duration I, then it is unlikely to reduce prevalence of unprofessional behaviours (O), because uptake of the intervention content will not be sufficient across the workforce (M) - 2  If the facilitators have the skills to properly implement the intervention(C), then unprofessional behaviours may become reduced (O), because they will improve collective awareness of UB (M1) and teach attendees how to identify UB (M2) - 1  If the education does not reach a critical mass of key actors in the workforce (e.g., management, bystanders, instigators) (C), then the intervention may not reduce unprofessional behaviours (O), because the intervention may not be taken up by those most proximal to the unprofessional behaviours (M) - 2  If the intervention does not have management support (C), then it is unlikely to be delivered for sufficiently long enough to achieve critical mass (M), because employee commitment will be lacking (O) - 2  If the intervention cannot be delivered in a flexible manner for employees around their other commitments (C) then it may not reduce unprofessional behaviours (O) as it is less likely to be taken up by people most proximal to the unprofessional behaviours (M) - 1  If the intervention is delivered to a high quality standard; i.e., is of sufficient duration (C1), is delivered flexibly around other commitment (C2), has support of management (C3), is delivered skilfully (C4) and reaches a critical mass of key actors (C5), then it may reduce unprofessional behaviours (O) because it can improve awareness of UB (M1), improve identification of UB (M2), enable instigators to reflect on their own behaviour (M3) and empowers victims and bystanders to come forward (M4) - 2  Piloting the educational/training materials with stakeholders (C) can lead to improved ability to reduce unprofessional behaviour (O) because it enables facilitators to improve intervention materials for greater efficacy (M) - 3 |
| 1. Teambuilding, Schwartz rounds | Unprofessional behaviours (general) | If the intervention is of too short a duration of implementation (C), then it is unlikely to reduce prevalence of unprofessional behaviours (O), because the workforce will not sense that they are supported by others (M1), and sense of collective empathy / mutual understanding has not been improved (M2) – 2  If the Schwartz rounds/teambuilding exercises are not delivered to a critical mass of the workforce (C) or the intervention does not have sufficient management support (C2), then it may not reduce unprofessional behaviours (O), as uptake will not be high enough to improve enough people’s collective sense of social support and empathy (M) – 2  If the intervention resource is of sufficient duration of implementation (C1) and is delivered to enough people (C2) then the intervention might reduce unprofessional behaviours (O), because it can improve sense of social support making people more likely to come forward to report unprofessional behaviours (M1), can improve sense of empathy which reduces the likelihood of unprofessional behaviours occurring (M2), and can improve empowered decision making processes in a team setting (M3) - 3 |
| 1. Coaching and mentoring | Unprofessional behaviours (general) | If the intervention is not visible or accessible to the workforce (C) then it may not reduce unprofessional behaviours (O), as workforce uptake will be low (M) - 2  If the mentor is skilled at mentorship (C1) and if enough of the workforce is mentored (C2) then mentorship may reduce unprofessional behaviours (O), as it would effectively improve perception of social support (M1), understanding of victimhood (M2), and improve victim self-confidence (M3) and individuals will feel in a stronger position to challenge negative behaviours (M4) - 2 |
| 1. Reverse mentoring (when lower-level employee mentors management) | Unprofessional behaviours (general), might work better on behaviours which require hierarchy | If managers have prior knowledge/training of the interventions to reduce unprofessional behaviours (C), then reverse mentoring may reduce unprofessional behaviours (O), as managers might gain knowledge, skills and motivation to tackle such behaviours (M) - 2  If managers do have knowledge and skills for how to effectively tackle unprofessional behaviours with follow-up interventions (C), then reverse mentoring may reduce unprofessional behaviours (O), as managers are more likely to accept there is a problem (M1), are more likely to better understand and have empathy for lower-level employees experiences (M2), and trust in management to tackle the problem will be increased (M3) - 3 |
| 1. Code of conduct (organisational) | Unprofessional behaviours (general) | If the code of conduct does not apply to all staff equally (C), then unprofessional behaviours may not be reduced (O), as instigators will not have their perception of risk increased (M1), and there will be no greater sense of empathy between staff members (M2)- 3  If the code of conduct does not have visible enforcement (C), then unprofessional behaviours may not be reduced (O), as there is no increase to perceived risk for instigators (M) - 3  If the code of conduct does reward positive behaviours (C), then unprofessional behaviours may be reduced (O), as there may be a greater sense of empathy between staff (M1), and perception of reward for instigators is reduced (M2) - 3  If the code of conduct is not visible and known to all employees (C), then it will not reduce unprofessional behaviours (O), as it will not increase awareness (or knowledge about how to tackle?) of unprofessional behaviours (M1), not increase perception of risk/reward for instigators (M2) nor improve sense of collegiality (M3) - 3  If employees sign a pledge to abide by the code of conduct (C1), then the code of conduct may further reduce unprofessional behaviours (O), because it will increase commitment to the code of conduct (M1), further increase awareness of UB (M2) and increase risk for an instigator (M3) - 3 |
| 1. Conflict management training | Bullying, harassment, undermining (targeted behaviours) | If there is a lack of follow up (C1) or short period in which the intervention is offered (C2) then conflict management training might not reduce unprofessional behaviours (O), because collective uptake is not sufficient in the population (M) - 2  If management does not support the intervention (C), then unprofessional behaviours may not be reduced (O), as the workforce would not be committed to the training (M) - 3  If the workforce does not know that they can access and take part in the training (C), then unprofessional behaviours may not be reduced (O), as uptake would not be sufficient in the population (M) - 3  If the organisation has a high prevalence of targeted behaviours such as harassment and bullying (C), then conflict management training might reduce conflict (O), because it may improve sense of collegiality and empathy (M) - 2  If conflict management training is skilfully delivered (e.g. with use of active learning techniques, reframing, role-play, or ‘I messages’) (C), then conflict management training may reduce unprofessional behaviours (O), because the intervention increases awareness of unprofessional behaviours (M1), provides an opportunity to practice new skills (M2; reduces escalation & reciprocity in situations involving conflict (M3), and improves sense of collegiality and empathy (M4) - 3 |
| 1. Environmental modification (cues) | Unprofessional behaviours (general) | If there are environmental prompts to remind staff of unprofessional behaviours (C) then it may reduce unprofessional behaviours (O) because there will be an increased awareness of unprofessional behaviours (M) and there will be an increased perception of risk to engage in UB (M2 – Instigators) - 2 |
| 1. Flattening hierarchies & training managers | Targeted behaviours that rely on hierarchy | If time and resources are not provided for the training (C), then unprofessional behaviours may not be reduced (O), as collective uptake of the intervention may not be sufficient (M) - 3  If facilitators do not have the requisite skills and knowledge to implement the intervention (C) then unprofessional behaviours may not be reduced (O) because managers will not uptake the required skills properly (M) - 2  If the training does not properly target a critical mass of managers (C) then unprofessional behaviours may not be reduced (O), as the perception of power gradient for victims will not be changed (M) - 3  If time and resources are provided for the training, facilitators have requisite skills to deliver the training, and training is delivered to a critical mass of managers (C1,2,3) then unprofessional behaviours may be reduced (O), as the perception of the power gradient may be changed for victims/bystanders (M1) leading to an increase in psychological safety (M2), there may be a reduced perception of reward for instigators (M3), managers may have a greater sense of empathy for those working under them (M4), and there may be improved team communication (M5) - 2  If there is a prevalence of targeted unprofessional behaviours that rely on hierarchy (e.g., bullying) in the organisation (C), then training to reduce hierarchy may be more effective (O), as it more explicitly targets the causes of these behaviours (M) - 2 |
| 1. Improving inclusion (e.g., staff networks, improving representation, unconscious bias training, allyship) | Racism, sexism, behaviours targeted at minority members of workforce | If the intervention is not sustained continuously (C), then unprofessional behaviours against minorities may not be reduced (O), because the intervention would not have reached enough people (low uptake) (M) - 3  Having allies who are not from ethnic minority backgrounds championing an intervention to improve inclusion (C) can improve efficacy of the intervention (O) because it can shine a light on assumptions underpinning structural biases by the challenge coming from less expected sources (M) - 3  If there are not multiple implementers to champion the intervention(s) (C1) or the champions do not have sufficient resource to maintain resilience (C2) then they may not effectively reduce unprofessional behaviours towards minorities (O) because there may not be sufficient uptake of the intervention (M) - 2  If the facilitators are not sufficiently skilled in the delivery of the training (C) then unprofessional behaviours towards minority members may not be reduced (O), because there might be improper uptake of the training by participants (M) - 2  If improvements are not able to be measured in response to these interventions (C), then unprofessional behaviours may not be reduced (O), because people will lose commitment to the intervention because they do not see change (M) - 2  If some staff who are not minority members are excluded from the interventions (C) then conflict may occur (O), as resentment could be increased in those who are excluded (M) – 3  If the intervention does not reach a critical mass of the workforce (C), then unprofessional behaviours towards minorities may not be reduced (O), because uptake will not be sufficiently high (M) – 2  If you see people of a similar background to yourself represented in senior management in the organisation (C) you are more likely to be committed to your organisation (O) because you will feel more empowered by working there (M) – 2  If the intervention has peer support (C1) and reaches a critical mass of the workforce (C2) then interventions focused on inclusion may reduce unprofessional behaviours towards minorities (O), as they can improve sense of fairness (M1), thereby reducing stress in victims/bystanders (M2), they can reduce the sense of a power gradient (M3), improve empathy towards colleagues from different backgrounds (M4), improve communication within teams (M5), help instigators understand differences in perception of their actions (M6), and provide positive role models for minority members (M–) - 2 |
| 1. Monitoring prevalence of unprofessional behaviours | Unprofessional behaviours (general) | If the monitoring of unprofessional behaviours is continuous (C) then it may reduce the impact of unprofessional behaviours (O), as it enables other strategies/interventions to be put in place to deal with UB earlier (M) - 3  If staff are made aware of the results of the monitoring (I.e., the prevalence) (C) then unprofessional behaviours may be reduced (O) because staff will gain trust in management (M1)  If actions are not taken when data about high prevalence of UB is made available (C) then unprofessional behaviours can be increased (O) because staff will lose morale (M1) and trust in management (M2) - 3  If monitoring is continuous (C1), unprofessional behaviours are seen as a problem (C2), and staff are more widely made aware of the prevalence of UB (C3), then unprofessional behaviours may be reduced (O), because there will be an enhanced awareness of unprofessional behaviours that can lead to further action (M1), it can improve collective trust in management to reduce these behaviours (M2) and knowledge that UB is being monitored can lead to improved psychological safety (M3) - 2 |
| 1. Positive role modelling/championing | Unprofessional behaviours (general) | If there are positive role models in place (C1) then unprofessional behaviours may be reduced (O), because victims and instigators can learn how to behave in a positive manner through social learning (M1) thereby collectively changing social norms/culture (M2) - 2 |
| 1. Visible commitment to reducing unprofessional behaviours by managers and gaining of trust from employees Commitment must be followed up by action or risk large decrease in trust. | Unprofessional behaviours (general) – strategy might be less effective with bullying or harassment | If managers visibly commit to reducing unprofessional behaviours in front of the workforce (C), then unprofessional behaviours may be reduced (O), because victims and bystanders may perceive that management is serious (M1), leading to a greater willingness to come forward (M2). Additionally, instigators may perceive there to be a greater risk to engaging in unprofessional behaviours (M3), and managers may be more likely to acquire more skills to deal with unprofessional behaviours (M4). - 3  If the commitment is not followed up by action by managers (C), then unprofessional behaviours may worsen (O), because staff will lose significant trust in management (M) - 3 |
| 1. Encouraging bystander intervention (once environment is relatively free of risk of reprisal) | Unprofessional behaviours (general) | If a bystander speaks up in a situation when they are lower in a power hierarchy than a bystander (C1), or where bullying is prevalent (C2), then they may face reprisal in the form of more unprofessional behaviours (O), because the instigator may perceive this action as an attack (M1) - 3  If bystanders are in a situation without power hierarchy (C1), then being encouraged to speak up may help reduce unprofessional behaviours (O), because it may identify instigators as unprofessional both to themselves and others (M1), leading instigators to reflect upon their actions (M2). Additionally, training may improve self-confidence of bystanders (M3), improve the bystanders’ abilities to correctly identify unprofessional behaviours (M4) can change social norms in the organisation to make UB less acceptable (M5) and can provide a sense of personal responsibility to employees to reduce and call out UB (M6)– 2 |
| 1. More stringent staff selection processes | Unprofessional behaviours (general) | If there is long-term commitment to changing staff selection processes (C) that results in a change in critical mass of the type of staff working in the organisation with positive values than mitigate against UB) (R), then more stringent staff selection processes may reduce UB (O), as it will reduce perceived exposure to UB (M1), thereby reducing prevalence of negative role models and reducing social learning of negative behaviours (M2) and desire to reciprocate (M3). Additionally, it may increase the perception of fairness in the workplace (M4). - 1 |
| 1. Improvements to work design | Unprofessional behaviours (general)  Might be useful with hierarchy where anonymous feedback is provided regarding manager behaviour | If work processes can be changed to reduce job demands (C), then conflict between staff may be reduced (O), because staff may have reduced stress (M) - 1 |
| 1. Multisource feedback | Bullying/harassment (targeted behaviours) | If 360 degree appraisal raters are able to provide anonymous feedback regarding the behaviour of a specific person (R), then, where there is a hierarchy in place (C), it may be particularly effective in tackling unprofessional behaviours (O) because it can help identify UB and who instigators are (M1) with low risk of reprisal to the reporter (M2). - 2  Additionally, existence of such an intervention (C) may increase collective perception that management is serious about addressing behaviours (O) because it requires managers to be put at risk of appraisal themselves. (M) - 2 |
| 1. Training for workforce (assertiveness, resilience, cognitive rehearsal) | More targeted behaviours but also improving ability to speak up | If a critical mass of employees is not trained (C), then these types of training intervention may not be successful at reducing unprofessional behaviours (O), because collective uptake is not sufficient (M) - 2  If the intervention is not visible and accessible (C), then these types of training intervention may not be successful at reducing unprofessional behaviours (O), because the training does not reach a critical mass of staff (M) - 2  If such interventions are not delivered competently by a trained facilitator (C), then unprofessional behaviours may not be reduced (O), because recipients will not have learned greater assertiveness (M) - 2  If a critical mass of employees is trained (C1), and the training is delivered competently (C2), then unprofessional behaviours may be reduced (O), because victims/bystanders may have improved confidence leading to an improved sense of psychological safety (M1), an increased understanding of their victimhood (M2), a reduced desire to escalate or respond (breaking the cycle) (M3), and improved teamworking/communication (M4) - 2  If delivered in a context of prevalent targeted forms of unprofessional behaviour (C), then these interventions may be particularly effective (O), because they can enhance resilience of victims (M1) and improve likelihood of bystanders to speak up (M2) - 2 |

Reactive strategies

Table 2. Initial theories of reactive strategies.

| Intervention | Type of behaviours | CMOCs |
| --- | --- | --- |
| 1. Acknowledgement of UB problem by management to the workforce | Unprofessional behaviours (general) | If staff think unprofessional behaviours are necessary to be able to do their jobs (C) (I.e., when training trainees), then acknowledgement by managers alone will not reduce unprofessional behaviours without further follow-up action (O), because there will be no commitment by employees to any strategies to reduce UB (M) - 2  If managers have the skills, knowledge and capability to follow up on the acknowledgement that there is a problem (C), then psychological safety of employees may be improved (O), because there can be an increased perception of risk to the instigator (M1), improved trust in management by victims/bystanders (M2), and improved perception of social support and self efficacy (M3) - 2  Acknowledgement of the problem by management (C) may lead to identification and revealing of instigators by the workforce (M) which can lead to their dismissal (O1), lowering prevalence of unprofessional behaviours (O2) - 2 |
| 1. Changing language around reporting incidents | Reduce potential for backlash in situation with hierarchy | If there are situations with a strict hierarchy, such as with surgeons and their trainees (C), then changing language to neutral non-blaming language when reporting errors can lower incidence of unprofessional behaviours (O), as it reduces the desire for senior staff to escalate or respond to critiques, which would generate conflict (M) - 3 |
| 1. Being inspected by regulatory body | Likely to only detect unprofessional behaviours that are having a more severe impact on care quality (e.g., patterns of bullying) | If actions are taken by management in response (C) to inspections by a regulatory body which find a high prevalence of UB (R), then this may lead to a reduction in unprofessional behaviours (O), because it can increase awareness of unprofessional behaviours in the organisation (M1), leading to acceptance that there is a problem by managers (M2) - 1 |
| 1. Dismissal of offending staff | Unprofessional behaviours (general) | If instigators are dismissed and replaced with more appropriately behaving staff (C), then unprofessional behaviours may be reduced (O), because there will be a greater sense of risk to other instigators (M1) reduced frequency of negative role modelling in the workplace (M2), and a perception that management is serious about addressing these negative behaviours and that there are consequences for perpetrators thus encouraging others to report (M3) - 2 |
| 1. Warning offending staff, removing key organisational roles from them | Unprofessional behaviours (general) | If removing perpetrators from their roles is considered by instigators to be a sufficient punishment (C), then warning offending staff and removing their roles may reduce unprofessional behaviours (O), because it will increase their perception of risk and serious consequences if they continue negative behaviour (M1), and may cause them to reflect on their actions (M2) - 1  If removing roles from an instigator is implemented (C) then an instigator may have an increased perception of risk when engaging in UB (O) because they will have been identified as an instigator to others (M) - 1 |
| 1. Educating instigators about their impact | Unprofessional behaviours (general) | Educating instigators about the impact of their actions (C) may reduce the chance of them engaging in further UB (O), because it may cause reflection upon their actions (M1), help them see the perspective of others (empathy / being in other’s shoes) (M2) and may increase their sense of risk to engage in such behaviours (M3) - 2 |
| 1. Individual – gathering evidence about unprofessional behaviours (e.g., diaries, emails) | Likely to work on targeted behaviours | If an individual is planning to report another for targeted forms of unprofessional behaviour towards them or others (C), then collating evidence of these unprofessional behaviours can increase effectiveness of reporting to reduce these behaviours (R), because it can improve confidence and self-efficacy when coming forward (M1) and increase how seriously management takes such claims (M2) - 2 |
| 1. Mediation | Likely to work only with targeted behaviours where there is no hierarchy (e.g., rudeness) | If there is a power gradient between two individuals in mediation (C), then mediation can increase unprofessional behaviours (O), because it gives the instigator further opportunity to engage in such behaviour (M1) and does not reduce the underlying perception of risk to engage in UB (M2) - 3  If in a case of unprofessional behaviours between two or more individuals with little hierarchy (C1), and a skilled mediator (C2), then mediation may be effective to reduce unprofessional behaviours (O), because it can cause an understanding in differences in perception between the victim and instigator (M1) which can lead to improved mutual understanding and empathy for each other (M2) – 3  If mediation is not known about, visible or trusted by employees (C) then participants may not engage with mediation (M) which will not reduce UB (O) - 3  Use of mediators external to an organisation (C) may be less effective at reducing UB than using internal ones (O) because the targets of mediators may perceive them as less familiar with their situation (M) - 1 |
| 1. Rewarding whistle blowers, presence of ‘speak up guardians’ etc. | More pervasive cultures of unprofessional behaviours | If there is an onus on the victim to report unprofessional behaviours (C), then unprofessional behaviours may not be reduced (O), as there can be significant fear of reprisal from the victim (M1) - 2  If whistle-blowers are rewarded in a manner visible to other employees (C) then this can increase speaking up about UB (O1) and thereby reduce likelihood of unprofessional behaviours (O2), as it can improve perception of social support for victims/bystanders (M1) and improve self-confidence/self efficacy (M2) thereby increasing sense of psychological safety (M3). Additionally, it can improve trust in management that they will take any claims seriously (M4). - 2 |
| 1. Creating ‘safe space’ and opportunities to speak up ***anonymously*** | More effective in situations where there is risk of reprisal (e.g. very prevalent negative cultures, much hierarchy) | If there are opportunities for victims or bystanders to speak up completely anonymously (C) then this can reduce unprofessional behaviours (O) because instigators might have a greater sense of risk from knowing they can be anonymously reported (M1) Additionally, the reduced chance for identification significantly lowers fear of reprisal for the reporter and massively increases sense of psychological safety when doing so (M2) - 2 |
| 1. Increasing safe space to speak up (***non-anonymously***) e.g., ensuring clear rules are in place and alleviating concerns that individuals may have regarding repercussions, etc. | Unprofessional behaviours (general) | If there are significant power differentials in place in the organisation (C1) or high prevalence of UB (C2), then opportunities to speak up non-anonymously can be ineffective (O), because victims and bystanders feel a lack of psychological safety (M) - 2  If there are clear and trusted routes for reporting unprofessional behaviours in an organisation (C), then unprofessional behaviours may be reduced (O), because it can lower perception of risk when speaking up for victims and bystanders (M1), and improve confidence/self-efficacy (M2) - 2 |
| 1. Removing victims from problematic environment | Bullying, harassment, undermining – targeted behaviours | If victims are removed from a problematic environment (R) but the instigators are not removed, or not punished in some way (C1), this may not be effective in reducing unprofessional behaviours towards others (O), because there will be no consequences observed by others for the instigators who may feel free to continue engaging in UB (less sense of risk for the instigators) (M1) and no change in social norms (M2) - 2  If in an environment rife with targeted UB such as bullying and harassment (C), removing victims from the reach of the instigator within an organisation may reduce their experience of the unprofessional behaviours (O), because they will have less perceived exposure to the instigator (M) - 3 |
| 1. Seeking help at a professional body | Targeted unprofessional behaviours | If the intervention is visible and accessible to victims/bystanders (C1), then seeking help at a professional body (R) can help reduce the impact of unprofessional behaviours (O), because it improves self-efficacy to come forward for victims (M1) and improves sense of social support (M2), thus increasing psychological safety and resilience (M3) – 2  If help is sought at a professional body (R) at the first sign of being exposed to targeted and frequent forms of UB (C) then the impact of UB on the victim may be reduced (O) because the victim may have increased sense of social support and improved ability to cope (M1) – 2  If help is sought at HR of an organisation before seeking help at a professional body (C) then it may avoid further backlash on the reporter (O) because others will perceive them as having done what they could before involving external actors (M) |
| 1. Therapeutic/coping strategies | Targeted unprofessional behaviours | If the intervention is visible and accessible (C1), and has sufficient follow-up and duration (C2), then therapeutic strategies may work to reduce targeted unprofessional behaviours (O) because they can increase understanding of victimhood by victims/bystanders to increase sense of empowerment (M1), increase sense of social support (M2) and thus sense of psychological safety (M3), and reduce stress (M4) - 2 |
