# Supplementary material for: Interventions to address unprofessional behaviours between staff in acute care: what works for whom and why? A realist review
Source: BMC Med. 2023 Oct 31;21:403. doi: 10.1186/s12916-023-03102-3 (PMC10617100; doi:10.1186/s12916-023-03102-3)
Supplement: Supplementary file 2 — Additional file 2. Search syntax and process. [file 12916_2023_3102_MOESM2_ESM.docx]

# Additional File 2. Search syntax and process.

This Additional File outlines our full search process and full list of search strategies used throughout the review across multiple databases and timepoints. These are outlined below.

## Full description of search process

## Results of searching and screening

The following results are in chronological order and reflect multiple cycles of searches, screening, and relevancy/rigour assessments. The PRISMA diagram in the manuscript, however, displays the process of searching, screening and inclusion in a non-chronological order, i.e., the update search results are incorporated with the initial search results in the diagram. This demonstrates more clearly how many studies were found, included, and excluded, at Step 1 vs. Step 2 of the review.

### Step 1 (November 2021): Identifying existing theories and scoping the literature

We included 38 documents in the initial theory generation step, comprising 30 identified from the proposal, five from informal searches, and three from the team.

### Step 2 (November 2021 – February 2022): Searching for evidence

The initial systematic search identified 2,629 records after 99 Google Scholar search results after cross-database deduplication. Initial independent pilot screening of 54 (of 2,629) records resulted in 16 papers included for full text screening with 100% agreement. Independent screening by two reviewers (JA and RA) of 267 (10% of 2,629) records led to 72 being included for the next step. Disagreement occurred on 40 items (15%) and was resolved through discussion between JA and RA. The remaining 2,308 papers were screened by JA against the inclusion/exclusion criteria. Of these, 400 papers were selected for full text screening.

#### Google and Google Scholar Searches (November 2021)

Two searches were performed on Google Scholar via use of Harzing’s Publish or Perish software (each was limited to the 50 most relevant results). This was to ensure we captured relevant grey or academic literature identified by Google’s algorithms. Search strategies are presented in Appendix 3. After duplicate removal, 99 papers remained for title and abstract screening. Papers were excluded largely because they were not related to unprofessional behaviours between staff. Sixty-three papers were selected for full text screening and then combined with the systematic search results at the full text screening stage. An additional 52 sources were identified through searches on Google (30) in 2021, from the project team (12) and from stakeholders (10) during 2022 (see Additional searching below). In total, 603 papers were selected for full-text screening. After cross-deduplication of these various sources of literature, 537 full-text papers were eligible for screening.

#### Step 2: Full text screening

Following application of major/minor screening criteria to the 537 potentially relevant papers, 193 papers were determined to have major relevancy, and 352 were excluded for having minor relevancy. In addition, 34 (10%) of those papers excluded at this stage were selected for independent screening (JA and RA). From these, two decisions were found to be in conflict. These two discrepancies were resolved through discussion and remained excluded. The 193 papers were then screened against inclusion criteria and conceptual richness and 148 papers were included.

#### Relevance and rigour

The remaining 148 papers were screened for relevancy according to the realist method, meaning papers had to include passages suitable for theory gleaning, testing, or refining with respect to either causes or strategies^47^. Studies which lacked such passages were therefore screened out (n=45) resulting in 103 papers. An additional 6 studies from the team and 2 studies from citation tracking key intervention papers were added at this stage, thus, 111 documents were included for conceptual thickness screening.

#### Conceptual thickness screening

As a result of conceptual thickness screening as outlined in Table 2 above, 47 sources were excluded at this stage for lacking conceptual thickness. This meant that 64 rich sources were included at this stage.

### Additional searching and evidence gathering (August – November 2022)

#### Search update to expand relevancy criteria to USA intervention studies (August 2022)

The team decided to include USA-based literature because earlier searches had not identified any UK interventions. We reran the same searches for ‘step 2’ but limited result to USA (excluding HMIC due to its predominance of UK content, and NHS Evidence which was withdrawn in April 2022). This identified 1,298 records, which reduced to 57 records once duplicates and previously screened records were removed. These 57 records were screened but none were included because no interventions were identified. However, we did re-include 10 USA studies from our step 2 search which had previously been excluded because of country.

A further 9 relevant studies were identified by citation searching (forwards and backwards) from nine key further USA studies using the CitationChaser Shiny App^48^. Four studies were identified by the team. Reference scanning of the 10 included USA studies and the 4 identified by the team found a further 11 and 2, resulting in a total of 36 additional papers from August 2022 searches. A total of 138 papers were included (step 1 and 2) prior to final update searches.

#### Searches for behavioural and organisational psychology theories (November 2022)

Members of the advisory group indicated that literature from behavioural or organisational psychology may provide useful evidence, so we searched for anti-bullying interventions that use behavioural science theory in: ABI/INFORM Collection (ProQuest), Business Source Premier (Ebscohost) and Google Scholar. The lack of relevant search results prompted a further search (in ABI/INFORM Collection (ProQuest) and Google Scholar). Due to low relevancy of results during screening, papers found in this search were not utilised.

### Search update (December 2022)

The above searches were repeated on 9^th^ December 2022 (except for NICE Evidence (withdrawn in April 2022)) to ensure our review was as up to date. The search strategies were reviewed before running the final update searches and no changes made. This identified 3890 records, which reduced to 192 records when we removed duplicates and previously identified records. After title and abstract screening, 36 papers were retained for full text screening. Included from this update were 8 papers and an additional 2 studies from the team (10 total).

### Total included literature (March 2023)

148 total papers were included. This included 38 papers from step 1; 100 papers from step 2; and 10 from our updated December 2022 search.

## List of search syntaxes

1. Google Scholar search 2021
2. Google Search 2021
3. Systematic search (multiple sources) 15-02-2022
4. USA update search (multiple sources) 25-08-2022
5. CitationChaser search 25-08-2022
6. Update Systematic Search (multiple sources) 09-12-2022

#### 1. Google Scholar (Via Harzing’s Publish or Perish)

Google Scholar (Via Harzing’s Publish or Perish)

Date searched: 19-11-2021

Records downloaded: 100

Search 1. 446 records found, first 50 downloaded (sorted by relevance)

bullying | harassment | discrimination|unprofessional AND workplace|worker|staff AND model|framework|concept|idea|opinion|theory|view|perception|attitude|theories [Searched in Publish or Perish Title Field]

Search 2. ~1,120,000 records found, first 50 downloaded (sorted by relevance)

interventions|strategies|techniques|program|programs|programme|programmes AND unprofessional|bullying|harassment|discrimination AND nurse|doctor|paramedic|hospital|ambulance|staff|professional AND emergency|acute|trauma [searched in Publish or Perish Keywords field]

#### Google

Date searched: 23-11-2021

Records browsed: First 5 pages

allintext: interventions|strategies|strategy|techniques|program|programs|programme|programmes unprofessional|bullying|harassment|discrimination nurse|doctor|paramedic|hospital|ambulance|staff|professionals emergency|acute

#### 2. Systematic search 15-02-2022

Sources searched:

- CINAHL (EBSCOhost)
- Embase Classic+Embase (Ovid) 1947 to 2022 February 11
- Ovid MEDLINE(R) ALL 1946 to February 11, 2022
- HMIC Health Management Information Consortium 1979 to November 2021
- NICE Evidence Search https://www.evidence.nhs.uk/
- Patient Safety Network <https://psnet.ahrq.gov/>

CINAHL (EBSCOhost)

Date searched: 14-02-2022

Records found: 1279

# Query Results

S37 S35 NOT S36 1,279

S36 TX ( ("elder mistreat*" or "elder abuse*" or "elder neglect*") ) 4,823

S35 S33 NOT S34 1,322

S34 ( ( (MH "Child") OR (MH "Adolescence+") OR (MH "Minors (Legal)") ) NOT (MH "Adult+") ) 469,789

S33 S22 NOT S32 1,391

S32 S28 NOT S31 365,968

S31 S29 OR S30 1,683,919

S30 (MH "Australia+") OR (MH "Europe") OR (MH "Austria") OR (MH "Baltic States+") OR (MH "Belgium") OR (MH "Canada+") OR (MH "Chile") OR (MH "Colombia") OR (MH "Costa Rica") OR (MH "Czech Republic") OR (MH "Scandinavia+") OR (MH "France") OR (MH "Germany+") OR (MH "Greece") OR (MH "Hungary") OR (MH "Iceland") OR (MH "Ireland") OR (MH "Italy") OR (MH "Israel") OR (MH "Japan") OR (MH "South Korea") OR (MH "Luxembourg") OR (MH "Mexico") OR (MH "Netherlands") OR (MH "New Zealand") OR (MH "North America") OR (MH "Poland") OR (MH "Portugal") OR (MH "Slovakia") OR (MH "Slovenia") OR (MH "Spain") OR (MH "Switzerland") OR (MH "Turkey") OR (MH "United Kingdom+") OR (MH "United States+") 1,677,371

S29 (MH "Developed Countries") or (MH "European Union") or (MH "Organisation for Economic Co-Operation and Development") 10,234

S28 (S23 OR S24 OR S25 OR S26 OR S27) 404,118

S27 (MH "Africa+") 92,227

S26 (MH "Asia, Southeastern+") OR (MH "China+") OR (MH "Hong Kong") OR (MH "Macao") OR (MH "Mongolia") OR (MH "North Korea") OR (MH "Taiwan") OR (MH "Atlantic Islands") OR (MH "Indian Ocean Islands+") OR (MH "Melanesia+") OR (MH "Micronesia+") OR (MH "Polynesia+") 127,545

S25 (MH "Bangladesh") OR (MH "Bhutan") OR (MH "India") OR (MH "Yemen") OR (MH "United Arab Emirates") OR (MH "Syria") OR (MH "Saudi Arabia") OR (MH "Qatar") OR (MH "Oman") OR (MH "Lebanon") OR (MH "Kuwait") OR (MH "Jordan") OR (MH "Iraq") OR (MH "Iran") OR (MH "Bahrain") OR (MH "Afghanistan") OR (MH "Nepal") OR (MH "Pakistan") OR (MH "Sri Lanka") OR (MH "Asia, Central+") 108,764

S24 (MH "Argentina") OR (MH "Bolivia") OR (MH "Brazil") OR (MH "Ecuador") OR (MH "French Guiana") OR (MH "Guyana") OR (MH "Paraguay") OR (MH "Peru") OR (MH "Suriname") OR (MH "Uruguay") OR (MH "Venezuela") OR (MH "Belize") OR (MH "El Salvador") OR (MH "Guatemala") OR (MH "Honduras") OR (MH "Nicaragua") OR (MH "Panama+") or (MH "West Indies+") 72,856

S23 (MH "Albania") OR (MH "Andorra") OR (MH "Armenia") OR (MH "Azerbaijan") or (MH "Byelarus") OR (MH "Bosnia-Herzegovina") OR (MH "Croatia") OR (MH "Bulgaria") OR (MH "Georgia (Republic)") OR (MH "Gibraltar") OR (MH "Liechtenstein") OR (MH "Macedonia (Republic)") OR (MH "Moldova") OR (MH "Monaco") OR (MH "Romania") OR (MH "Russia") OR (MH "San Marino") OR (MH "Serbia") OR (MH "Ukraine") OR (MH "Yugoslavia") 11,801

S22 S5 AND S21 1,505

S21 S16 OR S17 OR S18 OR S19 OR S20 23,637

S20 TI ( (staff or employee* or work* or nurs* or doctor#) n5 (sexism or sexist or ableis* or racism or racist or (racial n3 abus*)) ) OR AB ( (staff or employee* or work* or nurs* or doctor#) n5 (sexism or sexist or ableis* or racism or racist or (racial n3 abus*)) ) OR SU ( (staff or employee* or work* or nurs* or doctor#) n5 (sexism or sexist or ableis* or racism or racist or (racial n3 abus*)) ) 661

S19 TI ( ((staff or employee* or work* or nurs* or doctor#) n6 ((gender or disabilit* or disabled) n4 (inequalit* or equalit*))) OR ((staff or employee* or nurs* or doctor?) n1 (aggressi* or abus*))) OR AB ( ((staff or employee* or work* or nurs* or doctor#) n6 ((gender or disabilit* or disabled) n4 (inequalit* or equalit*))) OR ((staff or employee* or nurs* or doctor?) n1 (aggressi* or abus*)) ) OR SU ( ((staff or employee* or work* or nurs* or doctor#) n6 ((gender or disabilit* or disabled) n4 (inequalit* or equalit*))) OR ((staff or employee* or nurs* or doctor?) n1 (aggressi* or abus*)) ) 1,978

S18 ((MH "Workplace Violence") or (MH "Aggression") or (MH "Prejudice+") or (MH "Discrimination+") or (MH "Dehumanization") OR (MH "Oppressed Group Behavior") OR (MH "Coercion")) AND ( (MH "Interprofessional Relations+") or (MH "Intraprofessional Relations")) 1,028

S17 TI ( ((staff or employee* or work* or nurs* or doctor#) n4 (hostil* or undermin* or discrimination or discriminatory or discriminated or humiliat*) ) OR ((staff or employee* or work* or nurs* or doctor#) n4 (WPV or violen* or victim* or coerci*) n3 among*) OR "abusive supervision" OR (workplace n2 (conflict* or aggressi* or abus*)) ) OR AB ( ((staff or employee* or work* or nurs* or doctor#) n4 (hostil* or undermin* or discrimination or discriminatory or discriminated or humiliat*) ) OR ((staff or employee* or work* or nurs* or doctor#) n4 (WPV or violen* or victim* or coerci*) n3 among*) OR "abusive supervision" OR (workplace n2 (conflict* or aggressi* or abus*)) ) OR SU ( ((staff or employee* or work* or nurs* or doctor#) n4 (hostil* or undermin* or discrimination or discriminatory or discriminated or humiliat*) ) OR ((staff or employee* or work* or nurs* or doctor#) n4 (WPV or violen* or victim* or coerci*) n3 among*) OR "abusive supervision" OR (workplace n2 (conflict* or aggressi* or abus*)) ) 3,111

S16 S9 AND S15 18,280

S15 S10 OR S11 OR S12 OR S13 OR S14 2,214,829

S14 (MH "Health Personnel+") or (MH "Students, Health Occupations+") or (MH "Internship and Residency") OR (MH "Education, Graduate") OR (MH "Teamwork") 694,847

S13 TI ( (student# n2 (health* or clinic* or medic*)) OR employee# or worker# or Staff or personnel or practitioner# or professional# or workforce* or workplace* or "work place*" or worksite* or "work site*" or "work setting*" OR team*) OR AB ( (student# n2 (health* or clinic* or medic*)) OR employee# or worker# or Staff or personnel or practitioner# or professional# or workforce* or workplace* or "work place*" or worksite* or "work site*" or "work setting*" OR team*) OR SU ( (student# n2 (health* or clinic* or medic*)) OR employee# or worker# or Staff or personnel or practitioner# or professional# or workforce* or workplace* or "work place*" or worksite* or "work site*" or "work setting*" OR team*) 1,086,560

S12 TI ( Pathologist# or P#ediatrician# or Physiatrist# or Psychiatrist# or Pulmonologist# or Radiographer# or Radiologist# OR assistant# or cleaner# or ancillary or porter# or auxillary or auxillaries or administrator# or secretary or secretaries or receptionist# or technician# ) OR AB ( Pathologist# or P#ediatrician# or Physiatrist# or Psychiatrist# or Pulmonologist# or Radiographer# or Radiologist# OR assistant# or cleaner# or ancillary or porter# or auxillary or auxillaries or administrator# or secretary or secretaries or receptionist# or technician# ) OR SU ( Pathologist# or P#ediatrician# or Physiatrist# or Psychiatrist# or Pulmonologist# or Radiographer# or Radiologist# OR assistant# or cleaner# or ancillary or porter# or auxillary or auxillaries or administrator# or secretary or secretaries or receptionist# or technician# ) 153,037

S11 TI ( Audiologist# or Anatomist# or Allergist# or An#esthetist# or An#esthesiologist# or Cardiologist# or Dieti#ian# or Endocrinologist# or Gastroenterologist# or GP# or Geriatrician# or Hospitalist# or Oncologist# OR Ophthalmologist# or Otolaryngologist# ) OR AB ( Audiologist# or Anatomist# or Allergist# or An#esthetist# or An#esthesiologist# or Cardiologist# or Dieti#ian# or Endocrinologist# or Gastroenterologist# or GP# or Geriatrician# or Hospitalist# or Oncologist# OR Ophthalmologist# or Otolaryngologist# ) OR SU ( Audiologist# or Anatomist# or Allergist# or An#esthetist# or An#esthesiologist# or Cardiologist# or Dieti#ian# or Endocrinologist# or Gastroenterologist# or GP# or Geriatrician# or Hospitalist# or Oncologist# OR Ophthalmologist# or Otolaryngologist# ) 85,337

S10 TI ( ( nurs* or midwif* or midwiv or paramedic# or doctor# or physician# or clinician# or surgeon# or consultant# OR medic# or intern# or resident# or Therapist# or Pharmacist# or Optometrist# or Nutritionist# or Dentist# or Physiotherapist# ) ) OR AB ( ( nurs* or midwif* or midwiv or paramedic# or doctor# or physician# or clinician# or surgeon# or consultant# OR medic# or intern# or resident# or Therapist# or Pharmacist# or Optometrist# or Nutritionist# or Dentist# or Physiotherapist# ) ) OR SU ( ( nurs* or midwif* or midwiv or paramedic# or doctor# or physician# or clinician# or surgeon# or consultant# OR medic# or intern# or resident# or Therapist# or Pharmacist# or Optometrist# or Nutritionist# or Dentist# or Physiotherapist# ) ) 1,499,154

S9 S6 OR S7 OR S8 31,363

S8 TI ( ( ((lateral* or horizontal*) n2 violence) ) OR ( ((transgressive or disruptive or unprofessional) n2 behavio#r*) ) OR (professional* n2 misconduct) OR ( "negative behavio*" or "negative act" ) ) OR AB ( ( ((lateral* or horizontal*) n2 violence) ) OR ( ((transgressive or disruptive or unprofessional) n2 behavio#r*) ) OR (professional* n2 misconduct) OR ( "negative behavio*" or "negative act" ) ) OR SU ( ( ((lateral* or horizontal*) n2 violence) ) OR ( ((transgressive or disruptive or unprofessional) n2 behavio#r*) ) OR (professional* n2 misconduct) OR ( "negative behavio*" or "negative act" ) ) 11,945

S7 TI ( bully* or harass* or intimidat* or micro-aggress* or microaggress* or incivil* or uncivil* or rude* or mistreat* or mobbing or hazing or gaslight* or malic* or (Hidden n5 (aggressi* or abus* or violenc*))) OR AB ( bully* or harass* or intimidat* or micro-aggress* or microaggress* or incivil* or uncivil* or rude* or mistreat* or mobbing or hazing or gaslight* or malic* or (Hidden n5 (aggressi* or abus* or violenc*))) OR SU ( bully* or harass* or intimidat* or micro-aggress* or microaggress* or incivil* or uncivil* or rude* or mistreat* or mobbing or hazing or gaslight* or malic* or (Hidden n5 (aggressi* or abus* or violenc*))) 18,468

S6 (MH "Disruptive Behavior") or (MH "Bullying+") OR (MH "Emotional Abuse") OR (MH "Verbal Abuse") or (MH "Sexual Harassment") or (MH "Professional Misconduct") or (MH "Incivility") or (MH "Scapegoating") 22,192

S5 S1 OR S2 OR S3 OR S4 505,019

S4 TI ( ( (critical* n2 (care or ill*)) ) OR ( (urgent n2 (care or service* or medic*)) ) OR "intensive care" OR paramedic*) OR AB ( ( (critical* n2 (care or ill*)) ) OR ( (urgent n2 (care or service* or medic*)) ) OR "intensive care" OR paramedic*) OR SU ( ( (critical* n2 (care or ill*)) ) OR ( (urgent n2 (care or service* or medic*)) ) OR "intensive care" OR paramedic*) 165,198

S3 TI ( ( ((trauma* or ambulan*) n4 (care or service* or ill* or unit* or centre* or centre* or department* or setting)) ) ) OR AB ( ( ((trauma* or ambulan*) n4 (care or service* or ill* or unit* or centre* or centre* or department* or setting)) ) ) OR SU ( ( ((trauma* or ambulan*) n4 (care or service* or ill* or unit* or centre* or centre* or department* or setting)) ) ) 18,725

S2 TI ( ( ((emergenc* or acute*) n4 (care or service* or health* or ill* or treat* or medic* or unit* or centre* or centre* or department* or setting* or ward#)) ) ) OR AB ( ( ((emergenc* or acute*) n4 (care or service* or health* or ill* or treat* or medic* or unit* or centre* or centre* or department* or setting* or ward#)) ) ) OR SU ( ( ((emergenc* or acute*) n4 (care or service* or health* or ill* or treat* or medic* or unit* or centre* or centre* or department* or setting* or ward#)) ) ) 246,695

S1 (MH "Emergency Medical Services+") or (MH "Emergency Treatment+") or (MH "Emergency Care") or (MH "Airway Management+") or (MH "Ambulatory Care") OR (MH "Acute Care") or (MH "Critical Care+") OR (MH "Perioperative Care") OR (MH "Preoperative Care+") or (MH "Critical Care Nursing+") or (MH "Emergency Nursing+") 303,236

1. Embase Classic+Embase (Ovid) 1947 to 2022 February 11

Date searched: 14-02-2022

Records found: 1319

1 emergency health service/ or emergency medical dispatch/ or hospital emergency service/ or psychiatric emergency service/ 118852

2 emergency treatment/ or evidence based emergency medicine/ 18594

3 emergency care/ or advanced trauma life support/ or emergency ward/ 219986

4 respiration control/ or exp assisted ventilation/ or exp artificial ventilation/ 302732

5 exp ambulatory care/ or exp intensive care/ 817957

6 perioperative nursing/ or exp perioperative period/ or exp preoperative care/ 111940

7 exp hotline/ or poison center/ or exp ambulance/ 22714

8 exp intensive care nursing/ or emergency nursing/ 9915

9 (emergenc* adj5 (care or service* or health* or ill* or treat* or medic* or unit* or centre* or centre* or department* or setting*)).tw,kf. 297016

10 (acute* adj5 (care or service* or health* or ill* or treat* or medic* or unit* or centre* or centre* or department* or setting* or ward?)).tw,kf. 332752

11 (trauma* adj5 (care or service* or ill* or unit* or centre* or centre* or department*)).tw,kf. 36375

12 (ambula* adj5 (care or service* or unit* or centre* or centre* or department* or setting*)).tw,kf. 38326

13 (critical* adj2 (care or ill*)).tw,kf. 145798

14 (urgent adj3 (care or service* or medic*)).tw,kf. 8305

15 "intensive care".tw,kf. 255322

16 paramedic*.tw,kf. 13838

17 or/1-16 [Acute Care or Ambulance services] 1840430

18 agonistic behavior/ 193

19 exp bullying/ 9218

20 disruptive behavior/ 3154

21 harassment/ or non-sexual harassment/ or exp online harassment/ or exp sexual harassment/ 5032

22 incivility/ 363

23 microaggression/ 181

24 professional misconduct/ 3804

25 exp hostility/ 14279

26 bully*.tw,kf. 8592

27 harass*.tw,kf. 5336

28 intimidat*.tw,kf. 1973

29 (lateral* adj2 violence).tw,kf. 113

30 (horizontal* adj2 violence).tw,kf. 153

31 (transgressive adj3 behavio?r*).tw,kf. 46

32 (disruptive adj3 behavio?r*).tw,kf. 5605

33 (unprofessional adj3 behavio?r*).tw,kf. 479

34 (micro-aggress* or microaggress*).tw,kf. 562

35 incivil*.tw,kf. 688

36 uncivil*.tw,kf. 217

37 rude*.tw,kf. 1299

38 mistreat*.tw,kf. 3110

39 (professional* adj3 misconduct).tw,kf. 363

40 mobbing.tw,kf. 525

41 (negative behavio* or negative act?).tw,kf. 2078

42 hazing.tw,kf. 97

43 (gaslight* or malic*).tw,kf. 9947

44 (Hidden adj5 (aggressi* or abus* or violenc*)).tw,kf. 247

45 or/18-44 [Unprofessional behaviours] 61184

46 exp health care personnel/ 1827002

47 exp health student/ 129944

48 exp medical education/ 364812

49 public relations/ 63422

50 workplace/ 48445

51 teamwork/ 20182

52 (nurs* or midwif* or midwiv*).tw,kf. 620312

53 paramedic?.tw,kf. 9205

54 (doctor? or physician? or clinician? or surgeon? or consultant?).tw,kf. 1496063

55 (student? adj2 (medic* or health* or clinic*)).tw,kf. 88543

56 intern?.tw,kf. 16380

57 resident?.tw,kf. 239903

58 (Therapist? or Pharmacist? or Optometrist? or Nutritionist? or Dentist? or Physiotherapist?).tw,kf. 218724

59 (Audiologist? or Anatomist? or Allergist? or An?esthetist? or An?esthesiologist? or Cardiologist? or Dieti#ian? or Endocrinologist? or Gastroenterologist? or GP? or Geriatrician? or Hospitalist? or Oncologist?).tw,kf. 362665

60 (Ophthalmologist? or Otolaryngologist? or Pathologist? or P?ediatrician? or Physiatrist? or Psychiatrist? or Pulmonologist? or Radiographer? or Radiologist?).tw,kf. 281398

61 medic?.tw,kf. 42012

62 assistant?.tw,kf. 41928

63 (cleaner? or ancillary or porter?).tw,kf. 35085

64 (auxillary or auxillaries or administrator? or secretary or secretaries or receptionist? or technician?).tw,kf. 57136

65 (employee? or worker? or staff or personnel or practitioner? or professional? or workforce* or team*).tw,kf. /freq=2 573697

66 (workplace* or "work place*" or worksite* or "work site*" or "work setting*").tw,kf. 70577

67 or/46-66 [Staff] 4107447

68 45 and 67 [UB and Staff search 1] 18031

69 public relations/ and (aggression/ or aggressiveness/ or prejudice/ or exp social discrimination/ or coercion/) 419

70 ((staff or employee* or work* or nurs* or doctor?) adj8 (WPV or violen*) adj5 among*).tw,kf. 597

71 ((staff or employee* or work* or nurs* or doctor?) adj8 Victim* adj5 among*).tw,kf. 131

72 ((staff or employee* or work* or nurs* or doctor?) adj8 (humiliat* or hostil*)).tw,kf. 734

73 ((staff or employee* or work* or nurs* or doctor?) adj3 undermin*).tw,kf. 368

74 ((staff or employee* or work* or nurs* or doctor?) adj6 (discrimination or discriminatory or discriminated)).tw,kf. 2949

75 "abusive supervision".tw,kf. 121

76 (workplace adj3 (conflict* or aggressi* or abus*)).tw,kf. 634

77 ((staff or employee* or work* or nurs* or doctor?) adj5 (gender adj2 (inequalit* or equalit*))).tw,kf. 191

78 ((staff or employee* or work* or nurs* or doctor?) adj5 (racism or racist or (racial adj3 abus*))).tw,kf. 355

79 ((staff or employee* or work* or nurs* or doctor?) adj5 (sexism or sexist)).tw,kf. 80

80 ((staff or employee* or work* or nurs* or doctor?) adj8 ((disabilit* or disabled) adj5 (inequalit* or equalit*))).tw,kf. 20

81 ((staff or employee* or work* or nurs* or doctor?) adj8 ableis*).tw,kf. 10

82 ((staff or employee* or work* or nurs* or doctor?) adj8 coerci* adj5 among*).tw,kf. 11

83 ((staff or employee* or nurs* or doctor?) adj1 (aggressi* or abus*)).tw,kf. 275

84 or/69-83 [UB and staff search 2] 6650

85 68 or 84 [UB among Staff final search] 23586

86 17 and 85 [UB among Staff in Acute Care] 1669

87 afghanistan/ or africa/ or "africa south of the sahara"/ or albania/ or algeria/ or andorra/ or angola/ or argentina/ or "antigua and barbuda"/ or armenia/ or exp azerbaijan/ or bahamas/ or bahrain/ or bangladesh/ or barbados/ or belarus/ or belize/ or benin/ or bhutan/ or bolivia/ or borneo/ or exp "bosnia and herzegovina"/ or botswana/ or exp brazil/ or brunei darussalam/ or bulgaria/ or burkina faso/ or burundi/ or cambodia/ or cameroon/ or cape verde/ or central africa/ or central african republic/ or chad/ or exp china/ or comoros/ or congo/ or cook islands/ or coted'ivoire/ or croatia/ or cuba/ or cyprus/ or democratic republic congo/ or djibouti/ or dominica/ or dominican republic/ or ecuador/ or el salvador/ or egypt/ or equatorial guinea/ or eritrea/ or eswatini/ or ethiopia/ or exp "federated states of micronesia"/ or fiji/ or gabon/ or gambia/ or exp "georgia (republic)"/ or ghana/ or grenada/ or guatemala/ or guinea/ or guinea-bissau/ or guyana/ or haiti/ or honduras/ or exp india/ or exp indonesia/ or iran/ or exp iraq/ or jamaica/ or jordan/ or kazakhstan/ or kenya/ or kiribati/ or kosovo/ or kuwait/ or kyrgyzstan/ or laos/ or lebanon/ or liechtenstein/ or lesotho/ or liberia/ or libyan arab jamahiriya/ or madagascar/ or malawi/ or exp malaysia/ or maldives/ or mali/ or malta/ or mauritania/ or mauritius/ or melanesia/ or moldova/ or monaco/ or mongolia/ or "montenegro (republic)"/ or morocco/ or mozambique/ or myanmar/ or namibia/ or nauru/ or nepal/ or nicaragua/ or niger/ or nigeria/ or niue/ or north africa/ or oman/ or exp pakistan/ or palau/ or palestine/ or panama/ or papua new guinea/ or paraguay/ or peru/ or philippines/ or polynesia/ or qatar/ or "republic of north macedonia"/ or romania/ or exp russian federation/ or rwanda/ or sahel/ or "saint kitts and nevis"/ or "saint lucia"/ or "saint vincent and the grenadines"/ or saudi arabia/ or senegal/ or exp serbia/ or seychelles/ or sierra leone/ or singapore/ or "sao tome and principe"/ or solomon islands/ or exp somalia/ or south africa/ or south asia/ or south sudan/ or exp southeast asia/ or sri lanka/ or sudan/ or suriname/ or syrian arab republic/ or taiwan/ or tajikistan/ or tanzania/ or thailand/ or timor-leste/ or togo/ or tonga/ or "trinidad andtobago"/ or tunisia/ or turkmenistan/ or tuvalu/ or uganda/ or exp ukraine/ or exp united arab emirates/ or uruguay/ or exp uzbekistan/ or vanuatu/ or venezuela/ or viet nam/ or western sahara/ or yemen/ or zambia/ or zimbabwe/ 1583231

88 "organisation for economic co-operation and development"/ 1912

89 exp australia/ or "australia and new zealand"/ or austria/ or baltic states/ or exp belgium/ or exp canada/ or chile/ or colombia/ or costa rica/ or czech republic/ or denmark/ or estonia/ or europe/ or exp finland/ or exp france/ or exp germany/ or greece/ or hungary/ or iceland/ or ireland/ or israel/ or exp italy/ or japan/ or korea/ or latvia/ or lithuania/ or luxembourg/ or exp mexico/ or netherlands/ or new zealand/ or north america/ or exp norway/ or poland/ or exp portugal/ or scandinavia/ or sweden/ or slovakia/ or slovenia/ or south korea/ or exp spain/ or switzerland/ or exp united kingdom/ or "turkey (republic)"/ or exp united states/ or western europe/ 3795776

90 european union/ 29352

91 developed country/ 34992

92 or/88-91 3826946

93 87 not 92 1435767

94 86 not 93 [non OECD countries removed] 1504

95 exp juvenile/ not exp adult/ 2726374

96 94 not 95 [Child studies removed] 1363

97 (elder mistreat* or elder abuse* or elder neglect*).tw,kf. 2267

98 elder abuse/ 1666

99 97 or 98 2722

100 96 not 99 [Elder abuse studies removed] 1319

Ovid MEDLINE(R) ALL 1946 to February 11, 2022

Date searched: 14-02-2022

Records found: 838

1 emergency medical services/ or advanced trauma life support care/ or call centers/ or emergency medical dispatch/ or emergency medical service communication systems/ 47416

2 exp airway management/ or exp emergency treatment/ or exp ambulatory care/ or exp critical care/ or exp perioperative care/ or exp preoperative care/ 457199

3 exp emergency service, hospital/ or emergency services, psychiatric/ or hotlines/ or poison control centers/ or exp "transportation of patients"/ or triage/ or critical care nursing/ or emergency nursing/ 129355

4 (emergenc* adj5 (care or service* or health* or ill* or treat* or medic* or unit* or centre* or centre* or department* or setting*)).tw,kf. 201058

5 (acute* adj5 (care or service* or health* or ill* or treat* or medic* or unit* or centre* or centre* or department* or setting* or ward?)).tw,kf. 220841

6 (trauma* adj5 (care or service* or ill* or unit* or centre* or centre* or department*)).tw,kf. 26703

7 (ambula* adj5 (care or service* or unit* or centre* or centre* or department* or setting*)).tw,kf. 26152

8 (critical* adj2 (care or ill*)).tw,kf. 92532

9 (urgent adj3 (care or service* or medic*)).tw,kf. 5316

10 "intensive care".tw,kf. 171685

11 paramedic*.tw,kf. 9163

12 or/1-11 [Acute Care or Ambulance services] 1064466

13 agonistic behavior/ 1779

14 exp bullying/ 5625

15 problem behavior/ 3242

16 exp harassment, non-sexual/ 5708

17 sexual harassment/ 2022

18 incivility/ 234

19 Professional Misconduct/ 3434

20 Hostility/ 5399

21 bully*.tw,kf. 7062

22 harass*.tw,kf. 4657

23 intimidat*.tw,kf. 1509

24 (lateral* adj2 violence).tw,kf. 100

25 (horizontal* adj2 violence).tw,kf. 146

26 (transgressive adj3 behavio?r*).tw,kf. 39

27 (disruptive adj3 behavio?r*).tw,kf. 4367

28 (unprofessional adj3 behavio?r*).tw,kf. 400

29 (micro-aggress* or microaggress*).tw,kf. 477

30 incivil*.tw,kf. 686

31 uncivil*.tw,kf. 215

32 rude*.tw,kf. 1194

33 mistreat*.tw,kf. 2451

34 (professional* adj3 misconduct).tw,kf. 303

35 mobbing.tw,kf. 418

36 hazing.tw,kf. 84

37 (negative behavio* or negative act?).tw,kf. 1716

38 (gaslight* or malic*).tw,kf. 8372

39 (Hidden adj5 (aggressi* or abus* or violenc*)).tw,kf. 195

40 or/13-39 [Unprofessional behaviours] 47372

41 exp Health Personnel/ 572761

42 exp Students, Health Occupations/ 79918

43 exp education, graduate/ or "internship and residency"/ or teaching rounds/ 94478

44 exp Interprofessional Relations/ 71806

45 exp Patient Care Team/ 71777

46 Workplace/ 26813

47 (nurs* or midwif* or midwiv*).tw,kf. 516983

48 paramedic?.tw,kf. 6046

49 (doctor? or physician? or clinician? or surgeon? or consultant?).tw,kf. 1005171

50 (student? adj2 (medic* or health* or clinic*)).tw,kf. 65931

51 intern?.tw,kf. 11187

52 resident?.tw,kf. 179417

53 (Therapist? or Pharmacist? or Optometrist? or Nutritionist? or Dentist? or Physiotherapist?).tw,kf. 132734

54 (Audiologist? or Anatomist? or Allergist? or An?esthetist? or An?esthesiologist? or Cardiologist? or Dieti#ian? or Endocrinologist? or Gastroenterologist? or GP? or Geriatrician? or Hospitalist? or Oncologist?).tw,kf. 234452

55 (Ophthalmologist? or Otolaryngologist? or Pathologist? or P?ediatrician? or Physiatrist? or Psychiatrist? or Pulmonologist? or Radiographer? or Radiologist?).tw,kf. 170783

56 medic?.tw,kf. 21509

57 assistant?.tw,kf. 28841

58 (cleaner? or ancillary or porter?).tw,kf. 24976

59 (auxillary or auxillaries or administrator? or secretary or secretaries or receptionist? or technician?).tw,kf. 41556

60 (employee? or worker? or Staff or personnel or practitioner? or professional? or workforce* or team*).tw,kf. /freq=2 414261

61 (workplace* or "work place*" or worksite* or "work site*" or "work setting*").tw,kf. 56083

62 or/41-61 [Staff] 2697074

63 40 and 62 [UB and Staff search 1] 13280

64 physician-nurse relations/ or interprofessional relations/ 54906

65 aggression/ or prejudice/ or Social Discrimination/ or ageism/ or gender equity/ or homophobia/ or exp racism/ or sexism/ or weight prejudice/ or xenophobia/ or Perceived Discrimination/ or Coercion/ 74909

66 64 and 65 [Aggression or prejudice among staff MeSH] 767

67 ((staff or employee* or work* or nurs* or doctor?) adj8 (WPV or violen*) adj5 among*).tw,kf. 562

68 ((staff or employee* or work* or nurs* or doctor?) adj8 coerci* adj5 among*).tw,kf. 13

69 ((staff or employee* or work* or nurs* or doctor?) adj8 Victim* adj5 among*).tw,kf. 109

70 ((staff or employee* or work* or nurs* or doctor?) adj8 (humiliat* or hostil*)).tw,kf. 570

71 ((staff or employee* or work* or nurs* or doctor?) adj3 undermin*).tw,kf. 337

72 ((staff or employee* or work* or nurs* or doctor?) adj6 (discrimination or discriminatory or discriminated)).tw,kf. 2538

73 "abusive supervision".tw,kf. 140

74 (workplace adj3 (conflict* or aggressi* or abus*)).tw,kf. 562

75 ((staff or employee* or work* or nurs* or doctor?) adj5 (gender adj2 (inequalit* or equalit*))).tw,kf. 210

76 ((staff or employee* or work* or nurs* or doctor?) adj5 (racism or racist or (racial adj3 abus*))).tw,kf. 331

77 ((staff or employee* or work* or nurs* or doctor?) adj5 (sexism or sexist)).tw,kf. 83

78 ((staff or employee* or work* or nurs* or doctor?) adj8 ((disabilit* or disabled) adj5 (inequalit* or equalit*))).tw,kf. 15

79 ((staff or employee* or work* or nurs* or doctor?) adj8 ableis*).tw,kf. 6

80 ((staff or employee* or nurs* or doctor?) adj1 (aggressi* or abus*)).tw,kf. 237

81 or/66-80 [UB and staff search 2] 6326

82 63 or 81 [UB among Staff final search] 18616

83 12 and 82 [UB among Staff in Acute Care] 1023

84 afghanistan/ or africa/ or africa, northern/ or africa, central/ or africa, eastern/ or "africa south of the sahara"/ or africa, southern/ or africa, western/ or albania/ or algeria/ or andorra/ or angola/ or "antigua and barbuda"/ or argentina/ or armenia/ or azerbaijan/ or bahamas/ or bahrain/ or bangladesh/ or barbados/ or belize/ or benin/ or bhutan/ or bolivia/ or borneo/ or "bosnia and herzegovina"/ or botswana/ or brazil/ or brunei/ or bulgaria/ or burkina faso/ or burundi/ or cabo verde/ or cambodia/ or cameroon/ or central african republic/ or chad/ or exp china/ or comoros/ or congo/ or cote d'ivoire/ or croatia/ or cuba/ or "democratic republic of the congo"/ or cyprus/ or djibouti/ or dominica/ or dominican republic/ or ecuador/ or egypt/ or el salvador/ or equatorial guinea/ or eritrea/ or eswatini/ or ethiopia/ or fiji/ or gabon/ or gambia/ or "georgia (republic)"/ or ghana/ or grenada/ or guatemala/ or guinea/ or guinea-bissau/ or guyana/ or haiti/ or honduras/ or independent state of samoa/ or exp india/ or indian ocean islands/ or indochina/ or indonesia/ or iran/ or iraq/ or jamaica/ or jordan/ or kazakhstan/ or kenya/ or kosovo/ or kuwait/ or kyrgyzstan/ or laos/ or lebanon/ or liechtenstein/ or lesotho/ or liberia/ or libya/ or madagascar/ or malaysia/ or malawi/ or mali/ or malta/ or mauritania/ or mauritius/ or mekong valley/ or melanesia/ or micronesia/ or monaco/ or mongolia/ or montenegro/ or morocco/ or mozambique/ or myanmar/ or namibia/ or nepal/ or nicaragua/ or niger/ or nigeria/ or oman/ or pakistan/ or palau/ or exp panama/ or papua new guinea/ or paraguay/ or peru/ or philippines/ or qatar/ or "republic of belarus"/ or "republic of north macedonia"/ or romania/ or exp russia/ or rwanda/ or "saint kitts and nevis"/ or saint lucia/ or "saint vincent and the grenadines"/ or "sao tome and principe"/ or saudi arabia/ or serbia/ or sierra leone/ or senegal/ or seychelles/ or singapore/ or somalia/ or south africa/ or south sudan/ or sri lanka/ or sudan/ or suriname/ or syria/ or taiwan/ or tajikistan/ or tanzania/ or thailand/ or timor-leste/ or togo/ or tonga/ or "trinidad and tobago"/ or tunisia/ or turkmenistan/ or uganda/ or ukraine/ or united arab emirates/ or uruguay/ or uzbekistan/ or vanuatu/ or venezuela/ or vietnam/ or west indies/ or yemen/ or zambia/ or zimbabwe/ 1199047

85 "Organisation for Economic Co-Operation and Development"/ 415

86 australasia/ or exp australia/ or austria/ or baltic states/ or belgium/ or exp canada/ or chile/ or colombia/ or costa rica/ or czech republic/ or exp denmark/ or estonia/ or europe/ or finland/ or exp france/ or exp germany/ or greece/ or hungary/ or iceland/ or ireland/ or israel/ or exp italy/ or exp japan/ or korea/ or latvia/ or lithuania/ or luxembourg/ or mexico/ or netherlands/ or new zealand/ or north america/ or exp norway/ or poland/ or portugal/ or exp "republic of korea"/ or "scandinavian and nordic countries"/ or slovakia/ or slovenia/ or spain/ or sweden/ or switzerland/ or turkey/ or exp united kingdom/ or exp united states/ 3382217

87 European Union/ 17106

88 Developed Countries/ 21067

89 or/85-88 3397472

90 84 not 89 [OECD search filter NICE 2021] 1112292

91 83 not 90 [UB among Acute Care Staff with non-OECD countries removed] 931

92 (exp Child/ or Adolescent/ or exp Infant/) not exp Adult/ 2021427

93 91 not 92 [Child studies removed] 863

94 (elder mistreat* or elder abuse* or elder neglect*).tw,kf. 1880

95 93 not 94 [Elder abuse studies removed] 838

HMIC Health Management Information Consortium (Ovid) 1979 to November 2021

Date searched: 14-02-2022

Records found: 58

1 exp emergency health services/ 3422

2 exp emergency treatment/ 872

3 hospital departments/ or exp accident & emergency departments/ or exp acute units/ or exp critical care units/ or exp intensive care units/ or exp pre operative units/ or exp trauma centres/ 1963

4 accident & emergency nursing/ or ambulance service nursing/ or intensive care nursing/ 149

5 accident & emergency nursing/ or ambulance service nursing/ or intensive care nursing/ or triage/ 442

6 (acute* adj5 (care or service* or health* or ill* or treat* or medic* or unit* or centre* or centre* or department* or setting* or ward?)).tw,hw. 6620

7 (trauma* adj5 (care or service* or ill* or unit* or centre* or centre* or department*)).tw,hw. 440

8 (ambula* adj5 (care or service* or unit* or centre* or centre* or department* or setting*)).tw,hw. 2109

9 (critical* adj2 (care or ill*)).tw,hw. 1076

10 (urgent adj3 (care or service* or medic*)).tw,hw. 455

11 "intensive care".tw,hw. 2518

12 paramedic*.tw,hw. 484

13 or/1-12 [Acute Care or Ambulance services] 15204

14 bullying/ 319

15 exp harassment/ 488

16 malpractice/ 276

17 hostility/ 12

18 exp discrimination/ 2100

19 bully*.tw,hw. 430

20 harass*.tw,hw. 435

21 intimidat*.tw,hw. 107

22 (lateral* adj2 violence).tw,hw. 2

23 (horizontal* adj2 violence).tw,hw. 6

24 (transgressive adj3 behavio?r*).tw,hw. 0

25 (disruptive adj3 behavio?r*).tw,hw. 81

26 (unprofessional adj3 behavio?r*).tw,hw. 11

27 (micro-aggress* or microaggress*).tw,hw. 2

28 incivil*.tw,hw. 17

29 uncivil*.tw,hw. 4

30 rude*.tw,hw. 29

31 mistreat*.tw,hw. 51

32 (professional* adj3 misconduct).tw,hw. 31

33 mobbing.tw,hw. 2

34 (negative behavio* or negative act?).tw,hw. 34

35 hazing.tw,kf. 0

36 (gaslight* or malic*).tw,hw. 17

37 (Hidden adj5 (aggressi* or abus* or violenc*)).tw,hw. 18

38 or/14-37 [Unprofessional behaviours] 3329

39 exp health service staff/ 55488

40 exp medical education/ 3378

41 exp interprofessional relations/ 2548

42 exp teamwork/ 1159

43 workplace/ 80

44 (nurs* or midwif* or midwiv*).tw,hw. 48975

45 paramedic?.tw,hw. 270

46 (doctor? or physician? or clinician? or surgeon? or consultant?).tw,hw. 31472

47 (student? adj2 (medic* or health* or clinic*)).tw,hw. 1471

48 intern?.tw,hw. 61

49 resident?.tw,hw. 4966

50 (Therapist? or Pharmacist? or Optometrist? or Nutritionist? or Dentist? or Physiotherapist?).tw,hw. 8803

51 (Audiologist? or Anatomist? or Allergist? or An?esthetist? or An?esthesiologist? or Cardiologist? or Dieti#ian? or Endocrinologist? or Gastroenterologist? or GP? or Geriatrician? or Hospitalist? or Oncologist?).tw,hw. 14609

52 (Ophthalmologist? or Otolaryngologist? or Pathologist? or P?ediatrician? or Physiatrist? or Psychiatrist? or Pulmonologist? or Radiographer? or Radiologist?).tw,hw. 2785

53 medic?.tw,hw. 275

54 assistant?.tw,hw. 2110

55 (cleaner? or ancillary or porter?).tw,hw. 681

56 (auxillary or auxillaries or administrator? or secretary or secretaries or receptionist? or technician?).tw,hw. 4510

57 (employee? or worker? or Staff or personnel or practitioner? or professional? or workforce* or team*).tw,hw. 115226

58 (workplace* or "work place*" or worksite* or "work site*" or "work setting*").tw,hw. 3499

59 or/39-58 [Staff] 166259

60 38 and 59 [UB and Staff search 1] 1743

61 exp aggressive behaviour/ and (nurse doctor relations/ or interprofessional relations/) 3

62 exp prejudice/ and (nurse doctor relations/ or interprofessional relations/) 2

63 ((staff or employee* or work* or nurs* or doctor?) adj8 (WPV or violen*) adj5 among*).tw,hw. 15

64 ((staff or employee* or work* or nurs* or doctor?) adj8 Victim* adj5 among*).tw,hw. 0

65 ((staff or employee* or work* or nurs* or doctor?) adj8 (humiliat* or hostil*)).tw,hw. 76

66 ((staff or employee* or work* or nurs* or doctor?) adj3 undermin*).tw,hw. 71

67 ((staff or employee* or work* or nurs* or doctor?) adj6 (discrimination or discriminatory or discriminated)).tw,hw. 364

68 "abusive supervision".tw,hw. 0

69 (workplace adj3 (conflict* or aggressi* or abus*)).tw,hw. 35

70 ((staff or employee* or work* or nurs* or doctor?) adj5 (gender adj2 (inequalit* or equalit*))).tw,hw. 32

71 ((staff or employee* or work* or nurs* or doctor?) adj5 (racism or racist or (racial adj3 abus*))).tw,hw. 167

72 ((staff or employee* or work* or nurs* or doctor?) adj5 (sexism or sexist)).tw,hw. 18

73 ((staff or employee* or work* or nurs* or doctor?) adj8 ((disabilit* or disabled) adj5 (inequalit* or equalit*))).tw,hw. 18

74 ((staff or employee* or work* or nurs* or doctor?) adj8 ableis*).tw,hw. 0

75 ((staff or employee* or work* or nurs* or doctor?) adj8 coerci* adj5 among*).tw,hw. 1

76 ((staff or employee* or nurs* or doctor?) adj1 (aggressi* or abus*)).tw,hw. 31

77 or/61-76 [UB and staff search 2] 800

78 60 or 77 [UB and Staff final search] 2224

79 78 and 13 [UB among Acute Care Staff] 59

80 ((child* or infant* or baby or babies or preterm* or adolesc* or newborn* or pediatric* or paediatri* or neonate* or teen* or schoolchild*) not (adult or elderly or geriatric*)).ti. 21099

81 79 not 80 [Child studies removed] 58

82 (elder mistreat* or elder abuse* or elder neglect*).tw,hw. 301

83 81 not 82 [Elder abuse studies removed] 58

NICE Evidence Search <https://www.evidence.nhs.uk/>

Date searched: 15-02-2022

Records found: 79

("workplace bullying" or "workplace discrimination" or "unprofessional behaviour" or "staff bullying" or "bullying staff" or "staff harassment" or "staff discrimination") and (health or healthcare or hospital or medicine or medical or nurse or doctor) limited to filters

Filter applied: Area of interest : Clinical OR Area of interest: Commissioning and Management

Patient Safety Network <https://psnet.ahrq.gov/>

Date searched: 15-02-2022

Records found: 83

abuse bullying unprofessional harassment discrimination microaggression incivility intimidation humiliation hostility mobbing hazing gaslighting malicious

Limited to Ambulatory Care setting OR Limit to Hospital setting

#### 3. USA update search Aug 2022

Sources searched:

- CINAHL (EBSCOhost)
- Embase Classic+Embase (Ovid) 1947 to 2022 August 24
- Ovid MEDLINE(R) ALL 1946 to August 24, 2022
- Google Scholar
- Patient Safety Network <https://psnet.ahrq.gov/>

CINAHL (EBSCOhost)

Date searched: 25-08-2022

Records found: 357

# Query Results

S39 S37 AND S38 357

S38 (MH "United States+") OR AF ( USA or "united states" or america* ) OR TI ( USA or "united states" or america* ) OR AB ( USA or "united states" or america* ) 1,752,918

S37 S35 NOT S36 1,316

S36 TX ( ("elder mistreat*" or "elder abuse*" or "elder neglect*") ) 4,919

S35 S33 NOT S34 1,360

S34 ( ( (MH "Child") OR (MH "Adolescence+") OR (MH "Minors (Legal)") ) NOT (MH "Adult+") ) 483,829

S33 S22 NOT S32 1,436

S32 S28 NOT S31 381,030

S31 S29 OR S30 1,714,259

S30 (MH "Australia+") OR (MH "Europe") OR (MH "Austria") OR (MH "Baltic States+") OR (MH "Belgium") OR (MH "Canada+") OR (MH "Chile") OR (MH "Colombia") OR (MH "Costa Rica") OR (MH "Czech Republic") OR (MH "Scandinavia+") OR (MH "France") OR (MH "Germany+") OR (MH "Greece") OR (MH "Hungary") OR (MH "Iceland") OR (MH "Ireland") OR (MH "Italy") OR (MH "Israel") OR (MH "Japan") OR (MH "South Korea") OR (MH "Luxembourg") OR (MH "Mexico") OR (MH "Netherlands") OR (MH "New Zealand") OR (MH "North America") OR (MH "Poland") OR (MH "Portugal") OR (MH "Slovakia") OR (MH "Slovenia") OR (MH "Spain") OR (MH "Switzerland") OR (MH "Turkey") OR (MH "United Kingdom+") OR (MH "United States+") 1,707,539

S29 (MH "Developed Countries") or (MH "European Union") or (MH "Organisation for Economic Co-Operation and Development") 10,460

S28 (S23 OR S24 OR S25 OR S26 OR S27) 419,922

S27 (MH "Africa+") 95,616

S26 (MH "Asia, Southeastern+") OR (MH "China+") OR (MH "Hong Kong") OR (MH "Macao") OR (MH "Mongolia") OR (MH "North Korea") OR (MH "Taiwan") OR (MH "Atlantic Islands") OR (MH "Indian Ocean Islands+") OR (MH "Melanesia+") OR (MH "Micronesia+") OR (MH "Polynesia+") 132,915

S25 (MH "Bangladesh") OR (MH "Bhutan") OR (MH "India") OR (MH "Yemen") OR (MH "United Arab Emirates") OR (MH "Syria") OR (MH "Saudi Arabia") OR (MH "Qatar") OR (MH "Oman") OR (MH "Lebanon") OR (MH "Kuwait") OR (MH "Jordan") OR (MH "Iraq") OR (MH "Iran") OR (MH "Bahrain") OR (MH "Afghanistan") OR (MH "Nepal") OR (MH "Pakistan") OR (MH "Sri Lanka") OR (MH "Asia, Central+") 113,865

S24 (MH "Argentina") OR (MH "Bolivia") OR (MH "Brazil") OR (MH "Ecuador") OR (MH "French Guiana") OR (MH "Guyana") OR (MH "Paraguay") OR (MH "Peru") OR (MH "Suriname") OR (MH "Uruguay") OR (MH "Venezuela") OR (MH "Belize") OR (MH "El Salvador") OR (MH "Guatemala") OR (MH "Honduras") OR (MH "Nicaragua") OR (MH "Panama+") or (MH "West Indies+") 74,518

S23 (MH "Albania") OR (MH "Andorra") OR (MH "Armenia") OR (MH "Azerbaijan") or (MH "Byelarus") OR (MH "Bosnia-Herzegovina") OR (MH "Croatia") OR (MH "Bulgaria") OR (MH "Georgia (Republic)") OR (MH "Gibraltar") OR (MH "Liechtenstein") OR (MH "Macedonia (Republic)") OR (MH "Moldova") OR (MH "Monaco") OR (MH "Romania") OR (MH "Russia") OR (MH "San Marino") OR (MH "Serbia") OR (MH "Ukraine") OR (MH "Yugoslavia") 12,299

S22 S5 AND S21 1,557

S21 S16 OR S17 OR S18 OR S19 OR S20 24,298

S20 TI ( (staff or employee* or work* or nurs* or doctor#) n5 (sexism or sexist or ableis* or racism or racist or (racial n3 abus*)) ) OR AB ( (staff or employee* or work* or nurs* or doctor#) n5 (sexism or sexist or ableis* or racism or racist or (racial n3 abus*)) ) OR SU ( (staff or employee* or work* or nurs* or doctor#) n5 (sexism or sexist or ableis* or racism or racist or (racial n3 abus*)) ) 732

S19 TI ( ((staff or employee* or work* or nurs* or doctor#) n6 ((gender or disabilit* or disabled) n4 (inequalit* or equalit*))) OR ((staff or employee* or nurs* or doctor?) n1 (aggressi* or abus*))) OR AB ( ((staff or employee* or work* or nurs* or doctor#) n6 ((gender or disabilit* or disabled) n4 (inequalit* or equalit*))) OR ((staff or employee* or nurs* or doctor?) n1 (aggressi* or abus*)) ) OR SU ( ((staff or employee* or work* or nurs* or doctor#) n6 ((gender or disabilit* or disabled) n4 (inequalit* or equalit*))) OR ((staff or employee* or nurs* or doctor?) n1 (aggressi* or abus*)) ) 2,017

S18 ((MH "Workplace Violence") or (MH "Aggression") or (MH "Prejudice+") or (MH "Discrimination+") or (MH "Dehumanization") OR (MH "Oppressed Group Behavior") OR (MH "Coercion")) AND ( (MH "Interprofessional Relations+") or (MH "Intraprofessional Relations")) 1,053

S17 TI ( ((staff or employee* or work* or nurs* or doctor#) n4 (hostil* or undermin* or discrimination or discriminatory or discriminated or humiliat*) ) OR ((staff or employee* or work* or nurs* or doctor#) n4 (WPV or violen* or victim* or coerci*) n3 among*) OR "abusive supervision" OR (workplace n2 (conflict* or aggressi* or abus*)) ) OR AB ( ((staff or employee* or work* or nurs* or doctor#) n4 (hostil* or undermin* or discrimination or discriminatory or discriminated or humiliat*) ) OR ((staff or employee* or work* or nurs* or doctor#) n4 (WPV or violen* or victim* or coerci*) n3 among*) OR "abusive supervision" OR (workplace n2 (conflict* or aggressi* or abus*)) ) OR SU ( ((staff or employee* or work* or nurs* or doctor#) n4 (hostil* or undermin* or discrimination or discriminatory or discriminated or humiliat*) ) OR ((staff or employee* or work* or nurs* or doctor#) n4 (WPV or violen* or victim* or coerci*) n3 among*) OR "abusive supervision" OR (workplace n2 (conflict* or aggressi* or abus*)) ) 3,244

S16 S9 AND S15 18,721

S15 S10 OR S11 OR S12 OR S13 OR S14 2,270,521

S14 (MH "Health Personnel+") or (MH "Students, Health Occupations+") or (MH "Internship and Residency") OR (MH "Education, Graduate") OR (MH "Teamwork") 712,763

S13 TI ( (student# n2 (health* or clinic* or medic*)) OR employee# or worker# or Staff or personnel or practitioner# or professional# or workforce* or workplace* or "work place*" or worksite* or "work site*" or "work setting*" OR team*) OR AB ( (student# n2 (health* or clinic* or medic*)) OR employee# or worker# or Staff or personnel or practitioner# or professional# or workforce* or workplace* or "work place*" or worksite* or "work site*" or "work setting*" OR team*) OR SU ( (student# n2 (health* or clinic* or medic*)) OR employee# or worker# or Staff or personnel or practitioner# or professional# or workforce* or workplace* or "work place*" or worksite* or "work site*" or "work setting*" OR team*) 1,118,297

S12 TI ( Pathologist# or P#ediatrician# or Physiatrist# or Psychiatrist# or Pulmonologist# or Radiographer# or Radiologist# OR assistant# or cleaner# or ancillary or porter# or auxillary or auxillaries or administrator# or secretary or secretaries or receptionist# or technician# ) OR AB ( Pathologist# or P#ediatrician# or Physiatrist# or Psychiatrist# or Pulmonologist# or Radiographer# or Radiologist# OR assistant# or cleaner# or ancillary or porter# or auxillary or auxillaries or administrator# or secretary or secretaries or receptionist# or technician# ) OR SU ( Pathologist# or P#ediatrician# or Physiatrist# or Psychiatrist# or Pulmonologist# or Radiographer# or Radiologist# OR assistant# or cleaner# or ancillary or porter# or auxillary or auxillaries or administrator# or secretary or secretaries or receptionist# or technician# ) 157,381

S11 TI ( Audiologist# or Anatomist# or Allergist# or An#esthetist# or An#esthesiologist# or Cardiologist# or Dieti#ian# or Endocrinologist# or Gastroenterologist# or GP# or Geriatrician# or Hospitalist# or Oncologist# OR Ophthalmologist# or Otolaryngologist# ) OR AB ( Audiologist# or Anatomist# or Allergist# or An#esthetist# or An#esthesiologist# or Cardiologist# or Dieti#ian# or Endocrinologist# or Gastroenterologist# or GP# or Geriatrician# or Hospitalist# or Oncologist# OR Ophthalmologist# or Otolaryngologist# ) OR SU ( Audiologist# or Anatomist# or Allergist# or An#esthetist# or An#esthesiologist# or Cardiologist# or Dieti#ian# or Endocrinologist# or Gastroenterologist# or GP# or Geriatrician# or Hospitalist# or Oncologist# OR Ophthalmologist# or Otolaryngologist# ) 88,249

S10 TI ( ( nurs* or midwif* or midwiv or paramedic# or doctor# or physician# or clinician# or surgeon# or consultant# OR medic# or intern# or resident# or Therapist# or Pharmacist# or Optometrist# or Nutritionist# or Dentist# or Physiotherapist# ) ) OR AB ( ( nurs* or midwif* or midwiv or paramedic# or doctor# or physician# or clinician# or surgeon# or consultant# OR medic# or intern# or resident# or Therapist# or Pharmacist# or Optometrist# or Nutritionist# or Dentist# or Physiotherapist# ) ) OR SU ( ( nurs* or midwif* or midwiv or paramedic# or doctor# or physician# or clinician# or surgeon# or consultant# OR medic# or intern# or resident# or Therapist# or Pharmacist# or Optometrist# or Nutritionist# or Dentist# or Physiotherapist# ) ) 1,532,802

S9 S6 OR S7 OR S8 32,395

S8 TI ( ( ((lateral* or horizontal*) n2 violence) ) OR ( ((transgressive or disruptive or unprofessional) n2 behavio#r*) ) OR (professional* n2 misconduct) OR ( "negative behavio*" or "negative act" ) ) OR AB ( ( ((lateral* or horizontal*) n2 violence) ) OR ( ((transgressive or disruptive or unprofessional) n2 behavio#r*) ) OR (professional* n2 misconduct) OR ( "negative behavio*" or "negative act" ) ) OR SU ( ( ((lateral* or horizontal*) n2 violence) ) OR ( ((transgressive or disruptive or unprofessional) n2 behavio#r*) ) OR (professional* n2 misconduct) OR ( "negative behavio*" or "negative act" ) ) 12,059

S7 TI ( bully* or harass* or intimidat* or micro-aggress* or microaggress* or incivil* or uncivil* or rude* or mistreat* or mobbing or hazing or gaslight* or malic* or (Hidden n5 (aggressi* or abus* or violenc*))) OR AB ( bully* or harass* or intimidat* or micro-aggress* or microaggress* or incivil* or uncivil* or rude* or mistreat* or mobbing or hazing or gaslight* or malic* or (Hidden n5 (aggressi* or abus* or violenc*))) OR SU ( bully* or harass* or intimidat* or micro-aggress* or microaggress* or incivil* or uncivil* or rude* or mistreat* or mobbing or hazing or gaslight* or malic* or (Hidden n5 (aggressi* or abus* or violenc*))) 19,245

S6 (MH "Disruptive Behavior") or (MH "Bullying+") OR (MH "Emotional Abuse") OR (MH "Verbal Abuse") or (MH "Sexual Harassment") or (MH "Professional Misconduct") or (MH "Incivility") or (MH "Scapegoating") 22,843

S5 S1 OR S2 OR S3 OR S4 520,545

S4 TI ( ( (critical* n2 (care or ill*)) ) OR ( (urgent n2 (care or service* or medic*)) ) OR "intensive care" OR paramedic*) OR AB ( ( (critical* n2 (care or ill*)) ) OR ( (urgent n2 (care or service* or medic*)) ) OR "intensive care" OR paramedic*) OR SU ( ( (critical* n2 (care or ill*)) ) OR ( (urgent n2 (care or service* or medic*)) ) OR "intensive care" OR paramedic*) 170,862

S3 TI ( ( ((trauma* or ambulan*) n4 (care or service* or ill* or unit* or centre* or centre* or department* or setting)) ) ) OR AB ( ( ((trauma* or ambulan*) n4 (care or service* or ill* or unit* or centre* or centre* or department* or setting)) ) ) OR SU ( ( ((trauma* or ambulan*) n4 (care or service* or ill* or unit* or centre* or centre* or department* or setting)) ) ) 19,577

S2 TI ( ( ((emergenc* or acute*) n4 (care or service* or health* or ill* or treat* or medic* or unit* or centre* or centre* or department* or setting* or ward#)) ) ) OR AB ( ( ((emergenc* or acute*) n4 (care or service* or health* or ill* or treat* or medic* or unit* or centre* or centre* or department* or setting* or ward#)) ) ) OR SU ( ( ((emergenc* or acute*) n4 (care or service* or health* or ill* or treat* or medic* or unit* or centre* or centre* or department* or setting* or ward#)) ) ) 254,587

S1 (MH "Emergency Medical Services+") or (MH "Emergency Treatment+") or (MH "Emergency Care") or (MH "Airway Management+") or (MH "Ambulatory Care") OR (MH "Acute Care") or (MH "Critical Care+") OR (MH "Perioperative Care") OR (MH "Preoperative Care+") or (MH "Critical Care Nursing+") or (MH "Emergency Nursing+") 310,494

Embase Classic+Embase (Ovid) 1947 to 2022 August 24

Date searched: 25-08-2022

Records found: 585

1 emergency health service/ or emergency medical dispatch/ or hospital emergency service/ or psychiatric emergency service/ (122336)

2 emergency treatment/ or evidence based emergency medicine/ (18782)

3 emergency care/ or advanced trauma life support/ or emergency ward/ (233211)

4 respiration control/ or exp assisted ventilation/ or exp artificial ventilation/ (319367)

5 exp ambulatory care/ or exp intensive care/ (850847)

6 perioperative nursing/ or exp perioperative period/ or exp preoperative care/ (116685)

7 exp hotline/ or poison center/ or exp ambulance/ (23525)

8 exp intensive care nursing/ or emergency nursing/ (9995)

9 (emergenc* adj5 (care or service* or health* or ill* or treat* or medic* or unit* or centre* or centre* or department* or setting*)).tw,kf. (311715)

10 (acute* adj5 (care or service* or health* or ill* or treat* or medic* or unit* or centre* or centre* or department* or setting* or ward?)).tw,kf. (343867)

11 (trauma* adj5 (care or service* or ill* or unit* or centre* or centre* or department*)).tw,kf. (38174)

12 (ambula* adj5 (care or service* or unit* or centre* or centre* or department* or setting*)).tw,kf. (39628)

13 (critical* adj2 (care or ill*)).tw,kf. (152661)

14 (urgent adj3 (care or service* or medic*)).tw,kf. (8970)

15 "intensive care".tw,kf. (267368)

16 paramedic*.tw,kf. (14376)

17 or/1-16 [Acute Care or Ambulance services] (1915208)

18 agonistic behavior/ (215)

19 exp bullying/ (9865)

20 disruptive behavior/ (3294)

21 harassment/ or non-sexual harassment/ or exp online harassment/ or exp sexual harassment/ (5440)

22 incivility/ (409)

23 microaggression/ (260)

24 professional misconduct/ (3824)

25 exp hostility/ (14620)

26 bully*.tw,kf. (9103)

27 harass*.tw,kf. (5669)

28 intimidat*.tw,kf. (2045)

29 (lateral* adj2 violence).tw,kf. (119)

30 (horizontal* adj2 violence).tw,kf. (156)

31 (transgressive adj3 behavio?r*).tw,kf. (48)

32 (disruptive adj3 behavio?r*).tw,kf. (5760)

33 (unprofessional adj3 behavio?r*).tw,kf. (511)

34 (micro-aggress* or microaggress*).tw,kf. (672)

35 incivil*.tw,kf. (735)

36 uncivil*.tw,kf. (228)

37 rude*.tw,kf. (1347)

38 mistreat*.tw,kf. (3323)

39 (professional* adj3 misconduct).tw,kf. (367)

40 mobbing.tw,kf. (536)

41 (negative behavio* or negative act?).tw,kf. (2165)

42 hazing.tw,kf. (104)

43 (gaslight* or malic*).tw,kf. (10258)

44 (Hidden adj5 (aggressi* or abus* or violenc*)).tw,kf. (257)

45 or/18-44 [Unprofessional behaviours] (63710)

46 exp health care personnel/ (1900535)

47 exp health student/ (135620)

48 exp medical education/ (375149)

49 public relations/ (63614)

50 workplace/ (50638)

51 teamwork/ (20829)

52 (nurs* or midwif* or midwiv*).tw,kf. (637750)

53 paramedic?.tw,kf. (9666)

54 (doctor? or physician? or clinician? or surgeon? or consultant?).tw,kf. (1549923)

55 (student? adj2 (medic* or health* or clinic*)).tw,kf. (93266)

56 intern?.tw,kf. (16967)

57 resident?.tw,kf. (250684)

58 (Therapist? or Pharmacist? or Optometrist? or Nutritionist? or Dentist? or Physiotherapist?).tw,kf. (226034)

59 (Audiologist? or Anatomist? or Allergist? or An?esthetist? or An?esthesiologist? or Cardiologist? or Dieti#ian? or Endocrinologist? or Gastroenterologist? or GP? or Geriatrician? or Hospitalist? or Oncologist?).tw,kf. (376178)

60 (Ophthalmologist? or Otolaryngologist? or Pathologist? or P?ediatrician? or Physiatrist? or Psychiatrist? or Pulmonologist? or Radiographer? or Radiologist?).tw,kf. (291043)

61 medic?.tw,kf. (42867)

62 assistant?.tw,kf. (43633)

63 (cleaner? or ancillary or porter?).tw,kf. (36354)

64 (auxillary or auxillaries or administrator? or secretary or secretaries or receptionist? or technician?).tw,kf. (58834)

65 (employee? or worker? or staff or personnel or practitioner? or professional? or workforce* or team*).tw,kf. /freq=2 (600934)

66 (workplace* or "work place*" or worksite* or "work site*" or "work setting*").tw,kf. (73577)

67 or/46-66 [Staff] (4249002)

68 45 and 67 [UB and Staff search 1] (18910)

69 public relations/ and (aggression/ or aggressiveness/ or prejudice/ or exp social discrimination/ or coercion/) (419)

70 ((staff or employee* or work* or nurs* or doctor?) adj8 (WPV or violen*) adj5 among*).tw,kf. (649)

71 ((staff or employee* or work* or nurs* or doctor?) adj8 Victim* adj5 among*).tw,kf. (142)

72 ((staff or employee* or work* or nurs* or doctor?) adj8 (humiliat* or hostil*)).tw,kf. (765)

73 ((staff or employee* or work* or nurs* or doctor?) adj3 undermin*).tw,kf. (389)

74 ((staff or employee* or work* or nurs* or doctor?) adj6 (discrimination or discriminatory or discriminated)).tw,kf. (3096)

75 "abusive supervision".tw,kf. (128)

76 (workplace adj3 (conflict* or aggressi* or abus*)).tw,kf. (668)

77 ((staff or employee* or work* or nurs* or doctor?) adj5 (gender adj2 (inequalit* or equalit*))).tw,kf. (207)

78 ((staff or employee* or work* or nurs* or doctor?) adj5 (racism or racist or (racial adj3 abus*))).tw,kf. (421)

79 ((staff or employee* or work* or nurs* or doctor?) adj5 (sexism or sexist)).tw,kf. (83)

80 ((staff or employee* or work* or nurs* or doctor?) adj8 ((disabilit* or disabled) adj5 (inequalit* or equalit*))).tw,kf. (24)

81 ((staff or employee* or work* or nurs* or doctor?) adj8 ableis*).tw,kf. (15)

82 ((staff or employee* or work* or nurs* or doctor?) adj8 coerci* adj5 among*).tw,kf. (12)

83 ((staff or employee* or nurs* or doctor?) adj1 (aggressi* or abus*)).tw,kf. (282)

84 or/69-83 [UB and staff search 2] (7035)

85 68 or 84 [UB among Staff final search] (24790)

86 17 and 85 [UB among Staff in Acute Care] (1767)

87 afghanistan/ or africa/ or "africa south of the sahara"/ or albania/ or algeria/ or andorra/ or angola/ or argentina/ or "antigua and barbuda"/ or armenia/ or exp azerbaijan/ or bahamas/ or bahrain/ or bangladesh/ or barbados/ or belarus/ or belize/ or benin/ or bhutan/ or bolivia/ or borneo/ or exp "bosnia and herzegovina"/ or botswana/ or exp brazil/ or brunei darussalam/ or bulgaria/ or burkina faso/ or burundi/ or cambodia/ or cameroon/ or cape verde/ or central africa/ or central african republic/ or chad/ or exp china/ or comoros/ or congo/ or cook islands/ or coted'ivoire/ or croatia/ or cuba/ or cyprus/ or democratic republic congo/ or djibouti/ or dominica/ or dominican republic/ or ecuador/ or el salvador/ or egypt/ or equatorial guinea/ or eritrea/ or eswatini/ or ethiopia/ or exp "federated states of micronesia"/ or fiji/ or gabon/ or gambia/ or exp "georgia (republic)"/ or ghana/ or grenada/ or guatemala/ or guinea/ or guinea-bissau/ or guyana/ or haiti/ or honduras/ or exp india/ or exp indonesia/ or iran/ or exp iraq/ or jamaica/ or jordan/ or kazakhstan/ or kenya/ or kiribati/ or kosovo/ or kuwait/ or kyrgyzstan/ or laos/ or lebanon/ or liechtenstein/ or lesotho/ or liberia/ or libyan arab jamahiriya/ or madagascar/ or malawi/ or exp malaysia/ or maldives/ or mali/ or malta/ or mauritania/ or mauritius/ or melanesia/ or moldova/ or monaco/ or mongolia/ or "montenegro (republic)"/ or morocco/ or mozambique/ or myanmar/ or namibia/ or nauru/ or nepal/ or nicaragua/ or niger/ or nigeria/ or niue/ or north africa/ or oman/ or exp pakistan/ or palau/ or palestine/ or panama/ or papua new guinea/ or paraguay/ or peru/ or philippines/ or polynesia/ or qatar/ or "republic of north macedonia"/ or romania/ or exp russian federation/ or rwanda/ or sahel/ or "saint kitts and nevis"/ or "saint lucia"/ or "saint vincent and the grenadines"/ or saudi arabia/ or senegal/ or exp serbia/ or seychelles/ or sierra leone/ or singapore/ or "sao tome and principe"/ or solomon islands/ or exp somalia/ or south africa/ or south asia/ or south sudan/ or exp southeast asia/ or sri lanka/ or sudan/ or suriname/ or syrian arab republic/ or taiwan/ or tajikistan/ or tanzania/ or thailand/ or timor-leste/ or togo/ or tonga/ or "trinidad andtobago"/ or tunisia/ or turkmenistan/ or tuvalu/ or uganda/ or exp ukraine/ or exp united arab emirates/ or uruguay/ or exp uzbekistan/ or vanuatu/ or venezuela/ or viet nam/ or western sahara/ or yemen/ or zambia/ or zimbabwe/ (1644602)

88 "organisation for economic co-operation and development"/ (2158)

89 exp australia/ or "australia and new zealand"/ or austria/ or baltic states/ or exp belgium/ or exp canada/ or chile/ or colombia/ or costa rica/ or czech republic/ or denmark/ or estonia/ or europe/ or exp finland/ or exp france/ or exp germany/ or greece/ or hungary/ or iceland/ or ireland/ or israel/ or exp italy/ or japan/ or korea/ or latvia/ or lithuania/ or luxembourg/ or exp mexico/ or netherlands/ or new zealand/ or north america/ or exp norway/ or poland/ or exp portugal/ or scandinavia/ or sweden/ or slovakia/ or slovenia/ or south korea/ or exp spain/ or switzerland/ or exp united kingdom/ or "turkey (republic)"/ or exp united states/ or western europe/ (3872230)

90 european union/ (30020)

91 developed country/ (35321)

92 or/88-91 (3904207)

93 87 not 92 (1492561)

94 86 not 93 [non OECD countries removed] (1595)

95 exp juvenile/ not exp adult/ (2786964)

96 94 not 95 [Child studies removed] (1443)

97 (elder mistreat* or elder abuse* or elder neglect*).tw,kf. (2330)

98 elder abuse/ (1733)

99 97 or 98 (2801)

100 96 not 99 [Elder abuse studies removed] (1398)

101 exp united states/ (1419795)

102 (USA or "united states" or america*).ti,ab,in,kf. (11644462)

103 101 or 102 (12262076)

104 100 and 103 (585)

Ovid MEDLINE(R) ALL 1946 to August 24, 2022

Date searched: 25-08-2022

Records found: 316

1 emergency medical services/ or advanced trauma life support care/ or call centers/ or emergency medical dispatch/ or emergency medical service communication systems/ (48547)

2 exp airway management/ or exp emergency treatment/ or exp ambulatory care/ or exp critical care/ or exp perioperative care/ or exp preoperative care/ (463588)

3 exp emergency service, hospital/ or emergency services, psychiatric/ or hotlines/ or poison control centers/ or exp "transportation of patients"/ or triage/ or critical care nursing/ or emergency nursing/ (133395)

4 (emergenc* adj5 (care or service* or health* or ill* or treat* or medic* or unit* or centre* or centre* or department* or setting*)).tw,kf. (211268)

5 (acute* adj5 (care or service* or health* or ill* or treat* or medic* or unit* or centre* or centre* or department* or setting* or ward?)).tw,kf. (228051)

6 (trauma* adj5 (care or service* or ill* or unit* or centre* or centre* or department*)).tw,kf. (27944)

7 (ambula* adj5 (care or service* or unit* or centre* or centre* or department* or setting*)).tw,kf. (26953)

8 (critical* adj2 (care or ill*)).tw,kf. (97288)

9 (urgent adj3 (care or service* or medic*)).tw,kf. (5676)

10 "intensive care".tw,kf. (180297)

11 paramedic*.tw,kf. (9528)

12 or/1-11 [Acute Care or Ambulance services] (1096671)

13 agonistic behavior/ (1788)

14 exp bullying/ (6143)

15 problem behavior/ (3530)

16 exp harassment, non-sexual/ (6227)

17 sexual harassment/ (2129)

18 incivility/ (263)

19 Professional Misconduct/ (3450)

20 Hostility/ (5459)

21 bully*.tw,kf. (7551)

22 harass*.tw,kf. (4951)

23 intimidat*.tw,kf. (1564)

24 (lateral* adj2 violence).tw,kf. (107)

25 (horizontal* adj2 violence).tw,kf. (147)

26 (transgressive adj3 behavio?r*).tw,kf. (41)

27 (disruptive adj3 behavio?r*).tw,kf. (4505)

28 (unprofessional adj3 behavio?r*).tw,kf. (424)

29 (micro-aggress* or microaggress*).tw,kf. (572)

30 incivil*.tw,kf. (734)

31 uncivil*.tw,kf. (226)

32 rude*.tw,kf. (1234)

33 mistreat*.tw,kf. (2612)

34 (professional* adj3 misconduct).tw,kf. (308)

35 mobbing.tw,kf. (426)

36 hazing.tw,kf. (91)

37 (negative behavio* or negative act?).tw,kf. (1789)

38 (gaslight* or malic*).tw,kf. (8741)

39 (Hidden adj5 (aggressi* or abus* or violenc*)).tw,kf. (205)

40 or/13-39 [Unprofessional behaviours] (49573)

41 exp Health Personnel/ (589916)

42 exp Students, Health Occupations/ (83031)

43 exp education, graduate/ or "internship and residency"/ or teaching rounds/ (96785)

44 exp Interprofessional Relations/ (72096)

45 exp Patient Care Team/ (72269)

46 Workplace/ (28057)

47 (nurs* or midwif* or midwiv*).tw,kf. (530620)

48 paramedic?.tw,kf. (6351)

49 (doctor? or physician? or clinician? or surgeon? or consultant?).tw,kf. (1040260)

50 (student? adj2 (medic* or health* or clinic*)).tw,kf. (69323)

51 intern?.tw,kf. (11676)

52 resident?.tw,kf. (187085)

53 (Therapist? or Pharmacist? or Optometrist? or Nutritionist? or Dentist? or Physiotherapist?).tw,kf. (137588)

54 (Audiologist? or Anatomist? or Allergist? or An?esthetist? or An?esthesiologist? or Cardiologist? or Dieti#ian? or Endocrinologist? or Gastroenterologist? or GP? or Geriatrician? or Hospitalist? or Oncologist?).tw,kf. (243485)

55 (Ophthalmologist? or Otolaryngologist? or Pathologist? or P?ediatrician? or Physiatrist? or Psychiatrist? or Pulmonologist? or Radiographer? or Radiologist?).tw,kf. (176891)

56 medic?.tw,kf. (22425)

57 assistant?.tw,kf. (29950)

58 (cleaner? or ancillary or porter?).tw,kf. (25972)

59 (auxillary or auxillaries or administrator? or secretary or secretaries or receptionist? or technician?).tw,kf. (42828)

60 (employee? or worker? or Staff or personnel or practitioner? or professional? or workforce* or team*).tw,kf. /freq=2 (434686)

61 (workplace* or "work place*" or worksite* or "work site*" or "work setting*").tw,kf. (58882)

62 or/41-61 [Staff] (2786785)

63 40 and 62 [UB and Staff search 1] (13814)

64 physician-nurse relations/ or interprofessional relations/ (55150)

65 aggression/ or prejudice/ or Social Discrimination/ or ageism/ or gender equity/ or homophobia/ or exp racism/ or sexism/ or weight prejudice/ or xenophobia/ or Perceived Discrimination/ or Coercion/ (77044)

66 64 and 65 [Aggression or prejudice among staff MeSH] (767)

67 ((staff or employee* or work* or nurs* or doctor?) adj8 (WPV or violen*) adj5 among*).tw,kf. (610)

68 ((staff or employee* or work* or nurs* or doctor?) adj8 coerci* adj5 among*).tw,kf. (13)

69 ((staff or employee* or work* or nurs* or doctor?) adj8 Victim* adj5 among*).tw,kf. (123)

70 ((staff or employee* or work* or nurs* or doctor?) adj8 (humiliat* or hostil*)).tw,kf. (593)

71 ((staff or employee* or work* or nurs* or doctor?) adj3 undermin*).tw,kf. (362)

72 ((staff or employee* or work* or nurs* or doctor?) adj6 (discrimination or discriminatory or discriminated)).tw,kf. (2672)

73 "abusive supervision".tw,kf. (159)

74 (workplace adj3 (conflict* or aggressi* or abus*)).tw,kf. (598)

75 ((staff or employee* or work* or nurs* or doctor?) adj5 (gender adj2 (inequalit* or equalit*))).tw,kf. (231)

76 ((staff or employee* or work* or nurs* or doctor?) adj5 (racism or racist or (racial adj3 abus*))).tw,kf. (390)

77 ((staff or employee* or work* or nurs* or doctor?) adj5 (sexism or sexist)).tw,kf. (91)

78 ((staff or employee* or work* or nurs* or doctor?) adj8 ((disabilit* or disabled) adj5 (inequalit* or equalit*))).tw,kf. (18)

79 ((staff or employee* or work* or nurs* or doctor?) adj8 ableis*).tw,kf. (9)

80 ((staff or employee* or nurs* or doctor?) adj1 (aggressi* or abus*)).tw,kf. (242)

81 or/66-80 [UB and staff search 2] (6606)

82 63 or 81 [UB among Staff final search] (19495)

83 12 and 82 [UB among Staff in Acute Care] (1074)

84 afghanistan/ or africa/ or africa, northern/ or africa, central/ or africa, eastern/ or "africa south of the sahara"/ or africa, southern/ or africa, western/ or albania/ or algeria/ or andorra/ or angola/ or "antigua and barbuda"/ or argentina/ or armenia/ or azerbaijan/ or bahamas/ or bahrain/ or bangladesh/ or barbados/ or belize/ or benin/ or bhutan/ or bolivia/ or borneo/ or "bosnia and herzegovina"/ or botswana/ or brazil/ or brunei/ or bulgaria/ or burkina faso/ or burundi/ or cabo verde/ or cambodia/ or cameroon/ or central african republic/ or chad/ or exp china/ or comoros/ or congo/ or cote d'ivoire/ or croatia/ or cuba/ or "democratic republic of the congo"/ or cyprus/ or djibouti/ or dominica/ or dominican republic/ or ecuador/ or egypt/ or el salvador/ or equatorial guinea/ or eritrea/ or eswatini/ or ethiopia/ or fiji/ or gabon/ or gambia/ or "georgia (republic)"/ or ghana/ or grenada/ or guatemala/ or guinea/ or guinea-bissau/ or guyana/ or haiti/ or honduras/ or independent state of samoa/ or exp india/ or indian ocean islands/ or indochina/ or indonesia/ or iran/ or iraq/ or jamaica/ or jordan/ or kazakhstan/ or kenya/ or kosovo/ or kuwait/ or kyrgyzstan/ or laos/ or lebanon/ or liechtenstein/ or lesotho/ or liberia/ or libya/ or madagascar/ or malaysia/ or malawi/ or mali/ or malta/ or mauritania/ or mauritius/ or mekong valley/ or melanesia/ or micronesia/ or monaco/ or mongolia/ or montenegro/ or morocco/ or mozambique/ or myanmar/ or namibia/ or nepal/ or nicaragua/ or niger/ or nigeria/ or oman/ or pakistan/ or palau/ or exp panama/ or papua new guinea/ or paraguay/ or peru/ or philippines/ or qatar/ or "republic of belarus"/ or "republic of north macedonia"/ or romania/ or exp russia/ or rwanda/ or "saint kitts and nevis"/ or saint lucia/ or "saint vincent and the grenadines"/ or "sao tome and principe"/ or saudi arabia/ or serbia/ or sierra leone/ or senegal/ or seychelles/ or singapore/ or somalia/ or south africa/ or south sudan/ or sri lanka/ or sudan/ or suriname/ or syria/ or taiwan/ or tajikistan/ or tanzania/ or thailand/ or timor-leste/ or togo/ or tonga/ or "trinidad and tobago"/ or tunisia/ or turkmenistan/ or uganda/ or ukraine/ or united arab emirates/ or uruguay/ or uzbekistan/ or vanuatu/ or venezuela/ or vietnam/ or west indies/ or yemen/ or zambia/ or zimbabwe/ (1240847)

85 "Organisation for Economic Co-Operation and Development"/ (464)

86 australasia/ or exp australia/ or austria/ or baltic states/ or belgium/ or exp canada/ or chile/ or colombia/ or costa rica/ or czech republic/ or exp denmark/ or estonia/ or europe/ or finland/ or exp france/ or exp germany/ or greece/ or hungary/ or iceland/ or ireland/ or israel/ or exp italy/ or exp japan/ or korea/ or latvia/ or lithuania/ or luxembourg/ or mexico/ or netherlands/ or new zealand/ or north america/ or exp norway/ or poland/ or portugal/ or exp "republic of korea"/ or "scandinavian and nordic countries"/ or slovakia/ or slovenia/ or spain/ or sweden/ or switzerland/ or turkey/ or exp united kingdom/ or exp united states/ (3432805)

87 European Union/ (17334)

88 Developed Countries/ (21208)

89 or/85-88 (3448340)

90 84 not 89 [OECD search filter NICE 2021] (1152820)

91 83 not 90 [UB among Acute Care Staff with non-OECD countries removed] (977)

92 (exp Child/ or Adolescent/ or exp Infant/) not exp Adult/ (2073393)

93 91 not 92 [Child studies removed] (902)

94 (elder mistreat* or elder abuse* or elder neglect*).tw,kf. (1932)

95 93 not 94 [Elder abuse studies removed] (878)

96 exp united states/ (1440539)

97 (USA or "united states" or america*).ti,ab,in,kf. (5886737)

98 96 or 97 (6652358)

99 95 and 98 (316)

Google Scholar (Via Harzing’s Publish or Perish)

Date searched: 25-08-2022

Records downloaded: 14

Search 1. 14 records found

bullying | harassment | discrimination|unprofessional AND workplace|worker|staff AND model|framework|concept|idea|opinion|theory|view|perception|attitude|theories [Searched in Publish or Perish Title Field]

USA | america | united states [KEywords field]

Years limited to 2022-2022

Search 2. 0 records found

interventions|strategies|techniques|program|programs|programme|programmes AND unprofessional|bullying|harassment|discrimination AND nurse|doctor|paramedic|hospital|ambulance|staff|professional AND emergency|acute|trauma [searched in Publish or Perish Keywords field]

USA | america | united states [KEywords field]

Years limited to 2022-2022

Patient Safety Network <https://psnet.ahrq.gov/>

Date searched: 25-08-2022

Records found: 1

abuse bullying unprofessional harassment discrimination microaggression incivility intimidation humiliation hostility mobbing hazing gaslighting malicious

Limited to Ambulatory Care setting OR Limit to Hospital setting. Added in last 6 months
