# Supplementary material for: Interventions to address unprofessional behaviours between staff in acute care: what works for whom and why? A realist review
Source: BMC Med. 2023 Oct 31;21:403. doi: 10.1186/s12916-023-03102-3 (PMC10617100; doi:10.1186/s12916-023-03102-3)
Supplement: Supplementary file 3 — Additional file 3. Table of intervention characteristics. [file 12916_2023_3102_MOESM3_ESM.docx]

# Additional File 3. Table of intervention characteristics.

| **Study** | **Sample*** | **Duration** | **Design and data collection timepoints** | **Theoretical framework** | **Strategies** | **Measures** | **Findings** | **Reported effective by study authors (Y, N or n/a)** |
| --- | --- | --- | --- | --- | --- | --- | --- | --- |
| Single-session education/training | | | | | | | | |
| Ceravolo et al. (2012)[91] | 4032 practicing nurses, 1160 students and faculty | 60-90 minute workshops over 3 years | Quasi-experimental pre-post design without control. Pre (2007) and post 3-year data collection via survey (2011). | None | Education / awareness (assertiveness training), role modelling | Verbal Abuse Survey | This intervention used culture-change and communication enhancing workshops to decrease lateral violence in a five-hospital integrated health and care system. Nursing turnover and vacancy rates decreased but it was not clear if that was due to the intervention. | Y |
| Clark, Ahten and Macy (2013)[146] | 65 senior nursing students | 70 minute session | Pre-post design without control.  Only post-measures were delivered, immediately post-session. | Unknown | Problem-based learning, role playing | Custom feasibility questionnaire | Study of an intervention which used PBL to address incivility. It was found to heighten awareness of incivility, improve favourable reaction to PBL, and give increased confidence. | Y |
| Dahlby and Herrick (2014)[178] | 25 nurses on two nursing units. | 1.5 hour session | Pre-post design without control. Posttest timing is unclear. | None | Education, cognitive rehearsal | Lateral and Vertical Violence in Nursing Survey | A study examining the effects of a 1.5 hour educational intervention on lateral violence. They found participants were better able to identify causes of lateral violence and it perhaps contributed to reducing frequency too. However, no statistically significant results were noted other than for lateral violence becoming less serious post-intervention. | Y |
| Embree, Bruner and White (2013)[140] | 143 nurses | Two-hour session | Quasi-experimental pre-post design without control. Some post-measures were immediate and others such as internalised sexism were one-year later. | None | Education, cognitive rehearsal | Internalized Sexism, Minimization of Self, Total Nurse Workplace Behavior Scale, and Silencing the Self-Work Scale, RN voluntary turnover | Investigates effectiveness of a cognitive rehearsal education intervention on nurse to nurse lateral violence. It found that there was a trend towards increased awareness of lateral violence but no statistically significant results were identified. | N |
| Griffin (2004)[76] | 26 newly-enrolled nurses | Two-hour session | Quasi-experimental pre-post design without control. Data were collected post-only, occurring one-year post-intervention. | Cognitive learning theories | Cognitive rehearsal | Focus groups | Investigates a cognitive rehearsal intervention as a shield for lateral violence in newly licensed nurses. It finds that knowledge of lateral violence enabled greater coping and confronting skills. Retention rate improved. | Y |
| Griffith et al. (2019)[114] | 25 participants | One session of unknown length | Quasi-experimental post-only design without control. Data collection was only post-intervention via survey over an unclear timeline. | Six-step approach to curricular development by Kern et al. | Education, action planning, keeping records | Custom electronic evaluation of effectiveness | The authors developed an educational advance programme to aid residents and faculty in understanding and improving their learning environment. Attendees proposed coaching, signage, zero tolerance policies, and more, to tackle mistreatment. There was some evidence it increased awareness. | Y |
| Hawkins et al. (2022)[166] | 111 nurses across 12 units in four acute care hospitals | One session | Quasi-experimental pre-post design with control group (non-randomised). Baseline data collection occurred over the month prior to the intervention and post-data collection occurred over 6 months. | None | Education | Negative Acts Questionnaire— Revised, Ways of Coping Questionnaire | The intervention group experienced less of a reduction in bullying and negative behaviours than the control group. But no results were statistically significant. Authors recommended against single-session attempts to reduce UB. | N |
| Kile et al. (2019)[98] | 19 nurses | 2-hour training session | Pilot study with no control group with pre, post (immediate), and 6 week follow-up | Bandura’s social learning theory (1977) | Education, cognitive rehearsal | Adapted versions of the nursing Incivility Survey (NIS), the Nurse Interaction subscale of the National Database of Nursing Quality Indicators (NDNQI), and a questionnaire with two open‐ended questions | Investigated with a mixed methods pilot study the impact of incivility and cognitive rehearsal education on nurse-to-nurse incivility. They found that it was effective at increasing recognition and confronting of incivility due to movement in means on the NIS subscales, and reduced perceived incivility. | Y |
| Nikstatis and Simko (2014)[94] | 21 nurses | 1-hour session | Pilot study pre-post design without control group. Both pre- and post-data collection occurred over a 3-week timeframe. | None | Education, group discussion | Nursing incivility scale | A quantitative pilot study using a 1-group pre and post intervention test design to assess a 60 minute educational programme. They found that it increased perceived incivility. However this was not statistically significant. | N |
| O’Connell, Garbark and Nader (2019)[92] | 76 participants | 2-hour session | A pre-post study design with no control group. | None | Education, cognitive rehearsal, role play | Negative Acts Questionnaire - Revised (NAQ-R) | A quantitative exploration of nurses’ perceptions of lateral violence within a military setting and the impact of an education, cognitive rehearsal, and role play intervention. Six negative acts occurred daily or weekly preintervention and nine occurred postintervention. Putting together staff on different hierarchy levels into one session may have undermined results. | N |
| Stagg et al. (2017)[89] | 10 nurses | 2-hour session | Pilot study with post-only testing (6-months after the intervention over a 6-week period) without control. | None – based on Griffin (2004) | Education, cognitive rehearsal | Workplace Bullying Follow-Up Survey | Assessed the effectiveness of a 2-hour cognitive rehearsal programme, 6 months after completion. 70% of nurses reported changing behaviours, and 40% reported a decrease in bullying behaviours. | Y |
| Stagg et al. (2011)[144] | 20 nurses | 2-hour session | Pre-post quasi-experimental design with no control group. Unclear when post-test was delivered. | None – based on Griffin (2004) | Education, cognitive rehearsal | Adapted Workplace Bullying Inventory | Evaluated a workplace bullying cognitive rehearsal programme. Significant differences were found in the results between pre-test and post-test for observed bullying (Z = -2.636, p < .01), bullying of others (Z = -2.449, p < .05), and sufficiency of the training on management of bullying (Z = -3.358, p < .01). | Y |
| Warrner et al. (2016)[79] | 60-bed orthopaedic inpatient unit incl. management | 45-minute session | Pre-post design without control group, with follow-up at 2-months post-intervention. | None | Education, cognitive rehearsal, environmental modification | Nursing Incivility Scale (NIS) | Evaluates an intervention comprising awareness education, cognitive rehearsal, and which included management. None of the scores for the five subscales of sources of incivility were statistically significant. Two out of five subscales showed a statistically significant decrease in instances of perceived incivility: general incivility (2.75 to 2.24, p = 0.00) and physician incivility (2.79 to 2.43, p = 0.04), and the others decreased but not significantly. | Y |
| Multiple session education/training | | | | | | | | |
| Asi Karakaş and Okanli (2015)[147] | 30 nurses | Eight 2-2.5 hour sessions | Quasi-experimental pre-post design without control. Assessment was 6-months post-intervention. | None | Assertiveness training | Mobbing Scale, Rathus Assertiveness Inventory | This study reported an evaluation of an assertiveness training intervention in 30 nurses who experienced a high level of mobbing. Results indicated a statistically significant fall in mobbing after the intervention from 226.4 +- 27.7 to 159.6 +- 47.9 on the mobbing scale and an increase in assertiveness from 6.23 +- 15.6 to 17.0 +- 16.06. | Y |
| Banerjee et al. (2022)[85] | Division faculty members (n = 41) and pulmonary and critical care fellows (n = 12) | 13x 1-hour sessions over one year | Pre-post feasibility study. Time of post-assessment is unclear but likely immediately post-intervention. | None | Education, positive role-modelling | Surveys assessed knowledge on racism in medicine; opinions, understanding, and comfort surrounding race and racism in medicine; as well as additional questions to solicit feedback on the curriculum itself | Assessed the feasibility of a year-long antiracism educational study. As it was mostly a feasibility study, postintervention surveying indicated a 15% increase in self-directed learning on related topics. However, interest in the curriculum actually decreased post-intervention by 14%, perhaps since participants now felt they had learned what they needed to. | n/a |
| Barrett et al. (2009)[128] | An inpatient unit, critical care unit, emergency department, and inpatient operating room  59 pre-intervention and 45 post-intervention nurses | Two 2-hour group sessions | Quasi-experimental pre-post design without control. Pre-intervention measures were 2-weeks prior to intervention and post-measures were 3-months after intervention. | None | Education and role playing, encouragement to become champions, teambuilding, seeking hospital accreditation | National Database of Nursing Quality Indicators (NDNQI) RN-RN interaction subscale, Group Cohesion Scale | The study assessed a team-building intervention to reduce lateral violence, using mixed methods. The intervention was found to improve group cohesion from 540 pts to 612 pts (p=0.037). | Y |
| Demarco, Roberts, and Chandler (2005)[176] | 5 graduate nursing student participants | 2 hours per week for 6 weeks | Pilot pre-post design without control. Interviews were as soon as possible post-intervention. | Oppressed group behaviour (Freire, 1990) | Group writing | Interview | Pilot study investigating a writing group’s ability to build group cohesion. The group writing drew on the ”Amherst Writers and Artists” method which did not focus on UB but nonetheless was intended to help cope with it. It explored participants’ responses qualitatively, finding it helped build a sense of social support, and recommended potential changes. No quantitative effectiveness testing. | Y |
| Jenkins et al. (2011)[132] | 10 student leaders | 6-hour long sessions, monthly, for 6 months | Case study design | Unknown | Journal club / group writing | Weekly journal comments | Explores how 6 monthly hour-long journal club meetings may increase civility. Also incorporated journalling. Anecdotally, it found that participants became more aware and sought to become role models of civility. | n/a |
| Kang, Kim and Yun (2017)[97] | 40 hospital nurses | 20-hours over 10 sessions | Randomised controlled trial | 4 stages of cognitive rehearsal by Smith and non-violence communication (Rosenberg & Chopra, 2015) | Cognitive rehearsal, role playing, and communication training | Relationship Change Scale, Negative Acts Questionnaire-Revised, Brief Symptom Inventory-18, Yun’s nurse turn-  over intention tool | An RCT to investigate a cognitive rehearsal programme on workplace bullying. Post-intervention, there were significant differences in interpersonal relationships (F=6.21, p=.022) and turnover intention (F=5.55, p=.024) between groups, but not for workplace bullying. | Y |
| Kousha et al. (2022)[157] | 80 emergency nurses | Five 2-hour sessions over 3 weeks | Single-blinded randomised controlled trial with two hospitals as groups (education-only vs. education and cognitive rehearsal). Post-collection of data was 1-month after training sessions. | Bandura’s social learning theory (1977) | Cognitive rehearsal, education, role-play | The Incivility Scale | The study, focused on incivility between emergency nurses, found that ANOVA and repeated measures ANOVA indicated overall incivility and supervisor incivility increased in the intervention group but decreased in the control group. There were no notable differences in instances of nurse-to-nurse incivility and physician incivility. | N |
| Lasater et al. (2015)[109] | 94 nursing staff | One 1-hour session comprising presentation and discussion on incivility, a 4-hour session on norm-setting and action planning, and a 2-hour simulation role playing session | Quasi-experimental study with no control group. Data collection occurred pre, post-session 1, post-session 2, post-session 3, >1 month post-session 3, 5 months after the last session, and 24-months after the last session. | Bandura’s social learning theory (1977) | Education, action planning, role playing | Nurse Incivility Scale (NIS)  New General Self-Effectiveness Scale (NGSE), Workplace Collective Effectiveness Scale (WCES),  National Database for Nursing Quality Indicators (NDNQI) | Study of a three-part educational intervention to see if it reduced incivility in two units of a hospital. It was effective in decreasing incidences of perceived incivility across all NIS subscales and self-effectiveness increased significantly. | Y |
| Nicotera, Magon and Wright (2014)[159] | 19 participants with 47 comparison sample | 6x 90 minute sessions | Pilot quasi-experimental pre-post design with comparison group. No randomisation. Post data collection was unclear. | Structurational divergence | Education, conflict management, role playing | Structurational divergence, role conflict, burnout, depression, bullying, beliefs about arguing, verbal aggressiveness, taking conflict personally (TCP, a set of attitudes predisposing one to internalize conflict), ambiguity intolerance, and conflict management styles (collaborative, avoidant, and aggressive), general conflict orientations, scales for direct constructive discussion and direct destructive discussion | Assesses a nursing conflict intervention using mixed pre and post-test methods with a control sample, which sought to reduce structurational divergence by raising awareness of it and teaching skills to manage it. They found that participants experienced better understanding of conflicts and how to sustain healthier relationships. The course significantly reduced destructive communication and improved constructive communication skills. | Y |
| O’Keefe, Brennan and Doherty (2022)[103] | 203 participants in surgery | 1-day session with a 1-hour follow up e-learning course | Case study | Transformative learning theory conceptual framework | Role-playing, team-building, education, reflection | Online qualitative survey | Outlines an intervention based on a Transformative Learning Theory conceptual framework that draws upon role playing, simulations, case studies, reflection exercises, and peer-peer learning. Anecdotally, 85% of participants reported a perceived improvement in conflict management skills but effectiveness was not formally tested. | n/a |
| Saxton (2012)[181] | 17 participants | Two-day programme | Pre-post study design without control group. Post-intervention data were collected immediately. Data collection also at 4-week follow-up. | Humanizing Nursing Communication Theory and Social Cognitive Theory | Communication training | Self-Effectiveness to Address Disruptive Behavior Scale | Development and evaluation of a communication skills intervention to improve perceived self-effectiveness of perioperative nurses using a pre-post design. Results suggested an improvement in self-effectiveness scores and participants reported the ability to address disruptive physician behaviour 71% of the time four weeks after the study intervention. | Y |
| Training and/or further actions (e.g. codes of conduct) | | | | | | | | |
| Chipps and McRury (2012)[96] | 16 staff members | 3 month | Quasi-experimental pre-post design without control with 4-month follow-up. Posttest given immediately post-intervention. | Einarsen’s (2000) theoretical framework on predisposing factors for workplace bullying | Education, peer learning, action planning, conflict management training, code of conduct, communication training | Negative Acts Questionnaire-Revised (NAQ-R). Log books given for daily documenting of bullying experiences. | This quasi-experimental study assessed pre-test and post-test an educational intervention to address workplace bullying. They noted an increase in bullying reports as a result (from <1 act weekly/daily to 1.6 acts weekly/daily), but it did help develop a learning community. | N |
| Dimarino (2011)[168] | Unknown | Unknown – ‘on demand’ sessions and code of conduct | Case study | None | Code of conduct, education | Reports anecdotally on organisational turnover and reports of violence. | Reporting of one ambulatory surgery centre’s approach to reducing lateral violence through education about lateral violence, and zero tolerance policies. Did not test effectiveness. | n/a |
| Kang and Jeong (2019)[86] | 72 hospital nurses | Two-hour familiarity session followed by 8-weeks on-demand usage (smartphone based) | Cluster quasi-randomised design with control group with pre, post (4-week post), and 8-week follow-up measurement | Non‐violent communication (Rosenberg & Chopra, 2015) | Cognitive rehearsal (smartphone based) | Negative Acts Questionnaire‐Revised, turnover intentions were measured using a modified version of “intent to quit” | Investigates a cognitive rehearsal smartphone-based intervention’s impact on bullying using a cluster quasi-randomised trial. It found that use of the app reduced nurses' person‐related (21.07+-8.38 to 15.41+-4.03 at 8 wks, ICU staff) and work‐related bullying experiences (11.19+-5.02 to 7.81+-3.20 at wks, ICU staff) and turnover intentions (3.52+-0.8 to 3.21+-0.71 at 8 wks, ICU staff) between groups, but did not reduce intimidation‐related bullying experiences. | Y |
| Parker et al. (2016)[119] | Unclear / organisation-wide | One away day & subsequent ongoing efforts of unclear overall duration | Case study design | Longo’s (2010) three-level intervention model: organization, leadership, and individual | Education, conflict management, leadership training, role modelling, cognitive rehearsal, code of conduct, championing / becoming agents of change, seeking Magnet status | Briles’ Sabotage Savvy Questionnaire | Explores how nurses at an acute care hospital were able to implement multiple interventions to reduce horizontal violence prevalence in the organisation. These included 13 total organisational, leadership, and individual level strategies. | n/a |
| Stevens (2002)[111] | Unclear | Unclear | Case study design | None | Action planning, code of conduct, democratisation | Turnover rate | Informally explores the impact of a multi-strategy anti-bullying intervention in a large teaching hospital, delivered mostly through workshops for education, policies, supervisor training, and more. They anecdotally found a decrease in nursing turnover rate after 1 year but it could have been also a result of other factors and programmes. | n/a |
| Thorsness and Sayers (1995)[95] | Approximately 100 surgical staff | Unclear | Pre-post design without control group. Survey was conducted before and 2.5 years after intervention. | Kilmann model for transforming organisations (1974) | Survey, code of conduct, action planning, communication skills training, workplace rearrangement, education | Kilmann-Saxton Culture-Gap Survey | Evaluation of a programme adopting a systems approach to cultivating a positive work environment for perioperative staff members. Intervention comprised making action plans for different staff groups, code of conduct, and cultural change efforts. Post-survey showed improvements in culture but statistical significance was not tested. | Y |
| Professional accountability and reporting | | | | | | | |  |
| Baldwin et al. (2022)[182] | Three academic medical centres | Sept 2019 to Aug 2021 (2 years). | Descriptive study assessing feasibility of implementation. | None | Vanderbilt intervention (reporting and escalation system, informal and formal resolution, championing, peer messengers, seeking Magnet accreditation) | Statistics from message database. | Descriptive study analysing the types of reports received during the intervention to promote professionalism with nurses. 590 reports were received, of which most included more than one problematic behaviour. 76.5% of these messages were shared and completed. | n/a |
| Churruca et al. (2022)[77] | Eight hospitals | 5 years | Case study / descriptive design. Reported data is from between July 2017 and July 2021. | Unknown | Ethos reporting system with peer messengers, informal resolution, formal investigation, training to enhance speaking up and role-modelling. | Reported statistics from reporting system such as percentage of positive vs. negative reports | Reports on a case study of the Ethos messaging system implemented across eight hospitals in Australia to address a culture of respect. The Messaging System had 2497 submissions with approximately 1600 reflection conversations delivered, 54% submissions were about positive behaviours. Peer messengers faced some difficulties when delivering messages. | n/a |
| Dixon-Woods et al. (2019)[125] | Organization-wide at Johns Hopkins Medicine | Two-year period, 2014-2016 | ‘Case study’ approach | None | Reporting system and formal investigation | Interviews. | This study sought to improve employee’s ability to speak up about transgressive and disruptive behaviour at a John Hopkins Medicine hospital. It uses largely qualitative exploration of the results and highlights importance of leader commitment and intolerant culture. Makes suggestions for a testable approach to encouraging voice. Did not test effectiveness. | n/a |
| Hickson et al. (2007)[104] | Unknown | Variable, depends on requirements | Case study design | Unknown | Vanderbilt approach for graduated intervention (reporting system, informal conversation, awareness, leader-led action planning, formal investigation) & other variable strategies as required (e.g. communication training, leadership programmes, use of messengers) | Reports statistics on reporting system use and types of complaint. | Outlines and reviews the Vanderbilt approach to identifying, measuring, and addressing UB using four graduated interventions. Did not assess effectiveness. | n/a |
| McKenzie et al. (2019)[163] | 21 healthcare staff pre-to-post | 18 months into a 3-year intervention | ‘Case study’ design | Unknown | Vanderbilt approach (education, reporting system, champions, action plans, graduated informal to formal resolution) | Interviews and Safety Attitudes Questionnaire (SAQ) | Investigated factors affecting implementation of a multistrategy intervention using education, reporting systems with graduated intervention processes, safety champions, and action plans, to tackle unprofessional behaviour. They found that leader role modelling, work condition modification, confidence in accountability systems, and responsiveness enhanced the intervention implementation. | n/a |
| Speck et al. (2014)[169] | Three teaching hospitals | 4+ years | Case study design | Unknown | Variation on the Vanderbilt reporting system with graduated escalation from informal resolution (peer, then manager) to formal investigation, championing | Reports on professionalism committee statistics and vignettes | Assessment of a professionalism committee approach to tackling unprofessional behaviour across three large teaching hospitals. In this variation, department chairs were able to report individuals to the committee rather than any staff member. It found that it was able to identify early specific behavioural issues and refer them appropriately. | n/a |
| Webb et al. (2016)[81] | Three hospitals | 2 years for study data (but programme running for 9 years) | Case study/ evaluation | Unknown | Vanderbilt reporting system with graduated escalation from informal resolution (peer, then manager) to formal investigation, championing | Reports on statistics from the reporting system such as number of reports and escalations | Presents a feasibility study of the Co-Worker Observation Reporting System implemented by Vanderbilt University Medical Centre to reduce disrespectful and unsafe behaviours. They found that it was feasible, requiring organisation-wide implementation and found that most workers self-regulate after being informed a report was received about them. | n/a |
| Westbrook et al. (2023)[129] | Staff across five hospitals | 2.5-3 years after Ethos implementation | Pre-post cross-sectional study. Hospitals were surveyed at baseline, between July-November 2018, prior to programme introduction and repeated between October 2021-February 2022 (post). | Unknown | Ethos reporting system, training for leaders and staff to support speaking up and to role-model, tiered accountability pathway. | Longitudinal Investigation Of Negative behaviour (LION) survey, follow up survey including questions about the perpetrators of unprofessional behaviours, knowledge and views of the Ethos program, and the impact of COVID-19 on unprofessional behaviours | There was an overall reduction in the odds of staff experiencing incivility/bullying behaviours by 24%, and a 32% reduction in odds of experiencing extreme unprofessional behaviours in the previous 12 months. | Y |
| Structured and Structured culture change interventions | | | | | | | | |
| Armstrong (2017)[130] | 9 nurses | Two 8-hour sessions to train facilitators  Four weeks total with one meeting per week. Sessions lasted 20-30 mins. | Pre-post design without control. Assessment two-weeks post-intervention. | Model for Improvement by Langley (1996) | CREW (Education, teambuilding exercises, roleplaying) | Workplace Incivility Scale, Confidence Scale | Quantitative investigation of an intervention in a rural hospital to reduce nursing workplace incivility. Findings indicated no change in experience of incivility but a greater ability to respond to incivility (there was a statistically significant improvement in the posttest mean score (M = 85.56, SD =20.07, t (8) = -4.667, p =.002), when compared to the pretest mean score (M = 62.22, SD = 18.56). | Y |
| Laschinger et al. (2012)[135] | 8 units with 33 controls | 6 months, selecting strategies from the CREW toolkit as appropriate | Quasi-experimental design using randomised units. Pre and post assessment. Post-assessment timing is unclear. | Kanter’s (1977, 1993) Theory of Structural Power in Organizations | CREW intervention (espousing values by leadership / role modelling, education, signing code of conduct pledge, surveying civility, action planning, role playing, training on assertiveness, communication, conflict resolution). | Structural empowerment was measured using four subscales of the Conditions for Work Effectiveness Questionnaire-II, five items from the Workplace Incivility Scale, Six items from Cook and Wall’s (1980) Interpersonal Trust at Work Scale | Investigates the impact of the CREW intervention over 6 months with 8 intervention units and 33 control units. A significant interaction of time by intervention was found for the access to support and resources empowerment structures, total empowerment, supervisor incivility, and trust in management | Y |
| Leiter et al. (2011)[173] | 1,173 workers across 41 units | 6-months | Quasi-experimental design using randomised units. Pre and post assessment. Post-assessment timing is unclear. | Social interactions at work | CREW intervention (espousing values by leadership / role modelling, education, signing code of conduct pledge, surveying civility, action planning, role playing, training on assertiveness, communication, conflict resolution, leadership). | CREW Civility Scale, The 10-  item Workplace Incivility Scale and  an additional dimension of instigated workplace incivility was included Blau and Andersson  (2005), respect was measured using two items from the  Esteem Reward section of the Effort-Reward Imbalance Questionnaire, trust in management was measured  by six items from Cook and Wall’s (1980) Interpersonal Trust at Work Scale, Emotional Exhaustion and Cynicism subscales  of the Maslach Burnout Inventory-General Survey, three items were modified from the  Turnover Intentions measure developed by Kelloway, Gottlieb, and Barham (1999), Professional Effectiveness was measured using the Professional Effectiveness scale of the MBI-GS, Two items from the Affective Commitment Scale (Allen & Meyer, 1990), job satisfaction (Hackman & Oldham, 1975; Tsui, Egan & O’Reilly, 1992), absenteeism from institutional data. | Assesses the effect of 6 months of the CREW intervention and found that greater group x time interactions were found in the intervention group for civility, supervisor incivility, respect, cynicism, job satisfaction, and management trust, and absences. | Y |
| Osatuke et al. (2009)[123] | 647 post-intervention CREW participants and 680 comparison (total 34 workgroups) | Flexible/various | Quasi-experimental pre-post design with control group but no randomisation. The Civility scale for CREW was  administered at CREW-1 sites in September 2005 and July 2006, and at CREW-2 sites in February 2006 and March 2007. Comparison site data were retrospectively matched to intervention sites for respective years. | National Center for Organization Development (NCOD) practice model. Prototype approach. | CREW intervention (survey, action planning, various training e.g., on communication, education) | CREW civility scale | Preliminary evaluation of a nationwide Veterans Health Administration interventions called CREW across 23 sites. It found significant pre to post-intervention changes in civility compared to none at comparison sites. | Y |
