# Supplementary material for: Interventions to address unprofessional behaviours between staff in acute care: what works for whom and why? A realist review
Source: BMC Med. 2023 Oct 31;21:403. doi: 10.1186/s12916-023-03102-3 (PMC10617100; doi:10.1186/s12916-023-03102-3)
Supplement: Supplementary file 4 — Additional file 4. Use of strategies in different intervention types. [file 12916_2023_3102_MOESM4_ESM.docx]

# Additional File 3. Use of strategies in different intervention types.

Further details on use of strategies in different intervention types:

- Single-session interventions drew predominately on awareness-raising strategies such as education about UB (n=2). In 11 cases, this was also combined with role-playing and other activities intended to enhance and practice the ability to speak up and challenge UB in the moment.
- Multi-session interventions typically incorporated similar strategies, but often diversified the approach to include strategies aimed at increasing the ability to speak up or improving the ability to work together, such as assertiveness training (n=1), problem-based learning (n=1), conflict management training (n=1) group writing or journalling (n=2) and communication training (n=1). However, most still drew on a mix of cognitive rehearsal/role playing and education (n=4).
- Combined sessions with other activity interventions often sought to firstly, have a training or education session to, for example, improve knowledge and awareness of UB and ability to speak up, alongside, and secondly, non-session activities such as the addition of a code of conduct (n=6).
- Professional accountability interventions often utilised a reporting and escalation system at their core. These interventions typically combined this with training to enhance speaking up and role-modelling by leadership in the case of Ethos [77, 129] or incorporated championing (i.e. encouraging individuals to role-model and espouse the benefits of the intervention) in the case of Vanderbilt interventions [81, 104, 125, 163, 169, 182].
- Structured culture change interventions tended to use a bespoke response to organisational needs including (1) action planning to assess which strategies to implement and (2) surveys to understand the initial landscape of UB. Strategies used within these interventions included training on assertiveness, communication, and conflict resolution, as well as training for leaders and other social support-building strategies.
