# Supplementary material for: Interventions to address unprofessional behaviours between staff in acute care: what works for whom and why? A realist review
Source: BMC Med. 2023 Oct 31;21:403. doi: 10.1186/s12916-023-03102-3 (PMC10617100; doi:10.1186/s12916-023-03102-3)
Supplement: Supplementary file 5 — Additional file 5. Implementation principles. [file 12916_2023_3102_MOESM5_ESM.docx]

# Additional File 5. Implementation Principles.

| **Implementation principle** | **Programme theories** | **Key dynamic addressed** |
| --- | --- | --- |
| 1. Organisational reach | **CMOC 41.** If an intervention is delivered to as many staff as possible (C), then it may be more effective at reducing UB (O), because it can improve engagement, interprofessional interactions, reduce ostracisation, and demonstrate that management is serious about tackling UB (M). | Key Dynamic 7. Interventions must be perceived as authentic to foster trust in management.   - *Reach can ensure that interventions are taken more seriously.*   Key Dynamic 10. Interventions that are both inclusive and equitable are critical to ensure effectiveness and sustainability and for addressing inequalities.   - *Including all groups of staff and not excluding any can help this dynamic.* |
| 1. Co-creation with staff | **CMOC 42**. If UB interventions are designed and implemented with staff at the helm (i.e., co-created) (C), then staff feel a sense of ownership (O), because they have been engaged with the process and perceive it to be authentic (M). | Key Dynamic 7. Interventions must be perceived as authentic to foster trust in management.   - *Co-creating with staff can help improve sense an intervention is authentic.*   Key Dynamic 1. Interventions need to address systemic factors that contribute to UB not only individual factors.   - *Working more closely with staff can help identify contributors to UB.* |
| 1. Assess organisational landscape before implementation | **CMOC 43**. If delivering an intervention to reduce UB (C), then assessing the organisational landscape in terms of culture, presence, and types of UB before the intervention is implemented can improve effectiveness (O), because it enables the specific targeting of appropriate strategies at contributors (M). | Key Dynamic 1. Interventions need to address systemic factors that contribute to UB not only individual factors.   - *Assessing the contributors to UB can help ensure an intervention targets the actual contributors to UB.*   Key Dynamic 7. Interventions must be perceived as authentic to foster trust in management.   - *Addressing contributors known to staff helps increase perception an intervention is genuine.*   Key Dynamic 8. One size does not fit all – tackling UB generally requires multiple and sustained interventions to address underlying contributors.   - *Ensuring the landscape is understood can improve knowledge of the scope of the required intervention.* |
| 1. Dedicated staff to lead work to tackle UB | **CMOC 44**. If delivering an intervention to reduce UB (C), then having dedicated staff in place to tackle UB can improve intervention momentum and sustainability (O), because staff can collate relevant information for implementing the intervention which may enhance applicability of content, increase perception that management is serious about addressing UB and increase staff engagement (M). | Key Dynamic 7. Interventions must be perceived as authentic to foster trust in management.   - *Drawing on dedicated staff can increase sense of authenticity.*   Key Dynamic 3: How and why an intervention is expected to work must be clear otherwise evaluations of interventions can be misleading.   - *Use of dedicated staff can provide resource for a better-designed evaluation.* |
| 1. Skilled facilitation for training | **CMOC 45**. If an intervention to reduce UB relies on facilitation (C), then the facilitator must have adequate training and be skilled at delivering intervention content to be successful (O), because a facilitator with the ‘right’ skills will be capable of building an alliance between themselves and participants, keeping discussion on track and engaging participants, leading to better intervention outcomes (M). | Key Dynamic 7. Interventions must be perceived as authentic to foster trust in management.   - *Having skilled facilitation ensures intervention content is imparted properly and helps avoid unintended consequences arising from misunderstandings.* |
| 1. Multiple simultaneous strategies | **CMOC 46**. If an intervention draws on a greater number of simultaneous strategies (C), then it may have a greater culture change impact (O), because they create social systems that promote behaviour change, reach more people, increase uptake, address multiple contributors, and send a signal that management is serious about addressing UB (M). | Key Dynamic 7. Interventions must be perceived as authentic to foster trust in management.   - *Use of multiple strategies improves authenticity of a programme.*   Key Dynamic 8. One size does not fit all – tackling UB generally requires multiple and sustained interventions to address underlying contributors.   - *Using multiple strategies increases the chance that participants will be impacted by the programme.* |
| 1. Maximise visibility across the organisation | **CMOC 47**. If delivering an intervention to reduce UB (C), then ensuring it is highly visible through advertising improves its momentum and effectiveness (O), because it helps engage staff, sends a signal that culture change is happening and increases participation (M). | Key Dynamic 7. Interventions must be perceived as authentic to foster trust in management.   - *Maximising visibility improves the perception the intervention is authentic.* |
| 1. Early intervention | **CMOC 48**. If a pattern of UB is identified in an organisation (C), then it is important to demonstrate that something is being done to address it rapidly (even if working on implementing a more complex intervention alongside a smaller rapid action) to ensure it does not take hold (O) because doing so indicates that management will not tolerate any UB and mitigates loss of trust in management (M). | Key Dynamic 7. Interventions must be perceived as authentic to foster trust in management.   - *Intervening early helps avoid any further loss of trust in management, improving engagement with later efforts to reduce UB.* |
| 1. Manager and leader engagement | **CMOC 49**. If implementing an intervention to reduce UB (C), then ensuring senior staff (i.e. managers and leaders) are engaged (e.g. role model behaviour, provide resources and focus) is crucial to maintaining engagement and effectiveness (O), because this transmits the message that UB is not tolerated and fosters psychological safety (M). | Key Dynamic 7. Interventions must be perceived as authentic to foster trust in management.   - *Ensuring managers are seen to participate improves authenticity.* |
| 1. Intervention perceived as just and not punitive | **CMOC 50**. If seeking to implement an intervention to address UB (C), then ensuring the intervention is just and not punitive is essential to ensuring effectiveness of the intervention and avoiding backlash (O), because a sense of injustice can lead to disengagement with the intervention and generate a sense of outrage or discontent (M). | Key Dynamic 10. Interventions that are both inclusive and equitable are critical to ensure effectiveness and sustainability and for addressing inequalities.   - *Ensuring all groups perceive an intervention to be just and not unfair improves sense of equity.*   Key Dynamic 7. Interventions must be perceived as authentic to foster trust in management.   - *An unjust intervention can impact trust in management too.* |
| 1. Maximising existing opportunities | **CMOC 51**. If an organisation is seeking to reduce UB (C) then using existing organisational processes such as reviewing or appraising staff to discuss UB can build momentum (O) because using existing processes may enable other strategies to be more easily rolled out (M) | Key Dynamic 12. There are trade-offs between a theory-first and practice-first intervention design   - *Making use of existing processes used in practice can favour the advantages of a practice-first design.* |
| 1. Manage organisational turnover and change | **CMOC 52**. If implementing a longer-term intervention to reduce UB (C), then organisational change and turnover can disrupt intervention momentum (O), because there can be a loss in staff, expertise, resource and engagement (M) | Key Dynamic 7. Interventions must be perceived as authentic to foster trust in management.   - *If staff perceive an intervention as likely to not be sustained due to staff turnover this will lower sense of its authenticity.* |
| 1. Tackle the instigator of UB and not the victim | **CMOC 53**. If an individual is frequently engaging in UB, then moving the victim or instigator out of the situation (C) can lead to a worsening of social norms about UB (O), because it sends a signal that an organisation is tolerant of UB (M) | Key Dynamic 7. Interventions must be perceived as authentic to foster trust in management.   - *Moving a victim rather than tackling an instigator can send signals that instigators are tolerated. This can undermine trust in management.* |
| 1. Incorporate ongoing evaluation | **CMOC 54**. If implementing an intervention to tackle UB (C), then embedding an ongoing evaluation can enable strategies to be adjusted and adapted (O), because there can be a greater understanding of the organisational and implementation context (M). | Key Dynamic 11. There are trade-offs between fixed interventions and flexibility.   - *Ongoing evaluation enables better flexibility during programme implementation.*   Key Dynamic 8. One size does not fit all – tackling UB generally requires multiple and sustained interventions to address underlying contributors.   - *Enabling changes to be made during implementation can enhance ability to address underlying contributors, which impacts Key Dynamic 8.* |
| 1. Do not mix hierarchies | **CMOC 55.** When seeking to include employees at all levels of an organisation with managers and lower-level staff present when implementing interventions that rely on group sessions (C) this can cause disengagement with the intervention (O-) because speaking up can be inhibited due to mixing of hierarchies reducing psychological safety for lower-level staff (M) | Key Dynamic 4. Maintaining a focus on why it is important to reduce UB (e.g. to improve patient safety) is key when designing an intervention to reduce UB.   - *Mixing hierarchies can undermine sense of psychological safety which can impact the sense that it is safe to speak up.* |
